# A PP2A molecular glue overcomes RAS/MAPK inhibitor resistance in *KRAS*-mutant non–small cell lung cancer

**DOI:** 10.1172/JCI193790

**Published:** 2025-10-14

**Authors:** Brynne Raines, Stephanie Tseng-Rogenski, Amanda C. Dowdican, Irene Peris, Matthew Hinderman, Kaitlin P. Zawacki, Kelsey Barrie, Gabrielle Hodges Onishi, Alexander M. Dymond, Tahra K. Luther, Sydney Musser, Behirda Karaj Majchrowski, J. Chad Brenner, Aqila Ahmed, Derek J. Taylor, Caitlin M. O’Connor, Goutham Narla

**Affiliations:** 1 Cellular and Molecular Biology Program and; 2 Department of Internal Medicine, Division of Genetic Medicine, University of Michigan, Ann Arbor, Michigan, USA.; 3 Rogel Cancer Center, University of Michigan Health, Ann Arbor, Michigan, USA.; 4 Department of Pharmacology, Case Western Reserve University, Cleveland, Ohio, USA.; 5 Department of Pharmacology and; 6 Department of Otolaryngology, University of Michigan, Ann Arbor, Michigan, USA.; 7 Department of Biochemistry, Case Western Reserve University, Cleveland, Ohio, USA.

**Keywords:** Cell biology, Oncology, Drug therapy, Lung cancer, Tumor suppressors

## Abstract

The effectiveness of RAS/MAPK inhibitors in treating metastatic *KRAS*-mutant non–small cell lung cancer (NSCLC) is often hindered by the development of resistance driven by disrupted negative feedback mechanisms led by phosphatases like PP2A. PP2A is frequently suppressed in lung cancer to maintain elevated RAS/MAPK activity. Despite its established role in regulating oncogenic signaling, targeting PP2A with RAS/MAPK to prevent resistance has not been previously demonstrated. In this study, we aimed to establish a treatment paradigm by combining a PP2A molecular glue with a RAS/MAPK inhibitor to restore PP2A activity and counteract resistance. We demonstrated that KRASG12C and MEK1/2 inhibitors disrupted PP2A carboxymethylation and destabilized critical heterotrimeric complexes. Furthermore, genetic disruption of PP2A carboxymethylation enhanced intrinsic resistance to MEK1/2 inhibition both in vitro and in vivo. We developed RPT04402, a PP2A molecular glue that selectively stabilizes PP2A-B56α heterotrimers. In commercial cell lines and in a patient-derived model, combining RPT04402 with a RAS/MAPK inhibitor slowed proliferation and enhanced apoptosis. In mouse xenografts, this combination induced tumor regressions, extended median survival, and delayed the onset of treatment resistance. These findings highlight that promoting PP2A stabilization and RAS/MAPK inhibition presents a promising therapeutic strategy to improve treatment outcomes and overcome resistance in metastatic *KRAS*-mutant NSCLC.

## Abstract 2

Combining a PP2A molecular glue with RAS/MAPK inhibitors restores tumor-suppressor activity and overcomes drug resistance in metastatic KRas-driven lung cancer.

## Graphical abstract

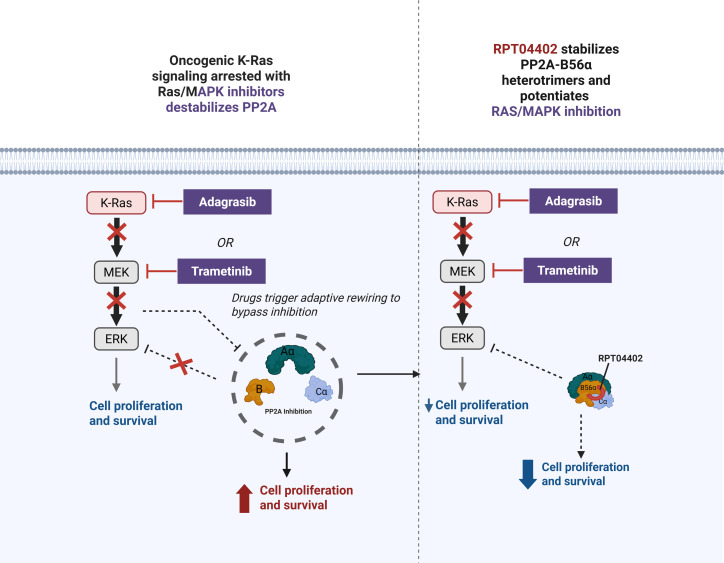

## Introduction

Oncogenic *KRAS* mutations are present in approximately 30% of non–small cell lung cancers (NSCLCs) (1, 2). Patients with tumors harboring these pathogenic mutations experience shorter median survival, partly due to late diagnosis and modest response to targeted therapies (3, 4). FDA approval of the KRASG12C-mutant-specific inhibitor adagrasib has shown promise clinically (5, 6); however, in a recent phase III trial of adagrasib, the objective response rate was only 32%, with a median progression-free survival of 5.5 months (7). It is likely that the poor objective response rate to adagrasib stems from intrinsic and acquired resistance mechanisms as in vitro studies have confirmed that cancer cells rapidly bypass KRASG12C inhibition if they respond at all (8–10). Deregulation and aberrant activation of the RAS/RAF/MEK/ERK (RAS/MAPK) pathway is a hallmark of *KRAS*-mutant NSCLC (11, 12). In addition to inhibiting oncogenic K-Ras, other treatment approaches have centered on the use of inhibitors targeting effector kinases such as MEK1/2. Although MEK1/2 inhibitors exhibit strong responses in preclinical models, long-term efficacy is limited in the clinic, and most patients develop resistance shortly after treatment initiation (13, 14). In several phase II and III trials, MEK1/2 inhibitor monotherapies failed to drive significant improvements in response rates or overall survival when compared with standard chemotherapy in patients with *KRAS*-mutant NSCLC (15, 16). These results suggest that resistance to targeted therapies remains the rule rather than the exception, highlighting the need to identify additional mechanisms of resistance and prevent them from developing.

Current targeted therapies for NSCLC are predominately inhibitors of oncogenic kinases; however, only targeting kinases ignores the critical role of phosphatases, like protein phosphatase 2A (PP2A), as regulators of protein phosphorylation in abating resistance mechanisms to RAS/MAPK inhibition (RAS/MAPKi). PP2A encompasses a family of serine/threonine phosphatases, where the active heterotrimeric forms are composed of a catalytic (Cα/β) subunit, scaffolding (Aα/β) subunit, and regulatory (B) subunit, the last of which drives its substrate specificity and ability to regulate diverse cellular processes and functions (17). Nominally, specific PP2A heterotrimers are critical tumor suppressors, and their inhibition is a prerequisite for the transformation of various cell types with activating *RAS* mutations (18–20). In fact, PP2A inhibition is a well-documented driver of lung cancer development and progression (21, 22). PP2A is also an important negative regulator of K-Ras–mediated signaling cascades including MAPK and PI3K/AKT/MTOR signaling; furthermore, hyperactivation of both cascades are known as resistance mechanisms (intrinsic and acquired) to KRASG12C and MEK1/2 inhibitors (23, 24).

PP2A heterotrimer biogenesis directs tumor-promoting or tumor-suppressive activity (15, 17). Formation of PP2A holoenzymes is regulated through unique reversible carboxymethylation of the L309 residue on PP2A C, acting as a “binding code” for B subunits. The methyl group is added by leucine methyltransferase 1 (LCMT1) and removed by the protein phosphatase methylesterase 1 (PME1). L309 carboxymethylation neutralizes the negative charge on the carboxyl-terminus of the flexible C-subunit tail, and in general, is required for the formation of tumor-suppressive PP2A holoenzymes. Loss of this posttranslational modification is commonly seen in cancer; in fact, low and high protein expression levels of LCMT1 and PME1, respectively, have been identified in cancers of the lung, prostate, and liver (25–27).

We have previously demonstrated that inhibition of PP2A, through mutation or siRNA-mediated knockdown of PP2A-Aα, contributes to MEK1/2 inhibitor resistance in *KRAS*-mutant colon and lung cancers (23, 28). Our group has also shown that the treatment of *KRAS*-mutant NSCLC with a PP2A molecular glue, DT-061, using both cell-based and in vivo models, induces apoptosis, inhibits tumor growth, and synergizes with the MEK1/2 inhibitor selumetinib (23, 29). These data suggest a role for PP2A signal rewiring that can bypass resistance; however, the mechanisms underlying these findings, namely synergy with a MEK1/2 inhibitor, and whether PP2A molecular glues can overcome resistance to MAPK inhibition more broadly, are unknown.

Given that PP2A is rarely mutated or lost in *KRAS*-mutant NSCLC, we sought to investigate alterations in the posttranslational modifications of PP2A upon RAS/MAPKi using KRASG12C and MEK1/2 inhibitors. We used a panel of *KRAS*-mutant NSCLC cell lines to demonstrate that clinically relevant RAS/MAPK inhibitors adagrasib (G12Ci) and trametinib (MEK1/2i) reduced carboxymethylation and destabilized PP2A heterotrimers and that combination treatment with a PP2A molecular glue, RPT04402, restabilized PP2A heterotrimers. Importantly, the combination resulted in enhanced in vitro and in vivo efficacy of adagrasib and trametinib. This work provides insights toward understanding the role of PP2A in adagrasib and trametinib treatment to improve the clinical outcomes of both inhibitors in metastatic *KRAS*-mutant NSCLC.

## Results

### KRASG12C and MEK1/2 inhibition decreases carboxymethylated PP2ACα and destabilizes PP2A heterotrimers.

**Figure 1:**
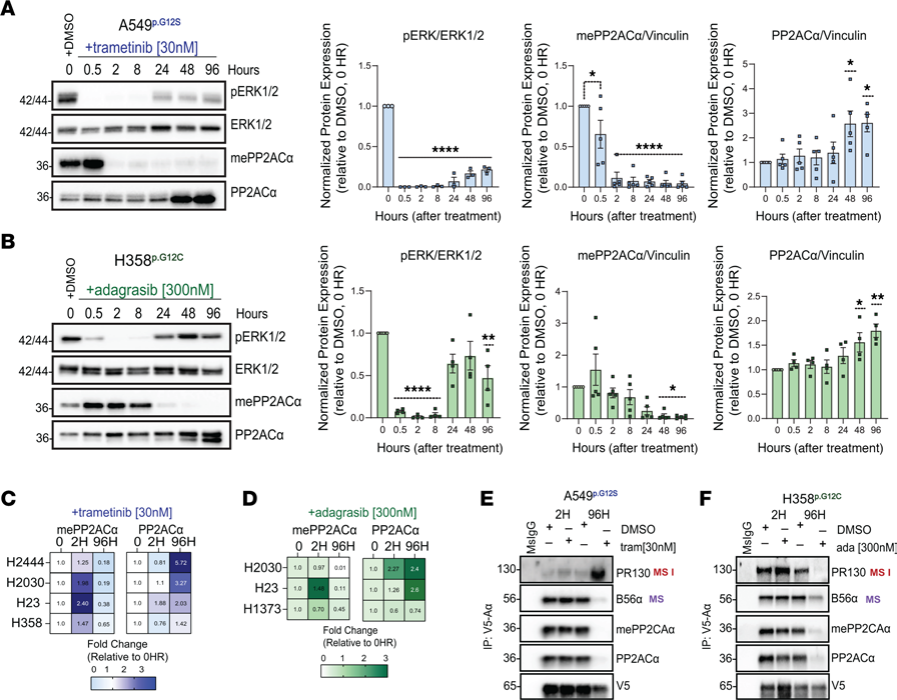
Inhibition of RAS/MAPK signaling in NSCLC reduces methylated PP2ACα and alters PP2A heterotrimer composition in vitro. (**A** and **B**) Western blot and quantification of pERK, ERK, mePP2ACα, and total PP2ACα in A549 cells treated with trametinib (30 nM) and NCI-H358 cells treated with adagrasib (300 nM) for the indicated time points. pERK was normalized to ERK; mePP2ACα and total PP2ACα were normalized to vinculin, and all targets are expressed relative to DMSO, 0 hours. Vinculin blots are shown in Supplemental Figure 2. Significance determined by 1-way ANOVA with Dunnett’s correction: *P ≤ 0.05, **P ≤ 0.01, ****P ≤ 0.0001. MS I, methylation-insensitive; MS, methylation-sensitive. (**C** and **D**) Heatmaps summarizing mePP2ACα and total PP2ACα levels in the indicated cell lines after trametinib or adagrasib treatment. Associated Western blots and quantification data are in Supplemental Figures 6 and 7 and the Supporting Data Values file. (**E** and **F**). Co-IP from A549 and H358 expressing V5-tagged PP2A-Aα showing altered holoenzyme composition 2 and 96 hours after trametinib or adagrasib treatment. Input controls are in Supplemental Figure 4; quantification is in Supplemental Figures 4 and 5. All data are presented as mean ± SEM (*n* ≥ 3).

Carboxymethylation is critically important to the formation and activity of tumor-suppressive PP2A heterotrimers. To determine whether RAS/MAPKi alters carboxymethylation, we evaluated carboxymethylated C subunit (mePP2ACα) expression in A549 and NCI-H358 NSCLC cell lines treated with trametinib or adagrasib. To determine the optimal drug concentrations able to decrease phosphorylated ERK (pERK) to undetectable levels, we treated cells with a range of drug concentrations (1–1,000 nM) (Supplemental Figure 1, A and C; supplemental material available online with this article; https://doi.org/10.1172/JCI193790DS1). We then set the in vitro trametinib concentration for further experiments to a dose of 30 nM, the lowest dose to maximally inhibit ERK signaling. In NCI-H358 cells, between 100 and 1,000 nM of adagrasib ablated pERK expression, and we selected a concentration of 300 nM for further studies. At these concentrations, we observed that trametinib and adagrasib induced time-dependent loss of mePP2ACα (Figure 1, A and B), which preceded the reactivation of pERK and hyperphosphorylation of AKT (pT308; pS473 AKT/t-AKT) (Figure 1, A and B, and Supplemental Figure 2). Therefore, the loss of mePP2ACα may contribute to known resistance programs in response to RAS/MAPKi. Total PP2ACα levels increased during the study, and the most significant changes occurred at the latest time points of 48 and 96 hours. This observation is consistent with previous studies, which suggest total PP2ACα protein levels increase when phosphatase activity is low (30). Similar effects on mePP2ACα and PP2ACα were detected across a panel of *KRAS*-mutant NSCLC cell lines treated with trametinib and adagrasib (Figure 1, C and D, and Supplemental Figures 6 and 7). Interestingly, there were no appreciable changes in the protein expression levels of LCMT1 or PME1 after RAS/MAPKi for any of the cell lines tested, suggesting that LCMT1 and PME1 enzymatic activity or interactions with the C-subunit may be regulated in response to treatment (Supplemental Figure 2, B and D, and Supplemental Figures 6 and 7).

We repeated the experiments using another set of chemically distinct inhibitors of MEK1/2 and KRASG12C, selumetinib (MEKi) and sotorasib (G12Ci). Again, we used optimal concentrations necessary to decrease pERK and those matched to relevant plasma exposures — 1 μM of selumetinib and 300 nM of sotorasib (Supplemental Figure 1, B and D). Both drugs reduced mePP2ACα and increased total PP2ACα expression (Supplemental Figure 3). These data indicate that RAS/MAPKi-induced changes in mePP2ACα and PP2ACα, independent of LCMT1 and PME1 expression, are not off-target products of trametinib and adagrasib but a product of general MEK1/2 or KRASG12C inhibition (Supplemental Figure 2, A and B, and Supplemental Figure 3, A and B). Given the loss of mePP2ACα, we assessed whether RAS/MAPKi altered the composition of PP2A heterotrimers. We stably overexpressed V5-tagged PP2A-Aα in A549 and NCI-H358 cells to immunoprecipitate PP2A complexes. After 96 hours of treatment with trametinib or adagrasib, we observed a dramatic reduction in the binding of the B56 family of regulatory subunits, including methylation-sensitive subunits like B56α and B56ε and some methylation-insensitive subunits like B56δ (Figure 1, E and F, and Supplemental Figures 4 and 5). Importantly, B56 regulatory subunit loss correlated with reduced binding between PP2A-Aα, mePP2ACα, and total PP2ACα, indicative of a disruption of AC dimerization. In A549 cells, the methylation-insensitive subunit PR130 increased binding to the scaffold, whereas binding between PP2A-Aα and PR130 decreased in NCI-H358 cells. Interestingly, we observed that trametinib, not adagrasib, upregulated PR130 levels in cells, which likely explains the enhanced binding between PR130 and the scaffold. Together, these data indicate that RAS/MAPKi altered PP2A heterotrimer composition and the availability of PP2A enzymes during the acquisition of resistance mechanisms in our experimental settings.

### Demethylated PP2ACα drives resistance to RAS/MAPKi in vitro.

**Figure 2:**
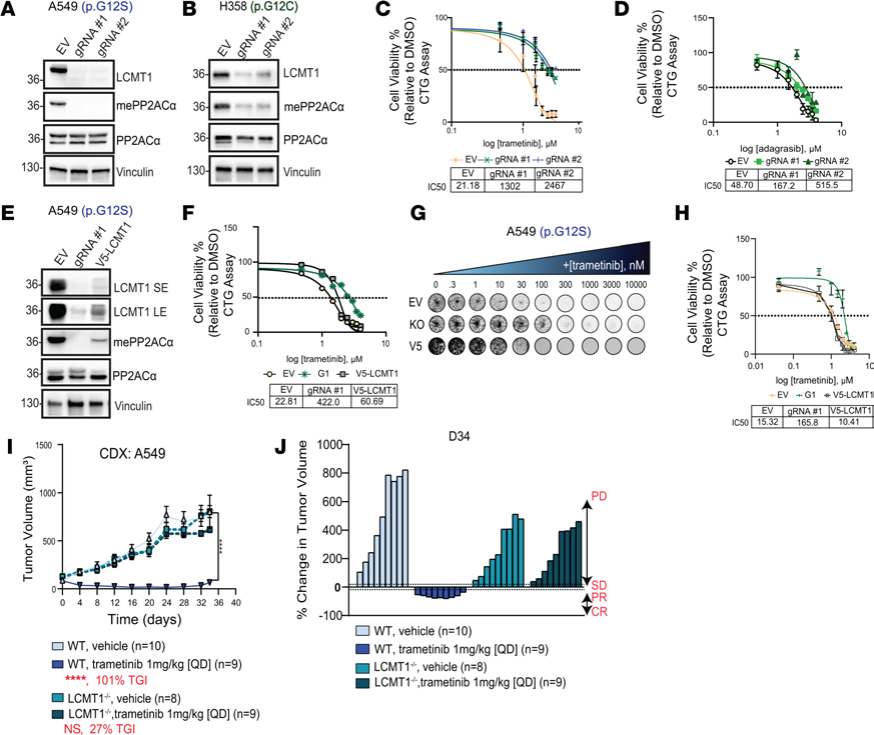
Demethylation of PP2ACα drives intrinsic resistance to RAS/MAPK inhibition in vitro and MEK1/2 inhibition in vivo. (**A** and **B**) Western blot of LCMT1, mePP2ACα, and PP2ACα in A549 and NCI-H358 cells expressing empty vector (EV) or LCMT1^–/–^ using 2 gRNAs. (**C** and **D**) Cell viability of A549 and NCI-H358 EV or LCMT1^–/–^ cells treated with trametinib or adagrasib (0–10 μM, 72 hours). (**E**) Western blot of A549 EV, LCMT1^–/–^, and V5-tagged LCMT1 (reconstitution) cells. (**F**) Corresponding trametinib cell viability (CTG) assay (0–10 μM, 72 hours). (**G** and **H**) Clonogenic assay of A549 EV, LCMT1^–/–^, and V5-tagged LCMT1 cells treated with trametinib (0–10 μM, 1.5 weeks), with growth inhibition (%) calculated in **H**. (**I**) Tumor growth curves for A549 EV and LCMT1^–/–^ CDXs treated with vehicle or trametinib (1 mg/kg, QD). TGI was assessed at day 34. (**J**) Waterfall plot showing tumor response by RECIST: PD, progressive disease; SD, stable disease; PR, partial response; CR, complete response. All Western blot quantifications are in Supplemental Figure 8, and raw data are in Supporting Data Values file. One-way ANOVA with Tukey’s multiple-comparison test was used to calculate statistical significance in **I**: ****P ≤ 0.0001.

To examine whether the loss of mePP2ACα could affect the drug response of RAS/MAPK inhibitors and drive resistance, we aimed to abolish carboxymethylation in A549 and NCI-H358 cells. We generated *LCMT1^–/–^* (LCMT1) KO or empty vector (EV) control lines using CRISPR/Cas9 (Figure 2, A and B, and Supplemental Figure 8, A and C). In A549 cells, we achieved undetectable levels of LCMT1 using 2 independent guide RNAs (gRNAs), resulting in a significant loss of mePP2ACα compared with the EV control (Figure 2A and Supplemental Figure 8, A and B). To determine whether the KO cells produced a differential response to MEKi, we treated EV and KO cells with increasing doses of trametinib for 72 hours and measured cell viability by CellTiter-Glo (CTG) assay. Both KO lines displayed increased resistance through an IC_50_ shift from 21.18 nM to 1 μM or greater in the KO cells (Figure 2C). Compared with the effects of LCMT1 KO on trametinib, adagrasib responses were more modest, possibly due to incomplete LCMT1 loss and retention of methylation in NCI-H358 cells (Figure 2B and Supplemental Figure 8, C and D). The KO lines displayed increased IC_50_ values compared with EV — 3-fold and 11-fold individually (Figure 2D).

To assess whether the observed effect on drug response was a result of LCMT1^–/–^ specifically, we restored LCMT1 expression using a V5-tagged LCMT1-expressing construct in A549 cells. Briefly, we lentivirally transduced A549 gRNA #1 LCMT1^–/–^ cells with a V5-tagged LCMT1 (V5-LCMT1) plasmid containing a mutated PAM site such that it could not be recognized by Cas9. Restoration of LCMT1 and mePP2ACα were confirmed by Western blot (Figure 2E and Supplemental Figure 8, E and F). The reconstituted cells were then subjected to a CTG assay for 72 hours and clonogenic assay for 2 weeks. The results showed that the reconstitution of LCMT1 into the KO cells reversed intrinsic resistance to trametinib. For the CTG assay, the restoration of LCMT1 led to a 7-fold reduction in the IC_50_ (422 nM to 61 nM) (Figure 2F). By clonogenic assay, we determined that the LCMT1^–/–^ cells were more resistant to trametinib compared with the EV control because after 2 weeks of treatment, 50% growth inhibition (GI_50_), defined as the concentration of drug required to reduce colony formation by 50% relative to untreated controls, was observed at a higher dose. The restoration of LCMT-1 led to a 10-fold or greater reduction in the GI_50_ to EV levels (165.8 nM to 10.41 nM) (Figure 2, G and H).

Together, these data suggest that decreased mePP2ACα caused by LCMT1^–/–^ drives intrinsic resistance to RAS/MAPKi — more so trametinib than adagrasib. Therefore, a decrease in mePP2ACα in response to RAS/MAPKi, described above, contributed to the cellular response, resulting in acquired resistance to these inhibitors (Figure 1, A and B, and Supplemental Figures 6 and 7).

### Demethylated PP2ACα drives resistance to MEK inhibition in vivo.

To determine whether decreased mePP2ACα drives intrinsic resistance to trametinib in vivo, we generated A549 EV and LCMT1 gRNA#1 cell-derived xenografts (CDXs). First, 5 × 10^6^ cells were injected subcutaneously into the right flank of male nude mice. After tumors reached 100 mm^3^, mice were randomized into 2 treatment groups: vehicle or trametinib (1 mg/kg) treated once per day (QD) by oral gavage. The dose and timing of treatment were determined based on the literature for in vivo studies using trametinib (31). Tumor volumes were measured twice per week by caliper measurement. Consistent with our cell-based studies, LCMT1^–/–^ trametinib tumors were indistinguishable from the vehicle tumors in that they did not respond to trametinib and had a tumor growth inhibition (TGI) of 27%. EV tumors treated with trametinib presented a TGI of 101% compared with vehicle controls and experienced regressions (Figure 2, I and J). We confirmed KO of LCMT1 and loss of mePP2ACα in the tumors at the conclusion of the study by Western blot (Supplemental Figure 8G). Importantly, the KO of LCMT1 and trametinib were well-tolerated as assessed by body weight (Supplemental Figure 8F).

At the end of the study, we found that EV-vehicle tumors were heavier than the EV-trametinib tumors, suggesting that trametinib was effective in this model. Of note, the LCMT1^–/–^ tumors (vehicle and trametinib) showed more necrosis and tumor ulcerations compared with the EV controls, making it challenging to draw a definitive conclusion for the pair. Together, the data suggest that the inactivation of PP2A through a reduction of mePP2ACα can lead to resistance to MEK1/2 inhibition in vitro and in vivo (Supplemental Figure 8H).

### PP2A-B56α loss confers resistance to RPT04402 and RAS/MAPKi in KRAS-mutant NSCLC cells.

The treatment of NSCLC cells with a RAS/MAPKi not only affects mePP2ACα expression level but also PP2A heterotrimer stability, which requires it. Certain methylation-sensitive heterotrimers, namely PP2A-B56α, negatively regulate MAPK and AKT/mTOR signaling (10, 32). Based on this, we hypothesized that the loss of PP2A-B56α observed in previous studies (Figure 1, E and F) could drive RAS/MAPKi resistance and that preventing this loss could delay it. To address this, we developed a PP2A molecular glue, RPT04402, that selectively stabilizes PP2A-B56α heterotrimers. RPT04402 increases the affinity of B56α for the A/C dimer, and this interaction is enhanced when mePP2ACα is reduced (Derek J. Taylor, Case Western Reserve University, unpublished observations). Therefore, combining RPT04402 with a RAS/MAPKi should stabilize PP2A-B56α heterotrimers and bypass resistance mechanisms induced by RAS/MAPKi.

We performed single-agent experiments to evaluate the efficacy of RPT04402, trametinib, and adagrasib in promoting cytotoxicity and inhibiting proliferation. In 2D and 3D cell viability assays, RPT04402 triggered cell death in a dose-dependent manner, with no difference in IC_50_ values between the 2 settings (Supplemental Figure 9, A and B). Adagrasib showed variable sensitivity, inducing cytotoxicity in NCI-H358 but not in NCI-H2030 or 6652CL cells (Supplemental Figure 9, C and D). 6652CL, created from a *KRAS* G12C-mutant patient-derived xenograft, was the most resistant to adagrasib treatment. This result is in line with literature highlighting variable, often ineffective, responses to direct G12C inhibition in commercial and patient-derived models. Trametinib mainly induced cytostatic responses, with a moderately cytotoxic response in A549 cells and minimal effects in others (Supplemental Figure 9, E and F). In clonogenic assays, all drugs inhibited proliferation in a dose-dependent manner (Supplemental Figure 9, G and H).

To evaluate the functional specificity of RPT04402 for PP2A-B56α, we generated B56α-KO (B56α^–/–^) A549 and NCI-H358 cell lines using CRISPR/Cas9 (Supplemental Figure 10, A–D). Clonogenic assays were employed to investigate specificity, as RPT04402 exhibited a more pronounced antiproliferative effect compared with its impact on cell viability. In our clonogenic assays, B56α loss impaired the ability of trametinib and RPT04402 to inhibit cell proliferation. In A549 cells, B56α^–/–^ increased resistance to trametinib (GI_50_ increased 8-fold) and RPT04402 (GI_50_ doubled). Similarly, in NCI-H358 cells, B56α^–/–^ increased resistance to both adagrasib and RPT04402 (GI_50_ for RPT04402 increased from 48.19 μM to 810.3 μM). These data indicate that the effects of both trametinib and RPT04402 on cell proliferation are partially dependent on B56α in vitro (Supplemental Figures 10, E–J).

### RPT04402 synergizes with MEK1/2 and KRASG12C inhibition to trigger cell death in NSCLC.

**Figure 3:**
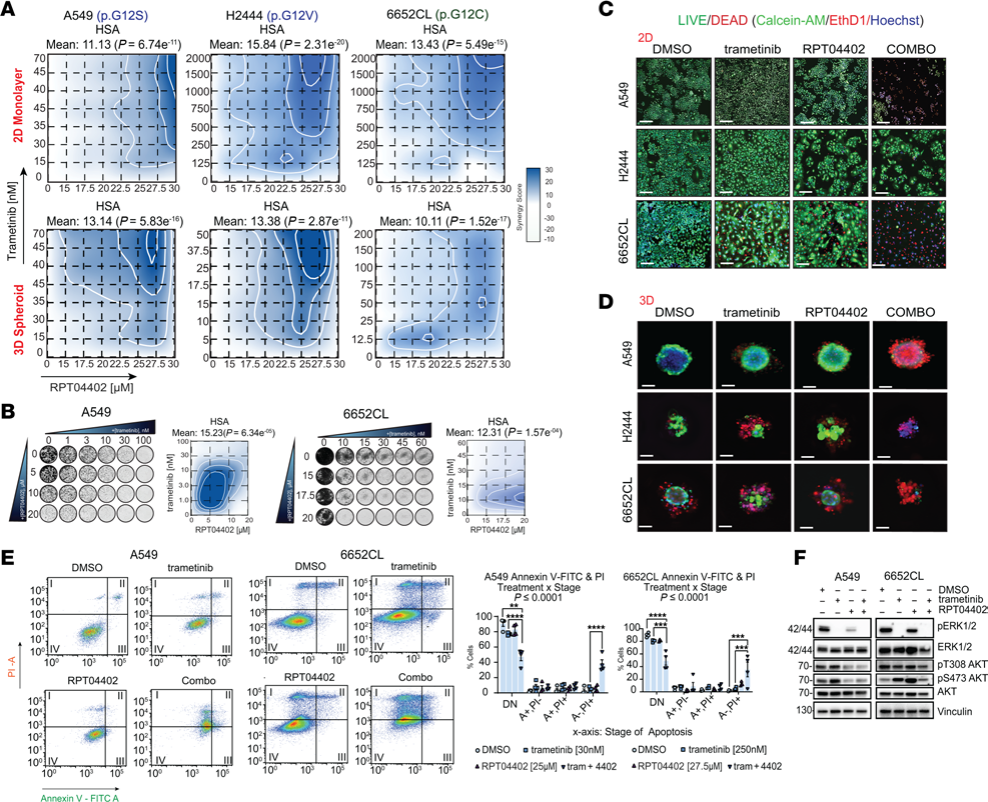
RPT04402 enhances the effects of trametinib by increasing cytotoxicity, reducing cell proliferation, and promoting apoptosis/cell death in NSCLC. (**A**) 2D contour plots of highest single-agent synergy scores for cell lines (A549, NCI-H2444, 6652CL) cultured as 2D monolayers or 3D spheroids. Cells were treated with increasing concentrations of RPT04402 or trametinib, either as single treatments or in combination, for 48 hours. (**B**) Clonogenic assay of A549 and 6652CL cells treated with increasing concentrations of trametinib, RPT04402, or the combination, and cultured for 2 weeks. (**C** and **D**) Representative images of 2D and 3D live/dead staining in A549 (trametinib: 2D/3D = 30 nM; RPT04402: 2D/3D = 25 μM), NCI-H2444 (trametinib: 2D/3D = 250/50 nM; RPT04402: 2D/3D = 25 μM), and 6652CL (trametinib: 2D/3D = 250 nM; RPT04402: 2D/3D = 25 μM) cells after 48 hours of treatment with trametinib, RPT04402, or the combination. Scale bar: 75 μm. (**E**) Annexin V/propidium iodide double staining assay of A549 (trametinib: 30 nM; RPT04402: 25 μM) and 6652CL (trametinib: 250 nM; RPT04402: 25 μM) cells treated with trametinib, RPT04402, or the combination for 48 hours. Data quantification is shown as bar graphs with individual data points and the mean ± SE (*n* ≥ 3). Statistical significance was assessed by 2-way ANOVA with Tukey’s post hoc analysis. (**F**) Western blot analysis of pERK and pAKT (pT308 and pS473) levels, normalized to tERK and tAKT, respectively, in A549 and 6652CL cells from the annexin V/propidium iodide assay. Blot quantifications are in Supplemental Figure 11. Raw values from all studies are in the Supporting Data Values file.

To determine whether RPT04402 could enhance MEK1/2 inhibition in vitro, we evaluated the synergistic effect of RPT04402 and trametinib in A549, NCI-H2444, and 6652CL. Cell viability assays showed that RPT04402 synergized with trametinib to enhance cytotoxicity after 48 hours of treatment, as determined by the highest single-agent synergy model (≥ 10 = synergism) (Figure 3A). This model assumes a positive interaction when the combination of drugs induces a greater response than single-agent treatments. Trametinib and RPT04402 induced modest cell death (≤ 50%) as single-agent treatments but enhanced cytotoxicity by at least 80% when combined (Supplemental Figure 11A). Similar synergy profiles were observed in both 2D and 3D contexts, indicating no effect of the cell culture vessel on the phenotype (Figure 3A and Supplemental Figure 11). To assess the durability of the combinatorial activity between the 2 drugs, A549 and 6652CL were treated with trametinib, RPT04402, or the combination for 2 weeks. Both cell lines showed dose-dependent inhibition of colony growth as single-agent treatments (Figure 3B and Supplemental Figure 11, B and C). The combination of RPT04402 and trametinib inhibited cell proliferation, and there were no signs of regrowth (relative to control) in the combination-treated wells in either cell line (Figure 3B). The synergistic effect on cell death, as measured by CTG, was confirmed by live/dead staining in 2D and 3D conditions after treating cells for 48 hours. In both contexts, the combination groups produced a higher percentage of dead cells compared with either trametinib or RPT04402 alone in A549 and 6652CL cells. However, we did not observe any increase in cell death in the combination groups in NCI-H2444 cells (Figure 3, C and D). These findings highlight the context-dependent nature of drug responses, particularly in *KRAS* G12V-mutant cancers, which are known to exhibit variable sensitivity to MEK1/2 inhibition (33–35). Additionally, our live/dead study was based on the “most synergistic” combination of drugs from the in vitro synergism studies; therefore, the combination identified in that context may not elicit the same cytotoxic effect in our live/dead system.

Next, we carried out annexin V/propidium iodide staining by flow cytometry to further examine the drug effects on apoptosis/cell death. A549 and 6652CL cells were treated for 48 hours with a combination of concentrations that produced the greatest amount of synergy in the 2D CTG assays (Supporting Data Values file). In both cell lines, single treatment of trametinib and RPT04402 did not significantly increase annexin V staining. However, the combination of trametinib and RPT04402 caused more than 50% of the cells to enter a late apoptotic/necrotic state (annexin V–negative, propidium iodide–positive) (Figure 3E). In both cell lines, we observed a statistically significant interaction effect (*P* ≤ 0.0001) and post hoc significance between trametinib and the combination (A549: *P* ≤ 0.0001; 662CL: *P* = 0.001). These data indicate that RPT04402 synergizes with trametinib to promote apoptosis/cell death.

**Figure 4:**
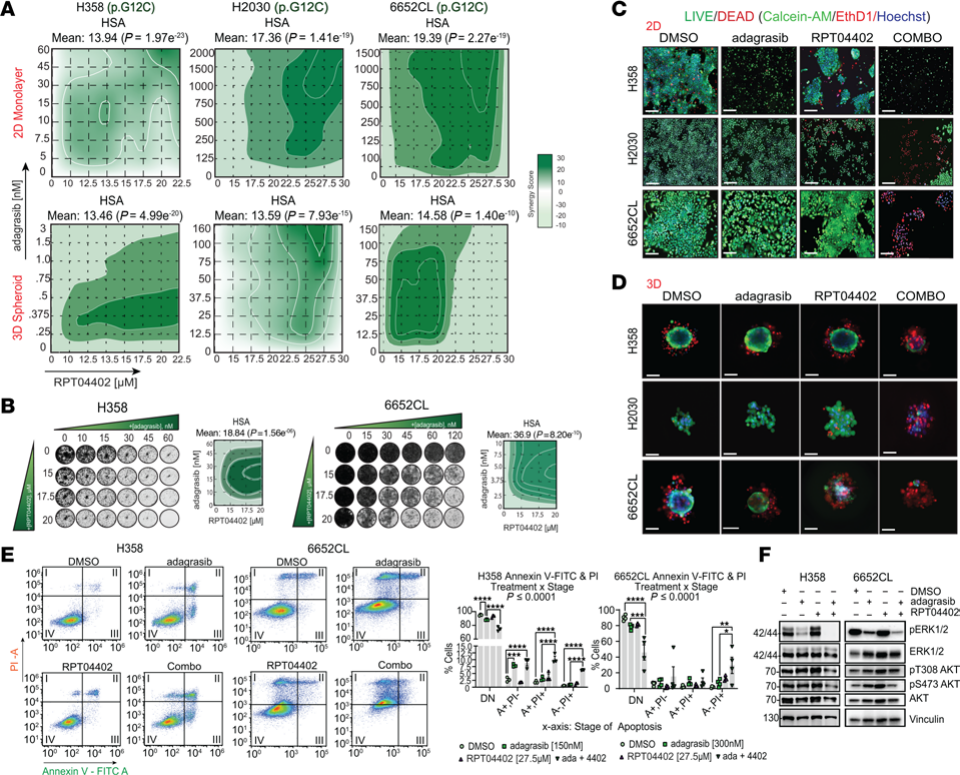
RPT04402 enhances the effects of adagrasib by increasing cytotoxicity, reducing cell proliferation, and promoting apoptosis/cell death in NSCLC. (**A**) 2D contour plots of highest single-agent synergy scores for cell lines (NCI-H358, NCI-2030, 6652CL) cultured as 2D monolayers or 3D spheroids. Cells were treated with increasing concentrations of RPT04402 or adagrasib, either as single treatments or in combination, for 48 hours. (**B**) Clonogenic assay of NCI-H358 and 6652CL cells treated with increasing concentrations of adagrasib, RPT04402, or the combination, and cultured for 2 weeks. (**C** and **D**) Representative images of 2D and 3D live/dead staining in NCI-H358 (adagrasib: 2D/3D = 150/3 nM; RPT04402: 2D/3D = 22.5 μM), NCI-H2030 (adagrasib: 2D/3D = 250 nM; RPT04402: 2D/3D = 27.5 μM), and 6652CL (adagrasib: 2D/3D = 250 nM; RPT04402: 2D/3D = 27.5 μM) cells after 48 hours of treatment with adagrasib, RPT04402, or the combination. Scale bar: 75 μm. (**E**) Annexin V/propidium iodide double staining assay of NCI-H358 (adagrasib: 150 nM; RPT04402: 25 μM) and 6652CL (adagrasib: 250 nM; RPT04402: 27.5 μM) cells treated with adagrasib, RPT04402, or the combination for 48 hours. Data quantification is shown as bar graphs with individual data points and the mean ± SE (*n* ≥ 3). Statistical significance was assessed by 2-way ANOVA with Tukey’s post hoc analysis. (**F**) Western blot analysis of pERK, pAKT (pT308 and pS473) levels, normalized to tERK and tAKT, respectively, in NCI-H358 and 6652CL cells from the annexin V/propidium iodide assay. Blot quantifications are in Supplemental Figure 12. The annexin V/propidium iodide plots for 6652CL treated with DMSO or RPT04402 are shared between Figures 3 and 4 given that all treatment groups were run simultaneously. Quantifications account for all pairwise comparisons made, and statistics were performed as described in Figure 3. Raw values from all studies are in the Supporting Data Values file.

We repeated our synergy experiments with adagrasib and RPT04402 in NCI-H358, NCI-H2030, and 6652CL cells. The combination exhibited synergistic effects in both 2D and 3D cultures (Figure 4A, Supplemental Figure 12A, and Supporting Data Values file). As noted previously, NCI-H2030 and 6652CL cells are intrinsically resistant to adagrasib compared with NCI-H358 cells (Supplemental Figure 9, C and D). When used alone, adagrasib resulted in IC_50_ values of 1 μM or higher in both resistant cell lines, but combining it with RPT04402 reduced the IC_50_ by more than 8-fold (Supplemental Figure 12A), suggesting that RPT04402 sensitizes resistant cells to adagrasib. This synergy was further supported by clonogenic assays, where NCI-H358 and 6652CL cells were treated with adagrasib, RPT04402, or their combination for 2 weeks. Both adagrasib and RPT04402 inhibited colony growth in a dose-dependent manner (Figure 4B). In NCI-H358 cells, RPT04402 enhanced the antiproliferative effect of adagrasib but only at lower concentrations of adagrasib (Supplemental Figure 12B). In contrast, adagrasib and RPT04402 alone induced minimal growth inhibition in 6652CL cells, while their combination increased cytotoxicity 80% or more, with the GI_50_ of adagrasib decreasing by 8-fold or greater when combined with RPT04402 at multiple concentrations (Supplemental Figure 12C).

Like our trametinib and RPT04402 studies, we confirmed synergy through live/dead staining in both 2D and 3D cultures. At the most synergistic concentrations, RPT04402 enhanced adagrasib’s cytotoxic effect in NCI-H358 cells and sensitized NCI-H2030 and 6652CL cells, which showed minimal cytotoxicity with adagrasib alone (Figure 4, C and D).

Annexin V/propidium iodide staining was performed 48 hours after treatment with the most synergistic concentrations in both cell lines. In NCI-H358 cells, adagrasib induced apoptosis, and the addition of RPT04402 further increased the percentage of double-positive (annexin V–positive, propidium iodide–positive) cells and of cells in a necrotic state (annexin V–negative, propidium iodide–positive). (Figure 4E). In 6652CL cells, treatment with adagrasib or RPT04402 alone modestly increased the percentage (≤ 20%) of cells in a late apoptotic/necrotic state. However, the combination treatment shifted more than 50% of the cells into this state (Figure 4E). In both cases, we observed a statistically significant interaction effect (*P* ≤ 0.0001) and post hoc significance between adagrasib and the combination (H358: *P* ≤ 0.0001; 662CL: *P* = 0.03).

Next, we aimed to determine how the addition of RPT04402 to either trametinib or adagrasib affected ERK and AKT pathway activation. In A549 and 6652CL cells, we observed that RPT04402 decreased pERK levels compared with the DMSO control in both cell lines (Figure 3F and Supplemental Figure 11, D and E). Interestingly, the combination of trametinib and RPT04402 also reduced pAKT (pT308 only in A549 cells and pS473 in both) compared with trametinib alone (Figure 3F and Supplemental Figure 10, D and E). The results with adagrasib and RPT04402 were more variable. In NCI-H358 cells, pERK levels rebounded 48 hours after adagrasib treatment; however, the addition of RPT04402 did not further suppress pERK to a level that reached statistical significance (Figure 4F and Supplemental Figure 11D). RPT04402 reduced pAKT hyperactivation, specifically at pS473 (but not pT308). In 6652CL cells, both drugs significantly decreased pERK compared with the DMSO control. Unlike the trametinib and RPT04402 combination, RPT04402 did not affect pAKT in 6652CL cells, as pAKT was not hyperphosphorylated at the selected concentration (Figure 4F and Supplemental Figure 11E). The variable effects on pERK and pAKT, despite the observed synergy between RPT04402 and either trametinib or adagrasib, suggest that the cooperation of these drugs extends beyond these 2 substrates.

### Synergism between RPT04402 and RAS/MAPK inhibitors depends on PP2A-B56α in vitro.

**Figure 5:**
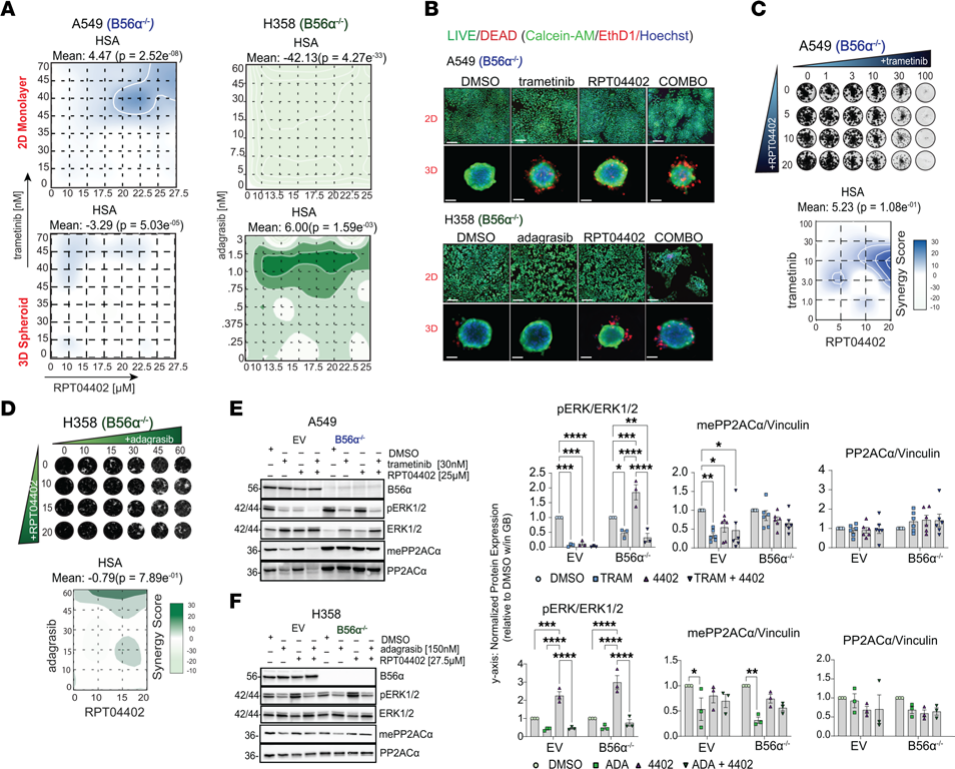
Loss of B56α abolishes synergy between RPT04402 and RAS/MAPK inhibition. (**A**) 2D contour plots of highest single-agent synergy scores for B56α^–/–^ cell lines (A549, NCI-H358) cultured as 2D monolayers or 3D spheroids. Cells were treated with increasing concentrations of RPT04402, trametinib (A549), or adagrasib (NCI-H358), either as single treatments or in combination, for 48 hours. (**B**) Representative images of 2D and 3D live/dead staining in A549 (trametinib: 2D/3D = 30 nM; RPT04402: 2D/3D = 25 μM) and NCI-H358 (adagrasib: 2D/3D = 150/3 nM; RPT04402: 2D/3D = 22.5 μM) cells after 48 hours of treatment. Scale bar: 75 μm. (**C**) Clonogenic assay of A549 cells treated with increasing concentrations of trametinib, RPT04402, or the combination, cultured for 2 weeks. (**D**) Clonogenic assay of NCI-H358 cells treated with increasing concentrations of adagrasib, RPT04402, or the combination, and cultured for 2 weeks. (**E**) Western blot analysis of pERK, mePP2ACα, and PP2ACα in A549 EV and B56α^–/–^ cells after 48 hours of treatment with trametinib (30 nM), RPT04402 (25 μM), and the combination. (**F**) Western blot analysis of pERK, mePP2ACα, and PP2ACα in NCI-H358 EV and B56α^–/–^ cells after 48 hours of treatment with adagrasib (150 nM), RPT04402 (27.5 μM), and the combination. For both **E** and **F**, pERK is normalized to tERK and mePP2ACα, and PP2ACα is normalized to the loading control, vinculin. Densitometry data are expressed relative to the DMSO condition within the genetic background. Vinculin blots are in Supplemental Figure 13. Data in graphs are represented as mean ± SEM (*n* ≥ 3). Statistical significance was determined by 1-way ANOVA with Tukey’s post hoc analysis: *P ≤ 0.05, **P ≤ 0.01, ***P ≤ 0.001, ****P ≤ 0.0001. Raw values are in the Supporting Data Values file.

Next, we investigated whether the synergy between RPT04402 and trametinib or adagrasib could be affected by B56α loss. We repeated our cell viability and clonogenic assays in B56α^–/–^ A549 and NCI-H358 cells. Briefly, B56α^–/–^ cells were treated with increasing concentrations of RPT04402, trametinib, or adagrasib for 72 hours in a CTG assay (Figure 5A). B56α^–/–^ reduced synergy scores in all settings (2D: 11.13 to 4.47 in A549 and 13.94 to –42.13 in H358; 3D: 13.14 to –3.29 in A549 and 13.46 to 6.00 in H358). In both cell lines, all drugs exhibited reduced effects on cell death, with some concentrations increasing viability compared with the DMSO control (NCI-H358) (Supplemental Figure 13, A and B). In addition, 2D and 3D live/dead staining confirmed the results of our CTG assay. For example, the combination of trametinib and RPT04402 in A549 B56α^–/–^ cells diminished, but not entirely abolished, synergy; fewer cells were observed in the combination group, indicating greater growth suppression than single-agent treatment (Figure 5B). Similarly, B56α^–/–^ did not entirely block the synergy between RPT04402 and adagrasib in NCI-H358 cells (Figure 5B).

In contrast with the CTG assays, B56α^–/–^ abolished synergy in our long-term clonogenic assays. In both cell lines, we found that the RAS/MAPK inhibitors and RPT04402 were unable to induce antiproliferative effects in the KO cells at the same concentrations as in parental cells, eliminating observed synergy in A549 (15.23 vs. 1.78) and NCI-H358 (18.84 vs. –0.79) (Figure 5, C and D).

Next, we examined the effects of the drug treatments on mePP2ACα, pERK, and pAKT in the KO cells compared with the EV control. In A549 EV cells, trametinib reduced mePP2ACα and increased total PP2ACα levels, consistent with our initial experiments (Figure 5E and Figure 1A). The reduction of mePP2ACα was also observed in the KO cells, indicating that the loss of B56α does not affect trametinib’s ability to directly alter mePP2ACα levels. Additionally, no changes were observed in pERK expression; for instance, single-agent trametinib reduced pERK, but the combination did not further suppress it, consistent with our parental results (Figure 5E and Figure 3F). In contrast to the parental and EV cells, the combination of trametinib and RPT04402 did not reduce hyperphosphorylation of AKT at either the pT308 or pS473 sites in the KO cells (Supplemental Figure 13F).

A similar pattern was observed in NCI-H358 cells, where both EV and KO cells showed a reduction in mePP2ACα in response to adagrasib treatment. No changes in pERK expression were observed (e.g., single-agent RPT04402 did not reduce pERK, nor did the combination treatment further enhance suppression) (Figure 5F). Furthermore, no effects on pAKT (pT308 or pS473) were detected in response to any of the drug treatments, whether administered as single agents or in combination (Supplemental Figure 13G).

### RPT04402 promotes TGI in KRAS-mutant NSCLC.

We have demonstrated that RPT04402 induces an antitumor response that is B56α dependent in endometrial cancer models (Irene Peris, University of Michigan, unpublished observations). To examine whether this dependency held true in *KRAS*-mutant NSCLC, we tested RPT04402 in the A549 CDX model, using both EV and B56α^–/–^ tumors (Supplemental Figure 14F). After tumors reached 100 mm^3^, mice were treated with 10 mg/kg or 100 mg/kg RPT04402 (oral gavage; QD). In the EV group, 10 mg/kg and 100 mg/kg RPT04402 treatment led to 59% TGI (*P* = 0.0271) and 42% TGI (*P* = 0.0186), respectively. However, in B56α^–/–^ tumors, neither dose inhibited growth, indicating that the antitumor effect of RPT04402 is B56α-dependent (Supplemental Figure 14, A, B, and E). Mouse body weights remained stable, with no signs of toxicity (Supplemental Figure 14, C and D). Further dose-efficacy studies were conducted in *KRAS*-mutant NSCLC CDX models, including NCI-H441 and NCI-H640. In the NCI-H441 model, 10 mg/kg and 30 mg/kg RPT04402 (oral gavage; QD) resulted in 50.5% TGI (*P* = 0.0011) and 42.4% TGI (*P* = 0.0034), respectively, with median survival of 79 days for the 10 mg/kg group and 67 days for the 30 mg/kg group (Supplemental Figure 15, F–H). In the NCI-H460 model, 10 mg/kg RPT04402 (oral gavage; QD) produced a TGI of 40.7% (*P* = 0.0053 1) (Supplemental Figure 15, K and L). Importantly, no toxicities were observed in these models (Supplemental Figure 15, J and N). Overall, daily doses of 10–30 mg/kg RPT04402 effectively inhibited tumor growth without affecting animals’ health. Furthermore, RPT04402 did not exhibit a linear dose-response relationship in our A549 and NCI-H441 models given that increasing the dose beyond 10 mg/kg did not enhance efficacy, suggesting a potential saturation point.

### RPT04402 synergizes with MEK1/2 inhibition in NSCLC CDX.

**Figure 6:**
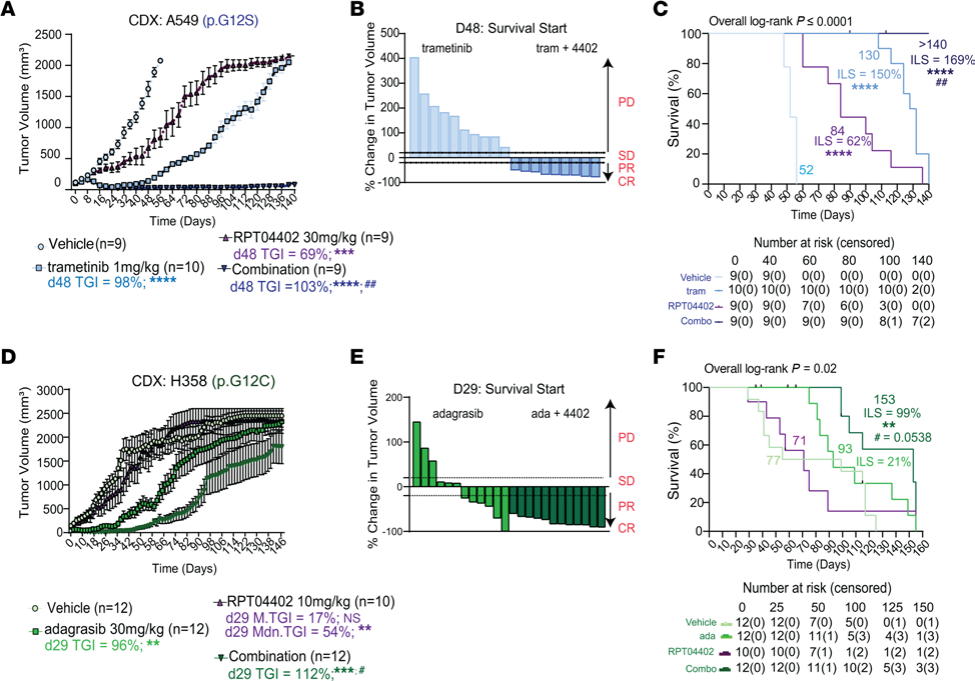
RPT04402 delays RAS/MAPKi-induced acquired resistance in *KRAS*-mutant NSCLC in vivo. (**A**) Tumor growth curves of A549 CDX mice treated with vehicle (*n* = 9), trametinib (1 mg/kg QD, *n* = 10), RPT04402 (30 mg/kg QD, *n* = 8), or the combination (*n* = 7). TGI was assessed at day 48; 2-way ANOVA with Tukey’s post hoc analysis was used for statistical significance. Statistical comparisons for vehicle versus all treatments: ***P ≤ 0.001, ****P ≤ 0.0001; trametinib versus combination: ^##^*P* ≤ 0.01. (**B**) Waterfall plot displaying the percentage change in tumor volume at day 48. RECIST criteria were used to categorize responses (PD, progressive disease; SD, stable disease; PR, partial response; CR, complete response). (**C**) Kaplan-Meier survival curves for A549 CDX. Median survival and increase in lifespan (increased lifespan) are shown; significance was determined using the log-rank (Mantel-Cox) test comparisons: vehicle versus all treatments: ****P ≤ 0.0001; trametinib versus combination: ^##^*P* ≤ 0.01. (**D**) Tumor growth curve for NCI-H358 CDX mice treated with vehicle (*n* = 8), adagrasib (30 mg/kg QD, *n* = 9), RPT04402 (10 mg/kg QD, *n* = 8), and the combination (*n* = 9). TGI was assessed at day 29. Statistical comparisons for vehicle versus all treatments: **P ≤ 0.01, ***P ≤ 0.0001; adagrasib versus combination, ^#^*P* ≤ 0.05. (**E**) Waterfall plot displaying the percentage change in tumor volume on day 29, categorized by RECIST. (**F**) Kaplan-Meier curves for NCI-H358 CDX with median survival and increased lifespan indicated. Log-rank test comparisons: vehicle versus all treatments, **P ≤ 0.01; adagrasib versus combination, ^#^*P* = 0.053.

Although MEK1/2 inhibitors exhibit strong antitumor activity in mouse models, single-agent suppression of MEK1/2 typically results in transient TGI, which is soon followed by tumor regrowth. This regrowth is often associated with the reactivation of MAPK signaling or the activation of compensatory pathways. In our cell-based experiments, we found that trametinib synergized with RPT04402 to enhance cytotoxicity, trigger apoptosis, and inhibit cell proliferation. Based on these results, we sought to determine whether the combination of trametinib and RPT04402 could prevent acquired resistance in vivo, as indicated by changes in tumor regrowth. We investigated the combination of trametinib and RPT04402 in an A549 CDX (Supplemental Figure 16A). Both trametinib (1 mg/kg, QD) and RPT04402 (30 mg/kg, QD) led to significant TGI, with mean TGI of 97% and 69%, respectively (Figure 6A, Supplemental Figure 16D). On day 48, the first animal in the vehicle group reached the study endpoint tumor volume (~2,000 mm³), and all vehicle-treated mice were removed by day 56. In contrast, the trametinib and RPT04402 single-agent treatment groups achieved median survivals of 130 and 84 days and increased lifespans of 150% and 62%, respectively, relative to vehicle (Figure 6C). Notably, both single-agent treatment groups eventually developed resistance, whereas the combination group did not. On day 48, the combination group produced a TGI of 103% (estimation plot in Supplemental Figure 16B), which was maintained for the duration of the study (~140 days) with an increased lifespan of 169% (Figure 6C). Furthermore, a waterfall plot revealed that 100% of the animals in the combination group had a greater than 50% decrease in tumor burden, with all mice in the combination arm maintaining tumor volumes below 100 mm³ (Supplemental Figure 16, E and F). In contrast, 100% of the animals in the trametinib group exhibited progressive disease (Figure 6B). These data suggest that RPT04402 can prevent trametinib-induced acquired resistance and that the combination promotes a durable antitumor response in vivo. Importantly, the combination was well tolerated, with no changes in body weight or condition (Supplemental Figure 16C).

All mice in this study were treated continuously after developing resistance, based on findings from an A549 CDX pilot study where doses were assigned prior to the conclusion of our pharmacokinetic studies. In our initial experiments, we tested 100 mg/kg and 300 mg/kg RPT04402, dosed every other day for 34 days and observed statistically significant TGI of 70% (*P* ≤ 0.001) and 80% (*P* ≤ 0.0001), respectively (Supplemental Figure 17, A and B). We combined the most efficacious dose of 300 mg/kg RPT04402 with 3 mg/kg trametinib and treated mice every other day. This dose of trametinib was chosen based on published dosing regimens of in vivo trametinib efficacy studies (31). As seen in our main study, the combination of trametinib and RPT04402 prevented tumor regrowth compared with either drug alone (Supplemental Figure 17F). However, when treatment ended on day 80, tumors in the combination group began to regrow (Supplemental Figure 17, E–H). Despite this regrowth, the combination still maintained a TGI of 103% for about 40 days after treatment cessation.

### RPT04402 potentiates KRAS-G12C inhibition in NSCLC CDX.

Our cell-based studies demonstrated that RPT04402 synergizes with adagrasib to enhance cytotoxicity, trigger apoptosis, and reduce cell proliferation. To assess the in vivo durability of this combination, we next evaluated adagrasib and RPT04402 in an NCI-H358 CDX model (Supplemental Figure 18A). As expected, adagrasib (30 mg/kg, QD) significantly inhibited tumor growth, achieving a mean TGI of 96%. In contrast, RPT04402 (10 mg/kg, QD) resulted in modest TGI, with a mean and median TGI of 17% and 54%, respectively (Figure 6D). On day 29, the first animal in the vehicle group reached the study endpoint tumor volume of 2,500 mm^3^, and the median survival of the vehicle group was 77 days (Figure 6F). One animal in the RPT04402 arm also reached the endpoint on day 29, and rapid tumor regrowth was observed in this group; median survival was 71 days (Figure 6F). Interestingly, animals in the RPT04402 arms at lower doses (1 or 5 mg/kg, QD) exhibited higher TGI on day 29 (56% and 42%, respectively) and median survival periods of 141 and 107 days, respectively (Supplemental Figure 15, A and C), underscoring a possible saturation point at 10 mg/kg in this model. Combined with our single-agent A549 and NCI-H441 CDX studies, this finding illustrates the context-dependent nature of RPT04402 response.

Although RPT04402 (10 mg/kg, QD) alone did not achieve significant TGI in the H358 CDX, the combination of adagrasib and RPT04402 enhanced TGI compared with adagrasib alone; the combination produced a TGI of 112% compared with a TGI of 96% by single-agent adagrasib (Figure 6D and Supplemental Figure 18 B, D–F). By day 29, 50% (6/12) of the adagrasib-treated animals had stable or progressive disease, whereas 100% (12/12) of the combination-treated animals displayed reductions in tumor burden (70%–95%) (Figure 6E). The combination group also reached a median survival of 153 days (increased lifespan = 99%), compared with 93 days (increased lifespan = 21%) for the adagrasib-only group. The difference between the adagrasib and combination curves was not statistically significant (*P* = 0.053), though it approached the conventional threshold. As observed in the A549 CDX study, the combination was well tolerated, with no significant changes in body weight or condition (Supplemental Figure 18C). These findings suggest that although RPT04402 potentiates the antitumor effects of adagrasib and delays the onset of acquired resistance, it does not completely prevent resistance development, which could potentially be achieved using a different dose of RPT04402 in this model.

### RPT04402 potentiates trametinib-induced apoptosis and antiproliferative activity in vivo.

A pharmacodynamic study was conducted to evaluate the effects of RAS/MAPK inhibitors, RPT04402, and their combination on key RAS/MAPK signaling effectors, including pERK, pAKT, me-PP2ACα, and methylation modulators. This study was necessary because our long-term combination studies induced significant tumor regression, making it difficult to collect sufficient tumor tissue for molecular analyses.

**Figure 7:**
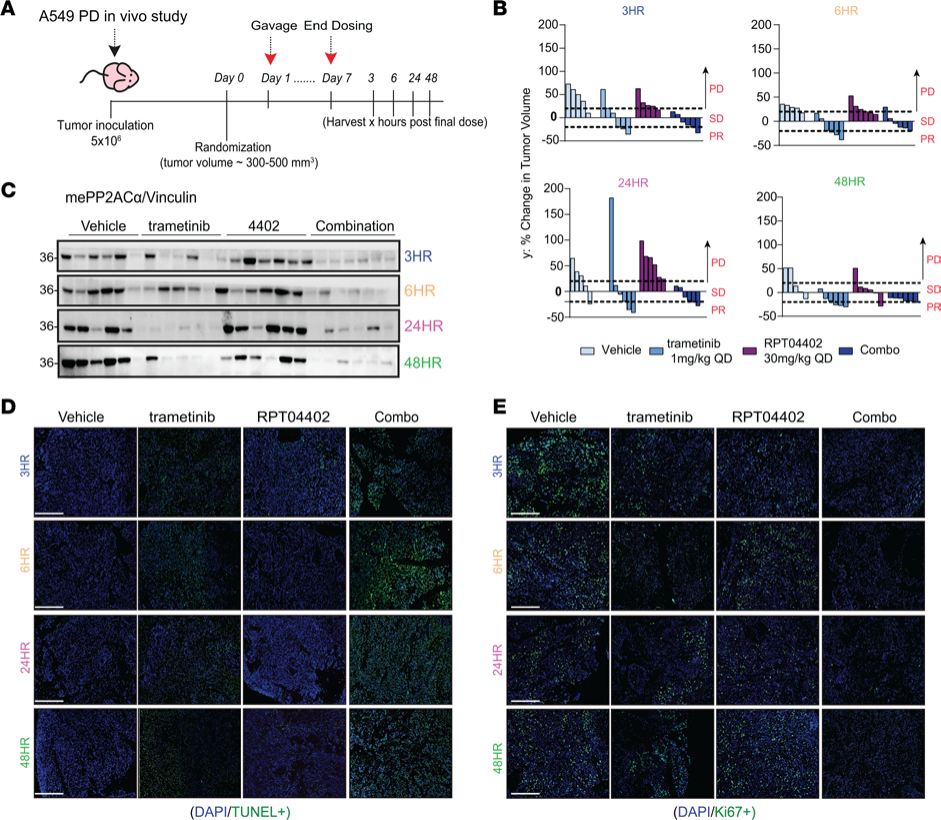
RPT04402 enhances trametinib-induced apoptosis in vivo. (**A**) Schematic of the A549 pharmacodynamic study. Mice were treated daily for 7 days with vehicle, trametinib (1 mg/kg), RPT04402 (30 mg/kg), or the combination. Mice were euthanized 3, 6, 24, or 48 hours after the final dose for analysis. (**B**) Waterfall plots displaying the percentage change in tumor volume for each treatment arm 3, 6, 24, and 48 hours after the final dose. The plot indicates responses based on RECIST criteria: PD, progressive disease; SD, stable disease; PR, partial response; CR, complete response. (**C**) Western blot analysis of mePP2ACα in tumors from A549 CDX. Blot quantifications are located in Supplemental Figure 21. (**D**) Representative images of TUNEL staining (green) from histological sections of A549 CDX tumors harvested 3, 6, 24, and 48 hours after the final dose. Between 5 and 15 images per tumor slice were captured to calculate the average percentage of TUNEL-positive cells. (**E**) Representative images of Ki67 staining (green) from A549 CDX pharmacodynamics as described in **D**. Nuclei are stained with DAPI. Scale bar: 75 μm. Graphs and quantifications are in Supplemental Figure 21. Raw values are in the Supporting Data Values file.

The investigation focused on the A549 CDX model and the trametinib-RPT04402 combination, which demonstrated the most pronounced effect in delaying tumor regrowth. Briefly, 5 × 10^6^ A549 cells were injected subcutaneously into male nude mice. After tumors reached a size of 300–500 mm^3^, mice were randomized into 4 groups: vehicle, 1 mg/kg trametinib, 30 mg/kg RPT04402, and the combination. Treatments were administered orally once daily for 7 days (Figure 7A), based on our initial combination study where tumor regression was observed by day 7 (Figure 6A). For downstream analyses, mice were euthanized at 3, 6, 24, or 48 hours after the final dose. By the end of the treatment period, there were no significant differences in tumor volume or weight across groups (Figure 7B and Supplemental Figure 19, A and B).

Western blot analysis of A549 tumors revealed that trametinib monotherapy effectively suppressed pERK expression, with the most pronounced effect observed 3 and 6 hours after the final dose (Supplemental Figure 20, A and B). In contrast, RPT04402 alone had no effect on pERK expression at any time compared with the vehicle control. The combination treatment mirrored the effect of trametinib monotherapy, indicating that the suppression of pERK was driven entirely by trametinib. Additionally, pERK reactivation was observed in both single-agent and combination groups at 24 and 48 hours after treatment. Interestingly, total ERK levels were significantly reduced in the combination group at 3 hours after treatment, a finding not observed with either monotherapy. However, this effect was transient, and although a statistically significant reduction in total ERK levels was noted at 48 hours in the combination group, it was driven by a single outlier in the vehicle control group (Supporting Data Values file).

Next, we examined p-AKT (pT308 and pS473) and total AKT expression. Trametinib monotherapy did not induce hyperactivation of p-AKT at either phosphosite. Instead, it reduced p-AKT levels at both pT308 and pS473, with the reduction at 48 hours being statistically significant (Supplemental Figure 20, A–D). RPT04402 alone had no consistent impact on p-AKT levels except for an isolated increase in pT308 at 24 hours, which was driven by a single tumor. In the combination group, p-AKT (pT308) was elevated at 3 hours after treatment, but this effect was not sustained at later time points. Like trametinib monotherapy, the combination group showed reductions in both pT308 and pS473 levels at 48 hours, with a greater effect on pS473 compared with trametinib alone (Supplemental Figure 20D). Consistent with other in vivo studies, trametinib was the primary driver of reductions in total AKT expression in both the single-agent and combination groups (Supporting Data Values file). In contrast, RPT04402 had virtually no effect on total AKT levels compared with the vehicle control, except for a transient increase at the 24-hour time point, which was not observed at any other time point or treatment condition (Supporting Data Values file).

IHC-based TUNEL staining confirmed that trametinib induced apoptosis in vivo (Figure 7D). The percentage of TUNEL-positive cells in the trametinib group was 7-fold higher than the control (36.1% vs. 5.5%), with this difference maintained across all time points (3, 6, 24, and 48 hours). The addition of RPT04402 to trametinib significantly enhanced apoptosis, with nearly 50% more TUNEL-positive cells compared with trametinib alone (Figure 7D and Supplemental Figure 21B). In contrast, single-agent RPT04402 did not induce apoptosis (Figure 7D), aligning with the tumor volume changes (Figure 7B), where only the trametinib and combination groups showed tumor regressions. This suggests that the tumor regressions observed in our A549 CDXs were primarily driven by trametinib, while RPT04402 resulted in tumor stasis or a slower growth rate (Figure 7B and Supplemental Figures 15 and 17). However, RPT04402 appeared to enhance the apoptotic/cell death effects of trametinib.

We next evaluated the effects of each treatment on proliferation using Ki67 staining. As expected, vehicle-treated tumors had the highest proliferative index, and both trametinib and the combination arms consistently reduced Ki67 staining (Figure 7E and Supplemental Figure 21C). RPT04402 also reduced Ki67 levels but only 3 hours after the final dose. Like the TUNEL staining, the primary inhibitory effects on proliferation seemed to be driven by trametinib. At early time points (3 and 6 hours), the combination of trametinib and RPT04402 enhanced proliferation inhibition compared with trametinib alone (3 hours: *P* = 0.02; 6 hours: *P* = 0.03), though this effect was diminished by 24 and 48 hours. At the later time points, single-agent trametinib consistently reduced proliferation relative to vehicle; however, the combination produced and maintained a 10-fold or greater reduction in Ki67-positive cells that was significantly greater than that observed for trametinib alone (vehicle vs. trametinib and vehicle vs. combination) (Supplemental Figure 21C). These findings align with the TUNEL staining, where increased cell death in the combination (relative to trametinib) coincided with the largest reduction in proliferation. Similarly, RPT04402 alone did not reduce Ki67 staining, mirroring its limited impact on cell death or proliferation. Overall, combining RPT04402 with trametinib bolstered both the proapoptotic and antiproliferative effects of trametinib.

Finally, we examined the expression of mePP2ACα, PP2ACα, B56α, PME1, and LCMT1. Trametinib treatment reduced mePP2ACα expression in both the single-agent and combination arms (Figure 7C and Supplemental Figure 21A). Although statistical significance was only achieved at the 48-hour time point, this variability likely reflects small sample sizes and the limitations of significance testing, given that the Western blots consistently showed a clear reduction compared with the vehicle control. RPT04402 alone increased mePP2ACα expression at the 24-hour time point; however, its addition to trametinib in the combination groups did not rescue mePP2ACα expression. Total PP2ACα levels remained largely unchanged except at the 24-hour and 48-hour time points. At 24 hours, trametinib treatment increased PP2ACα expression, whereas at 48 hours, the combination group showed reduced expression compared with the vehicle control (Supplemental Figure 20, A–D). For PME1 and LCMT1 expression, trametinib monotherapy consistently reduced their levels. The addition of RPT04402 did not restore PME1 or LCMT1 expression; however, at 24 hours, Western blots showed a retention of LCMT1 expression in the combination arm compared with trametinib alone, though this was not statistically significant (*P* = 0.0819) (Supplemental Figure 19, C–F).

Trametinib also decreased B56α expression at all time points, with peaks at 24 hours (*P* < 0.001) and 48 hours (*P* = 0.0242). In contrast, single-agent RPT04402 enhanced B56α expression at 3 hours (*P* = 0.0101), 6 hours (*P* = 0.0692), and 24 hours (*P* = 0.0057) after treatment compared with the vehicle control. However, this enhancement was abolished by 48 hours (Supplemental Figures 19, C–F). Interestingly, at 6 hours after treatment, the combination group began to show B56α expression retention compared with trametinib monotherapy (*P* = 0.0638). By 24 hours, the combination group displayed a statistically significant retention of B56α expression compared with trametinib alone (*P* = 0.0255) (Supplemental Figure 19E). This rescue of B56α may have contributed to the enhanced cell death and antiproliferative effects observed in the combination arm, which peaked at 24 hours in this study (Figure 7, D and E).

Our pharmacodynamic analyses suggest that the synergy between trametinib and RPT04402 is driven by enhanced cell death, apoptosis, and antiproliferative effects compared with trametinib monotherapy. The combination did not permanently prevent the reactivation of pERK or pAKT but did lead to a reduction in total ERK and total AKT levels, particularly in the combination groups. This reduction likely reflects a compensatory response by tumor cells to counteract cell death induced by other effectors modulated by the combination.

## Discussion

The development of combination therapies to prevent acquired resistance to single-agent RAS/MAPK inhibitors is crucial for improving treatment outcomes in metastatic *KRAS*-mutant NSCLC. Unfortunately, most current strategies focus heavily on GTPase/kinase or kinase-kinase interactions, often overlooking the vital role phosphatases play in RAS/MAPK signaling and therapeutic response. Acquired resistance to MEK1/2 or KRASG12C inhibitors is frequently tied to phosphatase inhibition, further underscoring their potential. However, the extent to which cells dysregulate PP2A activity to drive resistance is currently unclear, demanding further studies.

In our current study, we found that RAS/MAPKi reduces the carboxymethylation of PP2ACα and destabilizes tumor-suppressive PP2A-B56α heterotrimers. These heterotrimers are key negative regulators of RAS/MAPK effectors, including pERK and pAKT, which drive bypass mechanisms of resistance. Although direct clinical evidence of PP2A inactivation following resistance to RAS/MAPK has not been reported, our data suggest that suppression of PP2A activity may contribute to adaptive signaling and therapeutic relapse. Future studies analyzing PP2A carboxymethylation or holoenzyme composition in posttreatment clinical biopsies will be critical to validating this mechanism in patients.

Building on our previous work demonstrating that the PP2A molecular glue DT-061 enhances the efficacy of the MEK1/2 inhibitor selumetinib, we now report that the molecular glue RPT04402 enhances therapeutic outcomes when combined with trametinib (MEK1/2i) or adagrasib (KRASG12Ci). This combination not only drives tumor regression in vivo but also delays the onset of acquired resistance, extending treatment efficacy to over 100 days.

RPT04402 operates via a distinct mechanism compared with DT-061. Whereas DT-061 delays the off-rate of B56α (29), RPT04402 enhances the on-rate of PP2A-B56α heterotrimerization, enabling activity even in conditions where PP2ACα methylation is compromised. This makes RPT04402 uniquely suited to address resistance mechanisms induced by RAS/MAPKi, underscored by surface plasmon resonance experiments (Derek J. Taylor, Case Western Reserve University, unpublished observations). Specifically, RPT04402 enhances the affinity of B56α for the A-C dimer only when PP2ACα is demethylated—either partially (as seen in a 1:1 mix of unmethylated and methylated PP2ACα) or entirely (via C-terminal truncation). This property likely explains why trametinib- or adagrasib-induced demethylation of PP2ACα creates an environment for RPT04402 to restore PP2A-B56α activity both in vitro and in vivo.

Our cell-based studies highlight the dependence of RPT04402’s activity on B56α, with B56α^–/–^ cells displaying resistance to RPT04402 treatment compared with WT controls. Although CTG assays did not fully reflect in vivo responses, clonogenic assays, which measure long-term survival, provided stronger alignment. These findings were further corroborated in vivo, where we observed that, in our efficacy studies, RPT04402 slowed tumor growth or promoted tumor stasis, which was completely abrogated with B56α^–/–^, underscoring the functional importance of B56α in mediating the antitumor effects of RPT04402.

Although the combination of RPT04402 with trametinib or adagrasib enhanced apoptotic and antiproliferative effects, the exact targets or substrates mediating this synergy remain elusive. pERK and pAKT were selected as markers of acquired resistance based on their established roles in bypass signaling after RAS/MAPKi. However, neither protein’s hyperactivation — observed after trametinib or adagrasib monotherapy — was significantly suppressed by the addition of RPT04402. Instead, the combination appeared to maintain or even promote pERK and pAKT hyperactivation, suggesting an alternative mechanism of action. One possibility is that the synergy stems from downstream consequences like reduced global protein synthesis during apoptosis, a hallmark of cellular stress. Supporting this concept, we observed that trametinib reduced total AKT levels in vivo, whereas RPT04402 alone had no effect on phosphorylated or total protein levels. The combination uniquely reduced total ERK levels early during treatment — an effect neither single agent achieved independently.

B56α, while not directly regulating ERK translation or degradation, modulates ERK activity via broader signaling effects. These include interactions with RAF kinases, direct effects on ERK activation sites (Thr202/Tyr204), or modulation of mTOR signaling and downstream effectors like 4E-BP1 and S6K (10, 32, 36). Future studies will examine whether ERK reactivation and AKT hyperphosphorylation after RAS/MAPKi drive resistance or if these compensatory responses are unrelated to the combination’s mechanism. Phosphoproteomic analysis may reveal which pathways are most affected and contribute to synergy with trametinib and whether this synergy is generalizable. Notably, chemotherapies also upregulate PP2A-B56α–regulated pathways like Wnt/β-catenin and MAPK to combat chemo-induced cell death (37–39). Thus, combining RPT04402 with a chemotherapeutic could yield synergy like that seen with RAS/MAPKi to bypass chemoresistance. Interestingly, overexpression of B56α has been shown to combat the Wnt-mediated cell survival and cisplatin-resistance driven by miR-218 in oral cancer (40).

PP2A-B56α heterotrimers are also implicated in apoptotic pathways (24, 36). In our study, the combination of RPT04402 and trametinib rescued B56α protein levels, coinciding with peak apoptosis and reduced Ki67 staining in our pharmacodynamic analyses. PP2A-B56α heterotrimers also play a key role in cell cycle regulation by controlling G1/S and G2/M transitions through dephosphorylation of critical targets like cyclin-dependent kinases (41–43). Loss of downregulation of B56α can disrupt checkpoint control, leading to increased proliferation. In our context, stabilizing trametinib-induced loss of PP2A-B56α complexes in vitro and in vivo with RPT04402 may help restore proper cell cycle regulation and suppress aberrant proliferation and promote apoptosis. PP2A-B56α heterotrimers can also affect the stability of p53 by promoting its degradation through dephosphorylation of HDM2, a negative regulator of p53. PP2A-B56α activity on p53 eventual leads to p53-mediated apoptosis (44, 45).

In general, there are many pathways outside of ERK and AKT that PP2A-B56α heterotrimers regulate that could synergize with RAS/MAPKi. A limitation of our work is the inability to definitively characterize the specific populations of PP2A-B56 heterotrimers present and active after treatment. Our initial co-IP experiments revealed that trametinib and adagrasib destabilize tumor-suppressive PP2A-B56α heterotrimers. However, we cannot directly measure the activity of a specific heterotrimer due to limitations in commercially available activity assays. Additionally, the extensive cell death induced by combination treatments complicates the completion and accurate assessment of co-IP products because surviving cells likely represent resistant subsets. For example, we observed upregulation of PR130 and its enhanced binding to V5-tagged PP2A-Aα after trametinib treatment at the latest time point; in certain cases, PR130 is classified as tumor-promoting due to its modulation of PI3K/AKT and WNT signaling cascades (46). Future studies will incorporate greater temporal analyses coupled with phosphoproteomics to map the signaling networks influenced by RPT04402 and RAS/MAPK inhibitors. Additionally, modifications to proximity ligation assays to include real-time live cell imaging could be developed to monitor PP2A-B56 family interactions.

Our xenograft studies revealed a stronger synergy between RPT04402 and trametinib compared with adagrasib. Although both MEK1/2 and KRASG12C inhibitors activate similar compensatory pathways and disrupt PP2A function, their resistance mechanisms differ. Resistance to MEK1/2 inhibitors typically involves bypassing ERK inhibition via alternative MAPK routes, whereas KRASG12C inhibitor resistance often arises through mutations in K-Ras or compensatory pathway activation. Notably, PP2A-B56α is a known direct regulator of RAF and ERK kinases; therefore, the effects of RPT04402 are more aligned with modulating MAPK pathway components rather than directly targeting K-Ras. Although we did not test the adagrasib and RPT04402 combination in a pharmacodynamic study, resistance to G12Ci has also been shown to include the resynthesis of drug-resistant K-Ras protein, which is dependent on EGFR and AURKA signaling (8). We have previously found that PP2A inactivation creates dependencies for EGFR and AURKA inhibitors (23). Additionally, PP2A heterotrimers are known to negatively regulate both pathways (47–49); therefore, RPT04402 could mitigate this early adaptive resistance through these pathways by augmenting PP2A-B56α activity on EGFR and AURKA effectors.

Finally, we observed that RAS/MAPK inhibitors inhibit PP2ACα carboxymethylation without affecting the protein levels of methylation regulators LCMT1 or PME1 in vitro. However, our in vivo pharmacodynamic study revealed that trametinib decreases not only mePP2ACα but also LCMT1 and PME1 levels, highlighting discrepancies likely due to differences in treatment conditions — sustained treatment in vivo may better reveal the methylation dynamics underlying resistance.

Importantly, LCMT1-KO cells exhibited altered responses to MEKi both in vitro and in vivo, suggesting that LCMT1 loss may contribute to therapeutic resistance. To further investigate this, ongoing studies are evaluating whether LCMT1, PME1, or both mediate the reduction in mePP2ACα observed after RAS/MAPK pathway inhibition in vivo. To delineate the underlying mechanism, we are assessing how PME1 KO affects treatment response and methylation dynamics, as a complement to the LCMT1 studies. Additionally, we are exploring how enzymatically inactive LCMT1 and PME1 mutants influence mePP2ACα after RAS/MAPKi. If LCMT1 and PME1 protein levels remain stable after RAS/MAPKi treatment in vitro or with a shorter treatment period, then changes in enzymatic activity, and not expression, may drive mePP2ACα loss. Alternatively, the C-subunit may not interact with LCMT1 or PME1 after RAS/MAPKi, thereby affecting mePP2ACa levels. We are optimizing proximity ligation assays between the C-subunit and LCMT1 and/or PME1. Collectively, these ongoing studies will provide better understanding of resistance mechanisms after RAS/MAPK pathway inhibition.

In conclusion, our findings establish RPT04402 as a molecular glue that reactivates tumor-suppressive PP2A-B56α heterotrimers deconstructed by RAS/MAPK inhibitors. Unlike other agents, RPT04402 promotes heterotrimer formation, even under conditions of impaired PP2ACα methylation. Its ability to synergize with trametinib and adagrasib, delay resistance, and extend treatment responses represents a step forward in overcoming therapeutic barriers in treating metastatic *KRAS*-mutant NSCLC. By restoring PP2A activity and influencing critical apoptotic pathways, RPT04402 opens new avenues for combination treatments targeting phosphatase-mediated mechanisms in cancer therapy. Recently, K-Ras degraders have offered a promising approach by eliminating the protein, but they remain clinically unproven and may face limited efficacy due to resistance mechanisms like target mutations, E3 ligase alterations, or pathway rewiring that reduces K-Ras dependency (50–53). Thus, combining a K-Ras degrader with RPT04402 (or another PP2A molecular glue) could help prevent resistance to this mechanism or another to some extent. Overall, our results set the stage for future investigations into the molecular mechanisms driving RPT04402 activity and its broader applicability across RAS/MAPK-driven cancers and current treatments.

## Methods

### Sex as a biological variable.

Sex was not considered a factor in any of the study designs; however, all in vivo studies utilized male mice. Although lung cancer occurs in both males and females, there is no evidence of sex differences in responses to the drugs used in this study.

### Statistics.

Statistical analyses were performed using GraphPad Prism (version 10) or RStudio (version 2024.12.1). All statistical tests were 2-sided with significance set at *P* value of 0.05 or less. Continuous variables were compared using an unpaired *t* test, 1-way ANOVA, or 2-way ANOVA assuming normal distribution and equal variances. The choice of test is specified in the figure legends. For multiple comparisons, α level was adjusted using Tukey’s honestly significant difference test as we compared all treatment groups. When post hoc comparisons were considered, Tukey’s, Dunnett’s, or Šidák’s multiple-comparison tests were utilized. When data exhibited unequal variances, a mixed-effect analysis was used. In vitro data are based on 3 or more independent biological replicates. In vivo data analyses are described in the corresponding figure legends.

### Study approval.

All procedures related to animal handling, care, and treatment were performed under an approved protocol (PRO00010410) according to the University of Michigan IACUC and following the guidance of the Association for Assessment and Accreditation of Laboratory Animal Care.

### Data availability.

All relevant data for this study are included in the article and supplemental materials. Values for all data points in synergy plots, graphs, and densitometry of Western blots are reported in the Supporting Data Values file. Detailed descriptions of the methods and the key resources table are in a supplemental file titled Supplemental Methods.

## Author contributions

BR, STR, and GN conceptualized the study. BR, STR, IP, KPZ, KB, ACD, AMD, TKL, SM, AA, BKM, and JCB developed methodology. BR, STR, ACD, AMD, MH, KPZ, KB, GHO, SM, TKL, DJT, and AA conducted the investigation. Data visualization was performed by BR, STR, and ACD. Resources were provided by BR, STR, AD, KPZ, GHO, MH, DJT, BKM, and JCB. GN acquired funding for the study. Project administration was carried out by BR, STR, ACD, and GN, and GN supervised the study. The original draft was written by BR, STR, ACD, and GN, with BR, STR, ACD, GN, and CMO contributing to the review and editing of the manuscript.

## Funding support

This work is the result of NIH funding, in whole or in part, and is subject to the NIH Public Access Policy. Through acceptance of this federal funding, the NIH has been given a right to make the work publicly available in PubMed Central.

- Department of Defense HT9425-23-LCRP-TRA (to GN).
- NIH CMB training grant T32 GM145470 (to BR).
- RAPPTA Therapeutics (to GN).
- National Cancer Institute of NIH P30 CA046592 (to Flow Cytometry Core, University of Michigan Biomedical Research Core Facilities ([BRCF]).

## Supplementary Material

Supplemental data

Unedited blot and gel images

Supporting data values

## Version 1 10/14/2025

In-Press Preview

## Version 2 12/01/2025

Electronic publication
