# Supplementary material for: A PP2A molecular glue overcomes RAS/MAPK inhibitor resistance in KRAS-mutant non–small cell lung cancer
Source: J Clin Invest. 2025 Oct 14;135(23):e193790. doi: 10.1172/JCI193790 (PMC12646674; doi:10.1172/JCI193790)
Supplement: Supplemental data [file jci-135-193790-s077.pdf]

Supplemental Information

A PP2A Molecular Glue Overcomes RAS/MAPK Inhibitor Resistance in *KRAS*-mutant Non-Small Cell Lung Cancer

Brynne Raines[1,2,4], Stephanie S. Tseng-Rogenski[3], Amanda C. Dowdican[2,4], Irene Peris[2,4,] Matthew Hinderman[2,4], Kaitlin P. Zawacki[2,4], Kelsey Barrie[2,4], Gabrielle Hodges Onishi[2,8], Alexander M. Dymond[2,4,9], Tahra K. Luther[2,4,10], Sydney Musser[2,,11], Behirda Karaj Majchrowski [5,6], J. Chad Brenner[1,4,5,6], Aqila Ahmed[2,4,12], Derek Taylor[7,8], Caitlin M. O'Connor[2,4], Goutham Narla[1,2,4]*.

Correspondence should be addressed to:

*Goutham Narla:
Rogel Cancer Center, Room C3215
1500 E. Medical Center Drive
Ann Arbor, MI 48109, USA
Phone: +1-734-615-2411
Email: gnarla@umich.edu

Affiliations:

[1]Cellular and Molecular Biology Program, University of Michigan, Ann Arbor, Michigan, USA
[2]Department of Internal Medicine: Division of Genetic Medicine, University of Michigan, Ann Arbor, Michigan, USA
[3]Department of Internal Medicine: Human Genetics, University of Michigan, Ann Arbor, Michigan, USA
[4]Rogel Cancer Center, University of Michigan Health, Ann Arbor, Michigan, USA
[5]Department of Pharmacology, University of Michigan, Ann Arbor, Michigan, USA
[6]Department of Otolaryngology, University of Michigan, Ann Arbor, Michigan, USA
[7]Department of Biochemistry, Case Western Reserve University, Cleveland, Ohio, USA
[8]Department of Pharmacology, Case Western Reserve University, Cleveland, Ohio, USA

Current Affiliations:

[8]Department of Internal Medicine: Pulmonary/Critical Care, University of Michigan, Ann Arbor, Michigan, USA
[9]David Geffen School of Medicine, University of California – Los Angeles, Los Angeles, California, USA
[10]Department of Internal Medicine: Division of Cardiovascular Medicine, University of Michigan, Ann Arbor, Michigan, USA
[11]Department of Pathology: Molecular & Cellular Pathology Program, University of Michigan, Ann Arbor, Michigan, USA
[12]Market Development Group, Bio-Rad Laboratories, Ann Arbor, Michigan, USA

Conflict of Interest Statement: G.H.O, K.P.Z, and K.B received personal fees from RAPPTA Therapeutics during the completion of this work. DJT and CMO report receiving consulting fees from RAPPTA Therapeutics. G.N. receives research support from RAPPTA Therapeutics and has an equity interest and receives consulting fees from RAPPTA Therapeutics. No disclosures were reported from other authors.

Supplemental Methods

Cell Lines and Cell Culture

Human non-small cell lung cancer cell lines (A549, H358, H23, H1373, H2030, H2444) were purchased from the American Type Culture Collection (ATCC). The patient-derived cell line, J000096652, was established using a passaged tumor excised from a female NSG (NOD.Cg-Prkdc^{scid}Il2rg^{tm1Wjl}/SzJ) patient-derived xenograft purchased from Jackson Laboratories. Briefly, fresh tumor fragments were placed into tissue-cultured treated plates in Roswell Park Memorial Institute 1640 (RPMI 1640) (cat no: 11875119; Gibco) supplemented with 10% fetal bovine serum (FBS) (cat no: 97068-085; VWR) and 0.5% penicillin/streptomycin (pen-strep) (cat no:15140122 ; Gibco). After one week, the tissue fragments were removed, and the remaining adhered cells were allowed to propagate. The cells underwent repeated passaging to isolate the tumorigenic cells. All cell lines were maintained in RPMI 1640 supplemented with 10% FBS and 0.5% pen-strep and stored in a humidified incubator at 37°C with 5% CO_2 and underwent standard passaging. All cell lines underwent monthly testing for mycoplasma contamination using the MycoAlert® Mycoplasma Detection Kit (LT07-318; Lonza) and were authenticated by short tandem repeat (STR) analysis (ATCC, human and mouse STR profiling service).

Constructs and Lentiviral Production

PP2A V5-tagged Aα: pLX304 PPP2R1A plasmid (HsCD00444402) was purchased from DNASU (Arizona State University Plasmid Repository).

V5-tagged LCMT1: pLX304 LCMT1 plasmid (HsCD00441432) was purchased from DNASU (Arizona State University Plasmid Repository). For reconstitution studies, site-directed mutagenesis of the PAM site was performed using the Agilent QuikChange Lightning kit (cat no: 210515). Mutations to the PAM site can be viewed in the **Key Resources Table.**

LCMT1 and PPP2R5A knockouts: Guide RNAs (gRNAs) to *LCMT1* (LCMT-1) and *PPP2R5A* (B56α) were designed using ATUM Bio's CRISPR design tool. Custom plasmids were obtained from GenScript. Genscript licenses CRISPR technology derived from Feng Zhang's laboratory at the Broad Institute. gRNAs were cloned into the vector pLentiV2 using the CloneEZ method developed by GenScript. Two gRNAs were chosen for each gene and can be viewed in the **Key Resources Table.** Plasmids were verified by Sanger sequencing as part of Genscript's Quality Control procedures and by the Narla Lab through Molecular Cloning Laboratories (MCLAB).

All lentiviruses were generated in collaboration with the Vector Core at the University of Michigan. For lentiviral transduction of A549 and H358 cells, roughly 100,000 cells of each line were seeded in a 6-well plate (cat no: 229106; CELLTREAT) with complete media (RPMI + 10%+ 0.5% pen-strep). Cells were incubated with 300μL of 10X virus (1.5mL total volume) at a 3X final concentration for 48 hours. After transduction of V5-tagged Aα or V5-tagged LCMT1, cells were selected in 16 μg/mL Blasticidin (Invivogen, cat no: ant-bl-5b) for 72 hours to generate stable cell lines. Post selection, cells were maintained in 8 μg/mL Blasticidin in complete media. For *LCMT1* and *PPP2R5A* knockout studies, cells were selected with 3 μg/mL of Puromycin (Invivogen, ant-pr-1) for 72 hours and then maintained in 1 μg/mL of Puromycin in complete media. Cells were maintained at a subcultivation ratio of 1:2. For overexpression studies, lysates were collected during each passage and subjected to western blotting for protein expression of Aα or LCMT1. Knockout lysates were collected and analyzed for protein expression of LCMT1 or B56α. Three biological replicates were used to confirm overexpression or knockout. For knockout studies, monoclonal A549 (*LCMT1* and *PPP2R5A* knockout) and H358 (*PPP2R5A* knockout) subclones were generated in collaboration with the Flow Cytometry Core at the University of Michigan using Sony Biotechnology's SH800 cell-sorter.

Compounds

Trametinib (cat no: S2673), selumetinib (cat no: S1008), adagrasib (MRTX849) (cat no: S8884), and sotorasib (AMG510) (cat no: S8830) were obtained from Selleckchem. RPT04402 was synthesized by Evotec and retained in the Narla Lab at the University of Michigan for use. In powder form, RPT04402 was stored at -80°C. For all in vitro studies, trametinib, selumetinib, adagrasib, and sotorasib were dissolved in 100% dimethyl sulfoxide (DMSO) as a 10 mM stock solution, aliquoted, and stored at -80°C until use. RPT04402 was dissolved in DMSO for an 80 mM stock solution and stored at -80°C until use.

For *in vivo* studies, appropriate weights of trametinib (cat no: S2673; Selleckchem), adagrasib (cat no: HY-130149; MedChem Express), and RPT04402 were dissolved to yield a 10% N, N-dimethylacetamide (DMA'), 10% Solutol ® HS_{15} (Kolliphor ® HS_{15}, 'Solutol') and 80% water solution. The procedure for reconstitution is as follows: DMA was added to each compound and vortexed for 1 minute for dissolution. Next, prewarmed Solutol ® HS_{15} (Kolliphor ® HS_{15}, 'Solutol') was added to each compound and vortexed for 1 minute. Lastly, water pre-warmed to

40-45°C was slowly added to the drug solutions up to the final calculated volume and vortexed for 1 minute. Prior to dosing animals, all drugs were removed from the bead bath and allowed to cool to room temperature. For combination studies, drugs were combined 10 minutes prior to dosing. Prepared drugs were used within 7 days of mixing.

Protein Extraction, Western Blotting, and Analysis

Cells in 10-cm or 15-cm plates were collected, and cell lysates were prepared by incubating cell pellets in Pierce 1x IP lysis buffer (cat no: 87787; Thermofisher) for 24 hours at -20°C. Lysis buffer was supplemented with protease (cat no: 05892791001; Sigma-Aldrich) and phosphatase inhibitors (cat no: 04906837001; Roche). After 24 hours, pellets were thawed, promptly sonicated (30% power for 45 seconds), and centrifuged at >20,000 x g for 15 minutes at 4°C to remove cell debris. Protein concentration was determined using the Pierce BCA Protein Assay (cat no: 23250; Thermofisher). Approximately 35 μg of protein was separated using TGX stain-free 4-15% polyacrylamide gels (cat no: 4568086; Bio-Rad), transferred to nitrocellulose membranes (cat no: 1704158; Bio-Rad) and blocked in 3-5% milk (cat no: M17200-1000; Research Products International) in 1x TBS-T for 1 hour. Primary antibodies were diluted in 5% BSA and blots were incubated with primary overnight at 4°C, washed, and then incubated for 1.5 hours with secondary antibody at room temperature. Details of specific primary and secondary antibodies are listed in **Key Resources Table**. Blots were imaged using the Bio-Rad ChemiDoc XRS+ and densitometric analyses were performed using Bio-Rad Image Lab software. The analysis of specific western blots is detailed in the figure legends, and densitometric analysis protocols for both *in vitro* and *in vivo* studies were followed as previously described (1).

Co-immunoprecipitation

Cell lysates were prepared following the standard protein extraction protocol. Lysates were then subjected to co-immunoprecipitation using the Dynabeads™ Co-Immunoprecipitation Kit (cat no: 14321D; Thermofisher). For each reaction, 7 μg of V5-Tag antibody (cat no: MCA1360G; Bio- Rad) was conjugated to the M-270 Epoxy beads for 16-24 hours at 37°C. The total volume of V5- tag antibody depended on the number of reactions (e.g., 1 rxn = 7μg). 1.5 mg (150 μL) of the conjugated mixture was used for each IP. Denatured pulled-down proteins were subjected to western blotting to identify interacting proteins. 10% of the whole cell lysate was used as 'input' controls.

Cell Viability and Proliferation Assays

Cell Titer Glo: Depending on the cell line, 1,500-3,000 cells were plated in Nunc™ MicroWell™ 96-well black-walled, clear-bottom plates (cat no: 165305; Thermo Scientific) in standard tissue culture conditions (2D) and in 96-well black and clear round bottom plates (cat no: 4520, Corning) for low-adherent (3D) conditions. All cells, in duplicates, were plated in 75 µL of complete media and incubated for 24 hours. Complete media (100 µL) was added to the outer wells of the 96-well plates to avoid evaporation of media and to act as a "blank" (no cells) for an additional control. Compounds were plated at ten different concentrations covering a 10,000-fold concentration range (.3 nM-10 µM). 75 µL of the drugs, at 2X of the intended concentration, was added on top of the seeded cells for a total volume of 150 µL. Cells were placed in a humidified incubator at 37°C with 5% CO_2 for 72 hours (2D and 3D). Cell Titer Glo 2.0 (cat no: G9242; Promega) and Cell Titer-Glo(R) 3D (cat no: G9683; Promega) were used to measure cell viability in 2D and 3D conditions, respectively. Plates were read using the BioTek Synergy HTX Multiplate Reader using the "Cell Luminescence" protocol. Data were analyzed by averaging the duplicate wells for each concentration, then subtracting the luminescence values of the blank wells from the treated wells. All values were then normalized to the DMSO treated wells. The half maximal inhibitory concentrations (IC50) values were determined using GraphPad Prism (GraphPad, San Diego, CA). In GraphPad Prism, drug concentrations were log transformed, and the effect of the compounds were averaged and plotted as % of vehicle control. Log(inhibitor) vs. response: variable slope (four parameters) was used, and top best-fit value was set to 100 to calculate IC50 values. Studies were completed ≥ 3 times.

Clonogenic Assays: Depending on the cell line, 120-500 cells per well were seeded into 24-well plates for a total volume of 1 mL. After 24 hours, drugs were added to each well. DMSO and inhibitor(s) in the media were replenished every 3-4 days for the number of days noted. Intervals to replenish media were determined based on published literature identifying the half-life of drug(s). The experiment ended when the control reached 100% confluence. The procedure to stain the wells is as follows: removed all media from the cells, added 100% methanol to sufficiently cover the bottom of the well (200 µL for 12-well) and incubated for 20 minutes at room temperature, then wells were washed with water for 1 min and stained with 1% crystal violet (CV) for 10 minutes at room temperature, wells are then washed with water until excess dye is removed. Plates were inverted on tissue paper and left to dry overnight. Once dried, plates were imaged on

the Bio-Rad ChemiDoc MP. 200 µL of 10% acetic acid was added to each well of the 24-well plate and incubated for 20 minutes at room temperature to solubilize the CV. 200 µL of the acetic acid/CV mixture were transferred to standard 96-well plates and absorbance was measured at 590nm using the BioTek Synergy HTX multiplate reader. The data were analyzed by averaging the duplicate wells for each concentration, then subtracting the absorbance values of the blank wells from the treated wells. All values were then normalized to the DMSO treated wells. IC_{50} values were determined using GraphPad Prism (GraphPad, San Diego, CA). Studies were completed ≥ 3 times.

Drug Synergism Studies

Using the Multiflo liquid handling dispensing system, cell lines were plated in 96 (or 384-well plates at a density of 3,000 (96-well, cat no: 165305; Thermo Scientific, cat no: 4515, Corning) or 500-1,000 (384-well, cat no: 781091, Greiner-bio One; cat no: 4516, Corning) cells per well in 50 µL (96-well) or 25 µL (384-well) of complete media. For 96-well plates, cells were plated as singlets and plated in quadruplicates for a 384-well format. After seeding, cells were left to incubate overnight for adaptation. For a 384-well format, monotherapy and combination wells were prepared at 200X in DMSO in a 96-well plate (cat no: P-96-450R-C; Corning) to create a stock plate. Briefly, the maximal concentrations for each drug tested were based on the IC50 determinations from monotherapy cell titer glo studies. The stock plate is then diluted 1:20 into an "intermediate plate" containing 90 µL of complete media using the Agilent Bravo Liquid Handler (cat no: 49654, Agilent). The concentrations from the intermediate plate were then diluted 1:10 into the cell (treated) plate – 5 µL of the solution is transferred to 50 µL of media and plates were incubated at 37°C for 72 hours. For the 96-well format, cells were prepared at 4X in DMSO. Then each drug was transferred to the well at a volume of 25 µL: Control wells received 50 µL of media + DMSO, Monotherapy wells received 25 µL of media + 25 µL drug (RAS/MAPKi or RPT04402) , and the combination wells received 25 µL Tram + 25 µL RPT04402. After treating the cells, plates were incubated like the 384-well procedure. For analysis, the Cell Titer Glo protocol mentioned above was carried out for both 2D and 3D conditions.

For combination clonogenic assays, cell lines were seeded at a density of 125-500 cells per well in a 24-well plate for a total volume of 1 mL in complete media. Cells were allowed to adapt and treated 24 hours post seeding. Cells were treated with trametinib or adagrasib as a single treatment or in combination with RPT04402 as concentrations indicated in the figure legends. Inhibitor(s) and media were replenished every 3-4 days. The experiment ended when the control reached 100% confluency. For analysis, the clonogenic assay protocol mentioned above was utilized. Drug synergism was

analyzed using SynergyFinder+ [synergyfinder (synergyfinderplus.org)]. SynergyFinder+ generates synergy scores using the reference models highest single agent (HSA),Bliss, Loewe, and Zero interaction potency (ZIP) models. For our studies, we utilized the HSA model to quantify the degree of synergy compared to the maximum drug response for monotherapies. Studies were completed ≥ 3 times.

Live Dead Staining

2D Adherent Monolayer: Cells were plated at a density of 20,000-50,000 cells per well in 8-well chamber glass slides (cat no: 80841-90; Ibidi) and allowed to adapt 24 hours prior to treatment. Cells were treated with DMSO, trametinib/adagrasib, RPT04402, or the combination for 48 hours and cells were stained according to the Live Dead Cell Viability Kit protocol (cat no: CBA415; Sigma-Aldrich).

3D Spheroids: Cells were seeded in 24-well microcavity Elplasia® plates (cat no: 4441; Corning) at a density of 300 cells per microcavity (554 microcavities per well). Prior to seeding, wells were pre-wet with 500 µL of complete media and spun at 500x g for 1 minute to remove trapped air. 500 µL of desired cell concentration were added to the well for a total volume of 1mL. Cells were incubated for 48 hours prior to treatment to ensure the formation of spheroids. Cells were then treated similarly to the 2D study and allowed to incubate for 72 hours and stained following the Live Dead Cell Viability Kit. To ensure staining of the spheroids, the spent media was carefully aspirated, and the staining solution was added to the cells. Cells were then centrifuged at 500x g for 1 minute and promptly placed into a humidified incubator for 60 minutes at 37°C.

Both studies were imaged using the EVOS™ M5000 Imaging System (cat no: AMF5000, Invitrogen™). Images per treatment condition were taken at 10X magnification. Filters used were: DAPI (nuclei), GFP (calcein-AM), and RFP (Propidium Iodide).

Annexin-FITC and Propidium Iodide Staining by Flow Cytometry

Cells were seeded in 10-cm plates at a density of 1,000,000 cells and treated with appropriate drug (Trametinib, Adagrasib, or RPT04402) concentrations for 48 hours. Cells were collected, washed in 1 mL cold 1X PBS, and resuspended in 1X Annexin Binding buffer and stained according to the Dead Cell Apoptosis Kit with Annexin V FITC and PI kit (cat no: V13242; Thermofisher Scientific). Studies were completed ≥ 3 times.

Mice

Athymic Nude FOXn1^{nu} Homozygous male mice, aged 8-10 weeks, were purchased from Jackson Laboratory. Mice were maintained under specific pathogen-free conditions, and food and water were provided *ab libitum*.

In vivo xenograft studies

For cell-derived xenografts (CDXs), cells were harvested from culture on the day of use, washed once in DPBS, spun down at 300x g for 5 minutes and then resuspended in a 50:50 mixture of Matrigel (cat no: 356234; Corning) and DPBS (cat no: 14190250; Invitrogen). 100 µL of the mixture was subcutaneously injected into the right flank of nude mice. Specific cell lines and numbers are as follows: A549 (5×10^6), H358 (10×10^6), H441 (5×10^6), and H460 (5×10^6). After inoculation, mice were monitored daily, weighed twice per week, and caliper measurements began when tumors were palpable. Tumor volume (TV) was calculated using the formula: $TV = (L \times W^2)/2$. When tumors reached a size of 150-200 mm^3, mice were randomized into specific treatment arms and dosed with vehicle or compounds of choice daily *per os*. Compounds for *in vivo* studies were prepared as described above in "**Compounds**". Note, mice are randomized based on tumor volume for all studies – median tumor volumes and median body weights should be similar between all treatment arms.

For RPT04402 dose efficacy (DE) studies, tumor volume and body weights were measured 2-3 times per week. Mice were treated and monitored daily until the animal tumor burden reached an endpoint TV of 2500 mm^3 or when either the length or width reached 20 in either direction. Combination studies followed the same format as DE studies; however, once all vehicle mice were removed (due to reaching endpoint), studies were converted into survival analyses. Mice continued to be treated daily, and dates were tracked to capture when the RAS/MAPKi or RPT04402 treatment arms reached an endpoint, if at all. The percentage of TV change in the treated versus vehicle control is as follows: $[(T_{final} - T_{initial})/(C_{final} - C_{initial})] \times 100$, where $C_{initial}$ and C_{final} are the mean or median tumor volumes on the first day of treatment and the day indicated for the vehicle control group and $T_{initial}$ and T_{final} are the mean or median tumor volumes on the first day of treatment and the day indicated for the treated group. TV graphs are for DE and combination studies end at the day the final vehicle mouse was euthanized. For combination studies, GraphPad Prism was used to generate Kaplan Meier plots to assess differences between RAS/MAPKi, RPT04402, and combination study arms. Statistical significance for the long-term tumor growth curves was determined by two-way repeated measures ANOVA (assuming equal variances) or mixed-effect analysis (assuming unequal variances) with Tukey's post-hoc analysis. Statistical significance of the differences between

survival curves was determined by the Log-rank (Mantel Cox) test and pairwise comparisons were calculated using GrapPad Prism.

For pharmacodynamic studies, A549 cells were injected subcutaneously in the right flank of male mice as described above. When tumors reached a mean TV of 300 mm^3, mice were randomized into treatments arms and dosed daily *per os* with vehicle or test compounds for 7 days. At indicated time points, mice were sacrificed, and tumors were snap frozen and stored at −80 °C until molecular analysis. <u>*Waterfall Plots*</u>: The percentage of TV change (ΔT) over the duration of the study (or at a specific time) is calculated as follows: $\Delta T = (T_{final} - _{initial}) / T_{initial} \times 100$. Modified RECIST (mRECIST) as a preclinical criteria for response was defined as follows: a complete response (CR) is the lack of a palpable tumor; a partial response (PR) is ≥ 30% decrease in tumor volume; progressive disease (PD) is defined as a > 2-fold increase in tumor burden; stable disease (SD) is defined as neither sufficient tumor volume decrease to qualify as a PR nor sufficient tumor growth to qualify as PD.

For *in vivo* studies, all quantitative data are represented as the mean ± SEM. Researchers were not blinded during the studies.

<u>*Immunohistochemistry:*</u> For TUNEL and Ki67staining following pharmacodynamic studies, tumor tissue slides were deparaffinized and antigen unmasking was performed by boiling the slides in 10 mM Citrate Buffer (cat no: H-3300, Vector Laboratories) for 10 minutes. After two washes with cold 1X PBS, TUNEL staining was carried out using the ApopTag Fluorescein *In Situ* Apoptosis Detection Kit (cat no: S7110, MilliporeSigma). For Ki67 staining, slides were washed with cold 1x PBS, then blocked with Animal-Free Blocker ® and Diluent, R.T.U (cat no: SP-5035-100, Vector Laboratories) for 1 hour. After blocking, slides were washed once with cold 1x PBS and then incubated with Ki67 at a 1:300 dilution in Animal-Free Blocker ® and Diluent o/n at 4°C. Following incubation, slides were washed 3x with 1x PBS for 5 min, then incubated with Alexa-Fluor 488 (cat no: A11034, Thermofisher Scientific) for 30 minutes. Following the final wash steps in both studies, slides were then mounted using VectaShield® Vibrance™ Antifade mounting medium with DAPI (cat no: H-188- 10; Vector Laboratories). For each treatment group, 10-15 pictures were captured at 10X magnification using the EVOS500 microscope. The following filters were used: DAPI (nuclei), GFP (TUNEL or Ki67-positive cells). Data analysis was performed using the *Cell Detection* protocol in QuPath software (ref). The percentage of TUNEL or Ki67-positive was calculated as follows: [(Green − Blue)/(Blue)] × 100, where "Green" represents the number of TUNEL or Ki67-positive cells and "Blue" represents the total number of cells (2). Statistical significance between

groups was determined by two-way ANOVA with Tukey's post-hoc analysis for pairwise comparisons.

Key resources table

Reagents or Resources	Application	Source	Identifier
**Antibodies**			
PPP2R3A (PR130)	WB	Thermo Fisher Scientific	Cat# PA530127; RRID: AB_2547601
PP2A-B56α (3A6-F3) C-term	WB	RAPPTA	N/A
PP2A-B56β (E-6)	WB	Santa Cruz Biotechnology	Cat# SC-515676; RRID: AB_3676291
PPP2R5E (PP2A-B56ε) (EPR17147)	WB	Abcam	Cat# ab198500; RRID:AB_3676292
PPP2R5D (PP2A-B56δ) (EPR15617)	WB	Abcam	Cat# ab188323; RRID:AB_3065198
Anti-Methyl-PP2A-C (7C10)	WB	Ogris Lab	Reference - 3
Anti-PP2AC	WB	Abcam	Cat# ab106262; RRID:AB_10860464
Anti-LCMT1 (6D9-G3)	WB	RAPPTA	N/A
Anti-PME1 (8A6-F8)	WB	Millipore-Sigma	Cat# MABC1183; RRID:AB_3676294
PPP2R1A (PP2A-Aα)	WB	Thermo Fisher Scientific	Cat# PA5-27643; RRID:AB_2545119
V5-Tag (D3H8Q)	WB, IP	Cell Signaling Technology	Cat# 13202; RRID: AB_2687461
Vinculin (7F9)	WB	Santa Cruz Biotechnology	Cat# SC-73614; RRID: AB_1131294
Normal Mouse IgG	WB, IP	Santa Cruz Biotechnology	Cat# sc-2025; RRID:AB_737182
Phospho-p44/42 MAPK (ERK1/2) (T202/Y204)	WB	Cell Signaling Technology	Cat# 4370; RRID:AB_2315112
p44/42 MAPK (ERK1/2) (LE4F12)	WB	Cell Signaling Technology	Cat# 4696; RRID: AB_390780
Phospho AKT1 (pT308)	WB	Cell Signaling Technology	Cat# 9275; RRID:AB_329828
Phospho AKT1 (pS473)	WB	Cell Signaling Technology	Cat# 4060; RRID:AB_2315049
AKT1 (pan) (40D4)	WB	Cell Signaling Technology	Cat# 2920; RRID:AB_1147620

**Ki-67 (D3B5) Rabbit mAB**	**WB**	**Cell Signaling Technology**	Cat#9121T<br>RRID: AB_2687446
Peroxidase-AffiniPure Goat Anti-Mouse IgG (H+L)	WB, Secondary	Jackson ImmunoResearch Labs	Cat# 115-035-003; RRID:AB_10015289
Mouse Anti-Goat IgG for IP (HRP)	WB, Secondary	Abcam	Cat# ab157532; RRID: AB_3676695
Amersham ECL Rabbit IgG, HRP-linked whole Ab (from donkey)	WB, Secondary	Cytiva	Cat# GENA934; RRID:AB_2722659
Goat anti-Rabbit IgG (H+L)<br>Alexa Fluor 488	WB, Secondary	Thermofisher Scientific	Cat # A11034<br>RRID: AB_2576217
**Plasmids**			
		**Source**	**Catalog Number**
pLX304-V5-PPP2R1A (PP2A Aa)		DNASU	HsCD00444402
pLX304-V5-LCMT1		DNASU	HsCD00441432
pLentiCRISPR V2 Empty Vector		GenScript	SC1098
PPP2R5A pLentiCRISPR V2 gRNA 1		GenScript	SC1098
PPP2R5A pLentiCRISPR V2 gRNA 2		GenScript	SC1098
LCMT1 pLentiCRISPR V2 gRNA 1		GenScript	SC1098
LCMT1 pLentiCRISPR V2 gRNA 2		GenScript	SC1098
**Drugs**			
		**Source**	**Catalog Number**
Trametinib		Selleck Chemicals	S2673
Selumetinib		Selleck Chemicals	S1008
Adagrasib		MedChem Express	HY-130149
Sotorasib (AMG510)		Selleck Chemicals	S8830
Dimethyl Sulfoxide		Fisher Scientific	BP231-100
Blasticidin		Invivogen	ant-bl-5b
Puromycin		Invivogen	ant-pr-1
Penicillin-Streptomycin (10,000U/mL) (100X)		Thermo Fisher Scientific	15140122
**Experimental models: Mice Strains**			
		**Source**	**Strain**
Athymic *Foxn1*$^{nu/nu}$ Mice (male)		The Jackson Laboratory	002019

NOD.Cg-*Prkdc*^{scid} *Il2rg*^{tm1Wjl}/SzJ (female)	The Jackson Laboratory	*Mouse was used to create the J000096652 cell line; Reference - 4

**Experimental models: Cell lines**			
	**Origin**	**Tissue**	**Catalog Number**
A549	*Homo sapiens*, human, male	NSCLC: Lung Adenocarcinoma	ATCC CCL-185
NCI-H2444	*Homo sapiens*, human, male	NSCLC: unspecified	ATCC CRL-5945
NCI-H358	*Homo sapiens*, human, male	NSCLC: Lung Adenocarcinoma	ATCC CRL-5807
NCI-H23	*Homo sapiens*, human, male	NSCLC: Lung Adenocarcinoma	ATCC CRL-5800
NCI-H1373	*Homo sapiens*, human, male	NSCLC: Lung Adenocarcinoma	ATCC CRL-5866
NCI-H2030	*Homo sapiens*, human, male	NSCLC: Lung Adenocarcinoma	ATCC CRL-5914
6652CL	*Homo sapiens*, human, female	NSCLC: Lung Adenocarcinoma	N/A; Line Developed in Narla Lab

**Reagents/kits**		
	**Source**	**Catalog Number**
Matrigel	Corning	354234
Dulbecco's PBS	Invitrogen	14190250
Gibco Trypsin-EDTA (0.25%) Phenol Red	Fisher Scientific	25200072
LUNA cell counting slides	Logos Biosystem	L12001
Pierce IP Lysis Buffer	Fisher Scientific	PI87787
Phos Stop - phosphatase inhibitor tablets	Millipore Sigma	4906837001
cOmplete EDTA-free Protease Inhibitor Cocktail	Sigma-Aldrich	5892791001
96 well adherent plate	Fischer Scientific	FB012931
Criterion TGX gel(s)	Bio-Rad	5678025, 5678044, 5678094, 5678085
Mini-PROTEAN TGX gel(s)	Bio-Rad	4568044, 4568046, 4568045, 4568096
Nitrocellulose membrane transfer kit	Bio-Rad	1704159 and 1704158
Dry powder milk	Research Products International	M17200
FBS - Reserved Lot	VWR	97068-085
2D Cell Titer Glo Cell Viability Assay	Promega	G9242

CellTier-Glo(R) 3D Cell Viability Assay	Promega	G9683
Live Deda Cell Viability Assay Kit	Sigma-Aldrich	CBA415
Dead Cell Apoptosis Kit with Annexin V FITC and PI	Thermo Scientific	V13242
Apoptag Fluorescein In Situ Apoptosis Detection Kit	Fisher Scientifc	S7110
VECTASHIELD PLUS Antifade Mounting Medium	Vector Laboratories	H-1900
Citrate-Based Antigen Unmasking Solution	Vector Laboratories	H-3300-250
QuikChange II XL site-directed Mutagenesis Kit	Agilent Technologies	200521
Thermo Scientific PageRuler Prestained 180kDa	Fisher Scientific	P126617
8 well chamber, removable	Ibidi	80841-90
Protoglow, ECL	Fisher Scientific	NC1478047
Corning CellBIND 24-well clear well Multiple Well Pates	Corning	3337
Nunc Microwell 96-Well Polystrene Tissue Culture Treated Plates, Black	Thermo Scientific	165305
96-well Black/Clear Round Bottom Ultra-Low Attachment Surface Spheroid Microplate	Corning	4520
CELLSTAR µCLEAR 384 well-plates	Greiner Bio-One	781091
Black/Clear Round Bottom Ultra-Low Attachment Spheroid 384 well-plates	Corning	4516
Sample Buffer, Laemmli 2X Concentrate	Sigma-Aldrich	S3401-10VL
Scienceware FlowMi Cell Strainers for 1000µL Pipet Tips, Bel-Art, Porosity: 40 µm	VWR	10032-802
Scienceware FlowMi Cell Strainers for 1000µL Pipet Tips, Bel-Art, Porosity: 70 µm	VWR	10204-924
Molecular Probes NucBlue Live ReadyProbes Reagent	Fisher Scientific	R37605

**sgRNA sequence for *PPP2R5A* knock out (F: Forward, R: Reverse)**		**Source**
F-1: AACCCACGCTTGAGGCCTCT	F-2: GAGTATGTTTCAACTAATCG	GenScript
R-1: AGAGGCCTCAAGCGTGGGTT	R-2: CGATTAGTTGAAACATACTC	GenScript
**sgRNA sequence for *LCMT1* knock out (F: Forward, R: Reverse)**		**Source**

F-1: CATTGTCAAATTGTCAACCT	F-2: CAGGCATGGATACCACCTTC	GenScript
R-1: AGGTTGACAATTTGACAATG	R-2: GAAGGTGGTATCCATGCCTG	GenScript
**Primer sequence for V5-LCMT1 PAM mutation (F: Forward, R: Reverse)**		**Source**

F: TGTCAACCTTGTGGCAGGCATGATACC (MT PAM in purple)	R: GGTATCATGCCTGCCACAAGGTTGACA	IDT
**Statistical Analysis**		
**Description**	**Statistical test**	Software
Experimental data following a normal distribution was analyzed using various statistical tests, including ordinary one-way and two-way analyses with Tukey's, Dunnett's, or Šídák's tests for multiple comparisons. Each test was conducted with n ≥ 3 biological replicates, and all graphs displaying statistical analyses represent data as mean ± standard error of the mean.	Mixed-effects analysis<br>Log-rank Mantel-Cox test<br>Ordinary one-way/two-way ANOVA<br>Two-tailed T-test	GraphPad Prism,<br>R Studio,<br>SynergyFinder+
**Software**		

	**Source**	**URL**
ImageJ/Fiji	NIH	Fiji
Prism	GraphPad	https://www.graphpad.com/scientific-software/prism/
Image Lab Software	Bio-Rad	https://www.bio-rad.com/en-us/product/image-lab-software?ID=KRE6P5E8Z
QuPath	Center for Cancer Research & Cell Biology at Queen's University Belfast.	Introduction — QuPath 0.6.0-rc3 documentation
SynergyFinder+	Reference - 5	synergyfinder
Rstudio	RStudio Team (2024). RStudio: Integrated Development for R. RStudio, PBC, Boston, MA URL	RStudio Desktop - Posit
FlowJo	TreeStar	https://www.flowjo.com
**Instrumentation**		
ChemiDoc XRS+ Imager	Bio-Rad	
BioTek MultiFlo FX Multimode Dispenser	Agilent	
Bravo Automated Liquid Handling Platform	Agilent	

Agilent BioTek Synergy HTX multimode Plate Reader	Agilent
Sony SH800 FACS Cell Sorter	Sony Biotechnology
Ze5 Analyzer	Bio-Rad
EVOS M5000 Imaging System	Invitrogen
LUNA II Automated Cell Counter	Logos Biosystems

REFERENCES

1. Haanen T. J, et al. Mutant PP2A Induces IGFBP2 Secretion to Promote Development of High-Grade Uterine Cancer. *Cancer Res*. 2025; 85(3), 442–461.
2. Bankhead P, et al. QuPath: Open-source software for digital pathology image analysis. *Sci. Rep*. 2017;7(1), 16878.
3. Frohner IE, Mudrak I, Kronlachner S, Schüchner S, Ogris E. Antibodies recognizing the C terminus of PP2A catalytic subunit are unsuitable for evaluating PP2A activity and holoenzyme composition. *Sci. Signal*. 2020;13(616).
4. Patient Derived Xenograft ; Accession Number : J000096652
5. Zheng S, Wang W, Aldahdooh J, et al. SynergyFinder Plus: Toward Better Interpretation and Annotation of Drug Combination Screening Datasets. *Genom Proteom Bioinf*. 2022;20(3):587-596.

Supplemental Figures and Figure Legends

Supplemental Figure 1

Supplemental Figure 2 – Related to Figure 1

Supplemental Figure 3

Supplemental Figure 4 – Related to Figure 1

Supplemental Figure 5 – Related to Figure 1 & Supplemental Figure 4

Supplemental Figure 6

Supplemental Figure 7

Supplemental Figure 8 – Related to Figure 2

Supplemental Figure 9

Supplemental Figure 10

Supplemental Figure 11 – Related to Figure 3

Supplemental Figure 12 – Related to Figure 4

Supplemental Figure 13 – Related to Figure 5

Supplemental Figure 14

Supplemental Figure 15

Supplemental Figure 16 – Related to Figure 6

Supplemental Figure 17

Supplemental Figure 18 – Related to Figure 6

Supplemental Figure 19 – Related to Figure 7

Supplemental Figure 20 – Related to Figure 7

Supplemental Figure 1

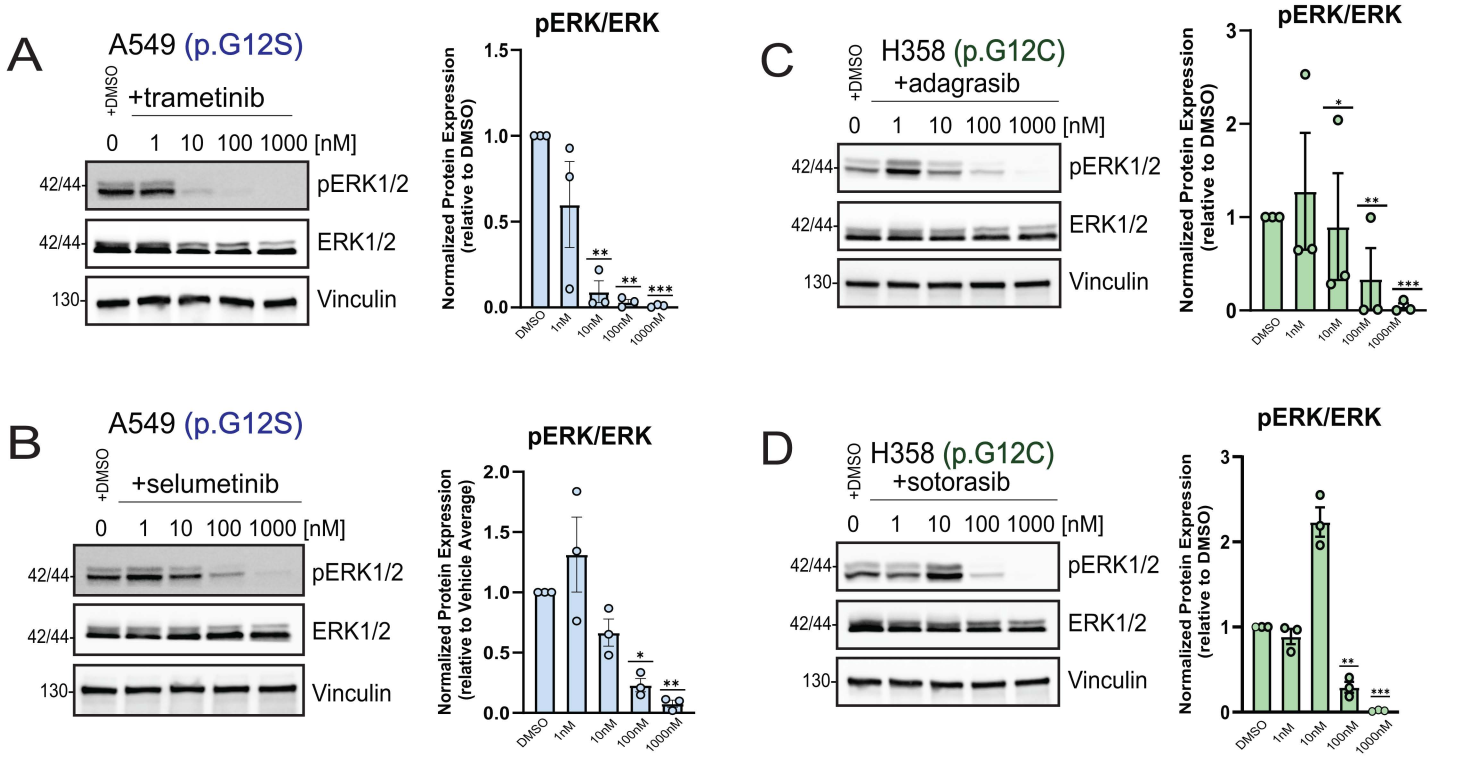

Supplemental Figure 1. RAS/MAPK inhibitor dose-response study in A549 and NCI-H358 cell lines. (**A & B**) Western blot and quantification of pERK levels from A549 lysates treated with trametinib **(A)** (1-1,000 nM) or selumetinib **(B)** (1-1,000 nM) for 2 hours. (**C & D**) Western blot and quantification of pERK levels from NCI-H358 lysates treated with adagrasib **(C)** (1-1000 nM) or sotorasib **(D)** (1-1,000 nM) for 2 hours. pERK was normalized to total ERK levels. Densitometry data is expressed relative to the "DMSO" condition. Data are represented as mean ± SE ($n \geq 3$). Statistical significance was determined by one-way ANOVA with Tukey's post-hoc analysis: *$p \leq 0.05$, **$p \leq 0.01$,***$p \leq 0.001$,****$p \leq 0.0001$.

Supplemental Figure 2

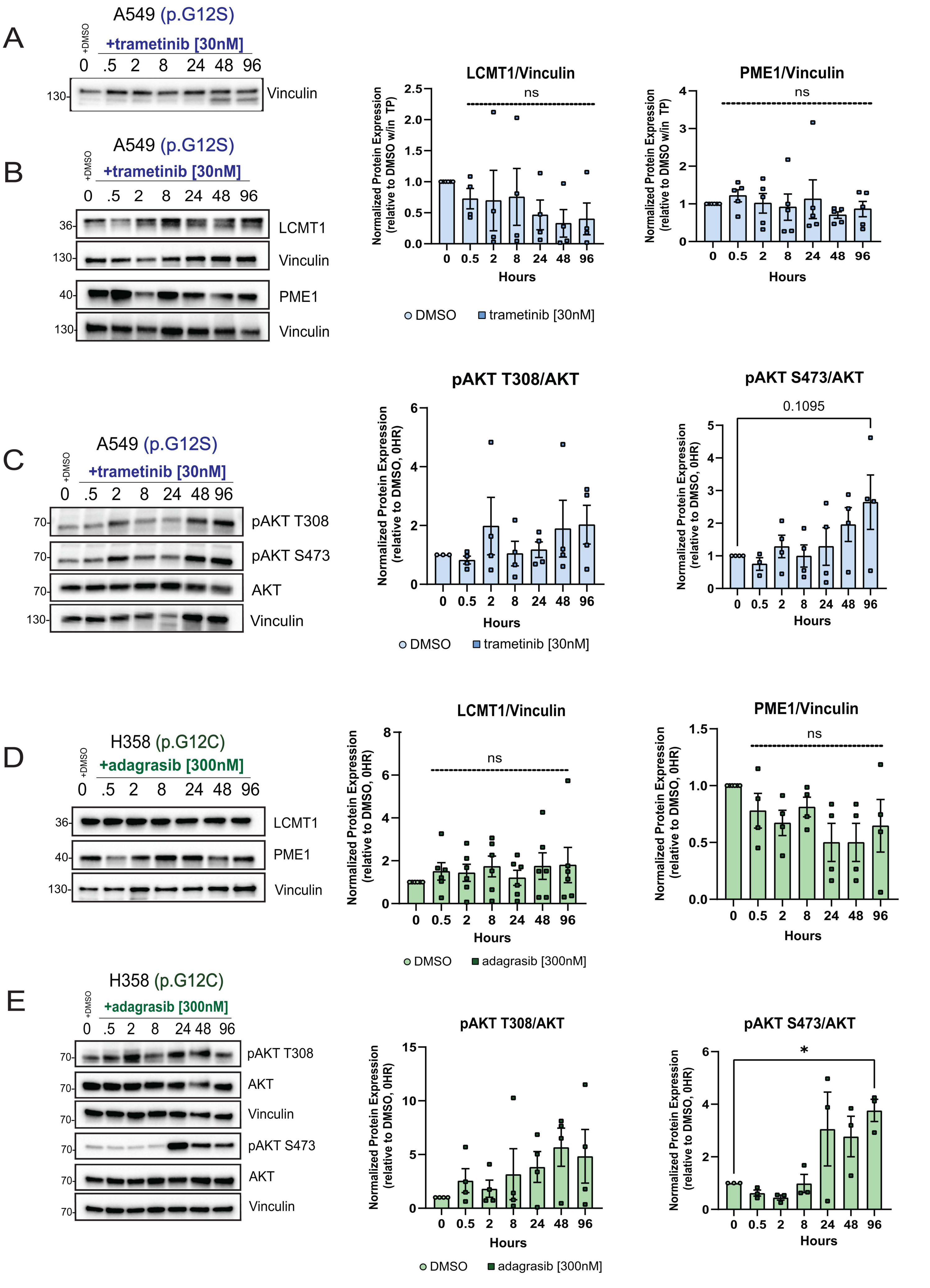

Supplemental Figure 2. RAS/MAPK inhibition had no effect on LCMT1 and PME1 protein levels. (**A**) Westen blot of vinculin used as the loading control for western blots in Figure 1A. (**B**) Western blot analyses of LCMT1 and PME1 from A549 lysates treated with trametinib (30 nM) for the indicated time-points. (**C**) Western blot analyses of pAKT (pT308 and pS473) from A549 lysates treated with trametinib (30 nM) for the indicated time-points. (**D**) Western blot analyses of LCMT1 and PME1 in NCI- H358 lysates treated with adagrasib (300 nM) for the indicated time-points. (**E**) Western blot analyses of pAKT (pT308 and pS473) from NCI-H358 treated with adagrasib (300 nM) for the indicated time-points. LCMT1 and PME1 levels were normalized to Vinculin. pAKT (pT308 and pS473) was normalized to total AKT levels. Densitometry data is expressed relative to the "DMSO, 0HR" condition. Quantitation of western blots are located to the right of the blots. Data are represented as mean ± SE ($n \geq 3$). Statistical significance was determined by one-way ANOVA with Dunnett's post-hoc analysis: *$p \leq 0.05$, ***$p \leq 0.001$, ns = not statistically significant.

Supplemental Figure 3

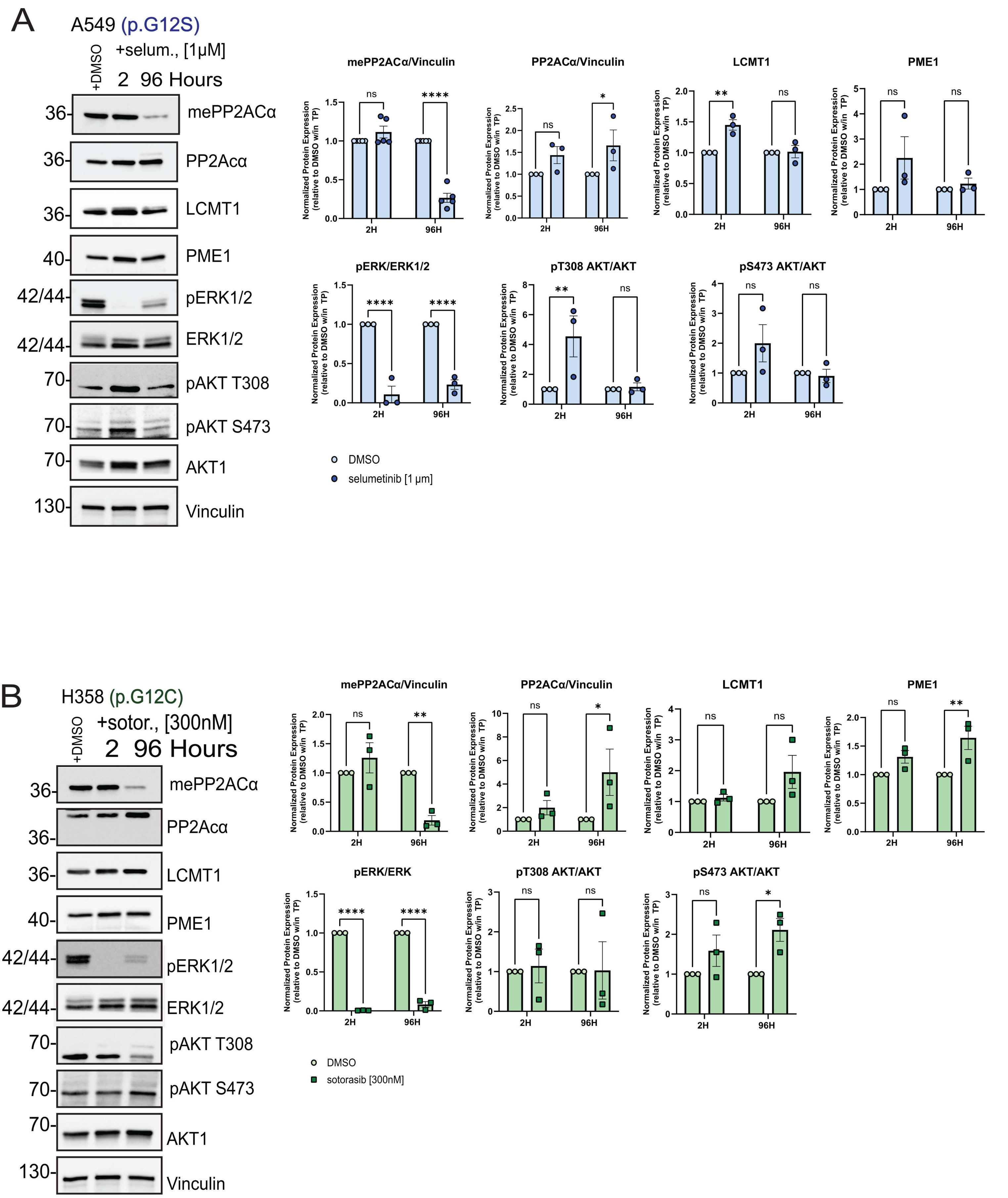

Supplemental Figure 3. Selumetinib and sotorasib reduced methylated PP2AC*α* in vitro.
(**A**) Western blot analyses and the quantitation of pERK, tERK, mePP2ACα, PP2ACα, LCMT1, PME1, and p-AKT (pT308 and pS473) in A549 cells treated with selumetinib (1 μM) for the indicated time points. (**B**) Western blot analysis and the quantitation of the same target proteins mentioned in (**A**) in NCI- H358 cells treated with sotorasib (300 nM) for the indicated time points. pERK and pAKT are normalized to tERK and t AKT, respectively. mePP2ACα, and PP2ACα are normalized to the loading control, Vinculin. Densitometry data is expressed relative the "DMSO, 0hr" condition. Data in graphs, located on the right, are represented as mean ± SE (n ≥ 3). Statistical significance was determined by one-way ANOVA with Šídák's multiple comparisons test: *p≤0.05, ****p≤0.0001, ns = not statistically significant. Note, "DMSO" is represented twice on the graphs for simplicity; but data represent the same 'control' for 2- and 96-hour time-points as both time-points were collected simultaneously.

Supplemental Figure 4

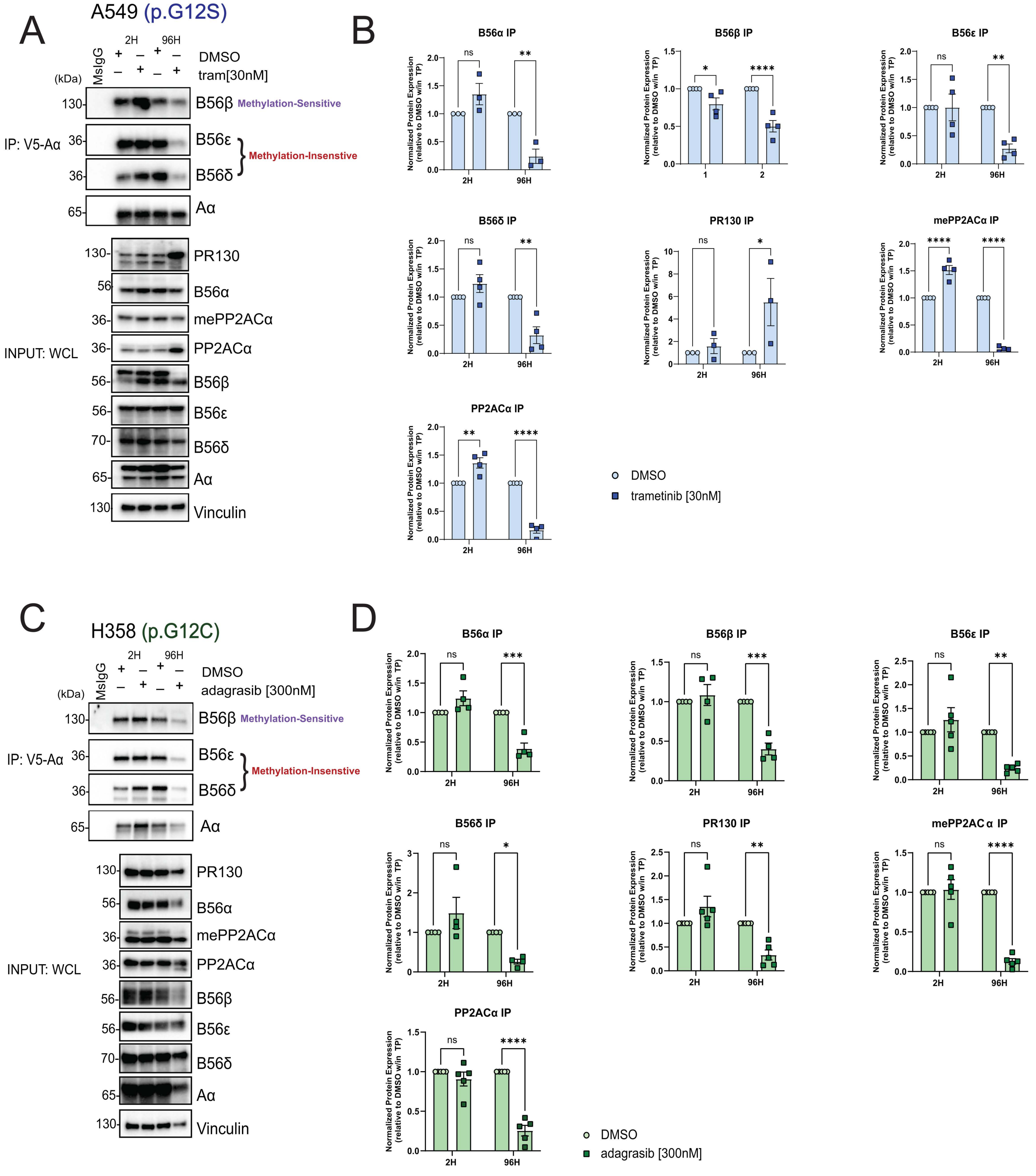

Supplemental Figure 4. RAS/MAPK inhibition destabilized PP2A heterotrimers in vitro.
(**A & B**) Western blot analyses and quantification of B56β, B56ε, B56δ, and Aα from V5-tagged PP2A-Aα A549 lysates following treatment with trametinib (30 nM) for the indicated time points. (**C & D**) Western blot analyses and the quantification of the same target proteins mentioned in (**A**) in NCI-H358 cells treated with Adagrasib (300 nM) for the indicated time points. All data are normalized to V5 relative to DMSO control at each time point. Quantitation of densitometry data are presented as mean ± SE ($n \geq 3$). Statistical significance was determined by two-way ANOVA with Šídák's multiple comparisons test : ns = not significant, *$p \leq 0.05$, ***$p \leq 0.001$, ****$p \leq 0.0001$.

Supplemental Figure 5

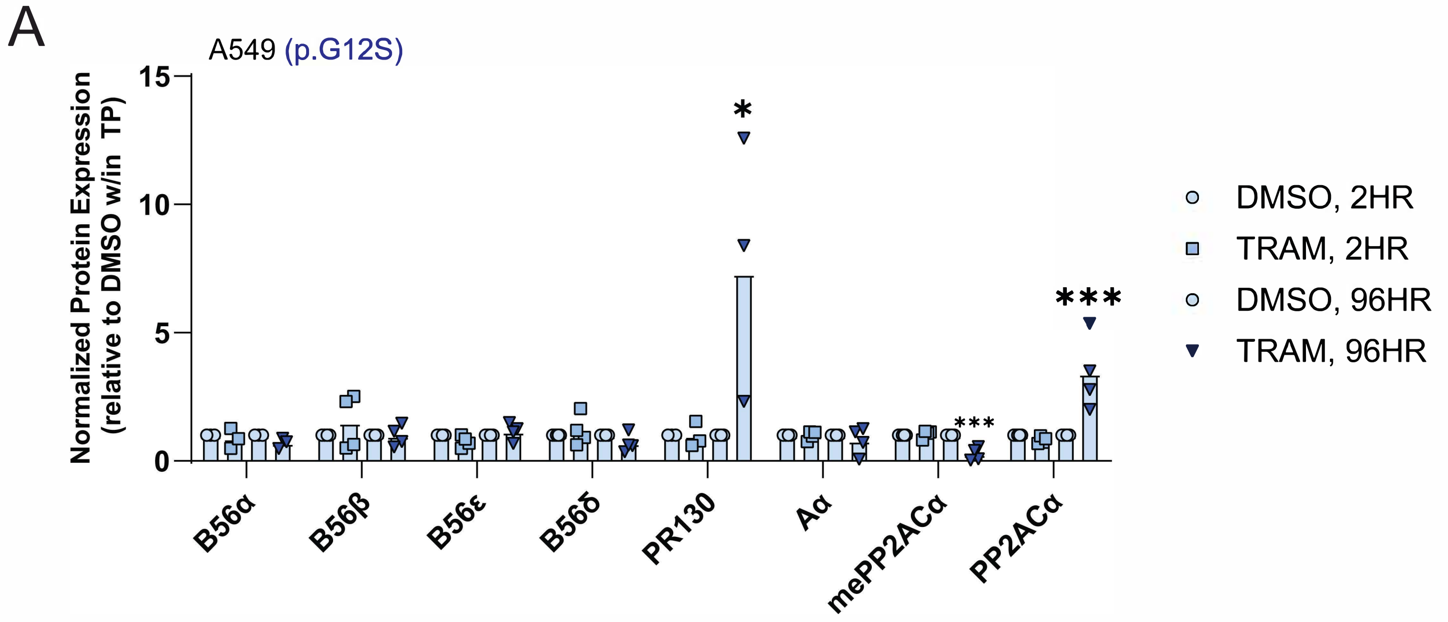

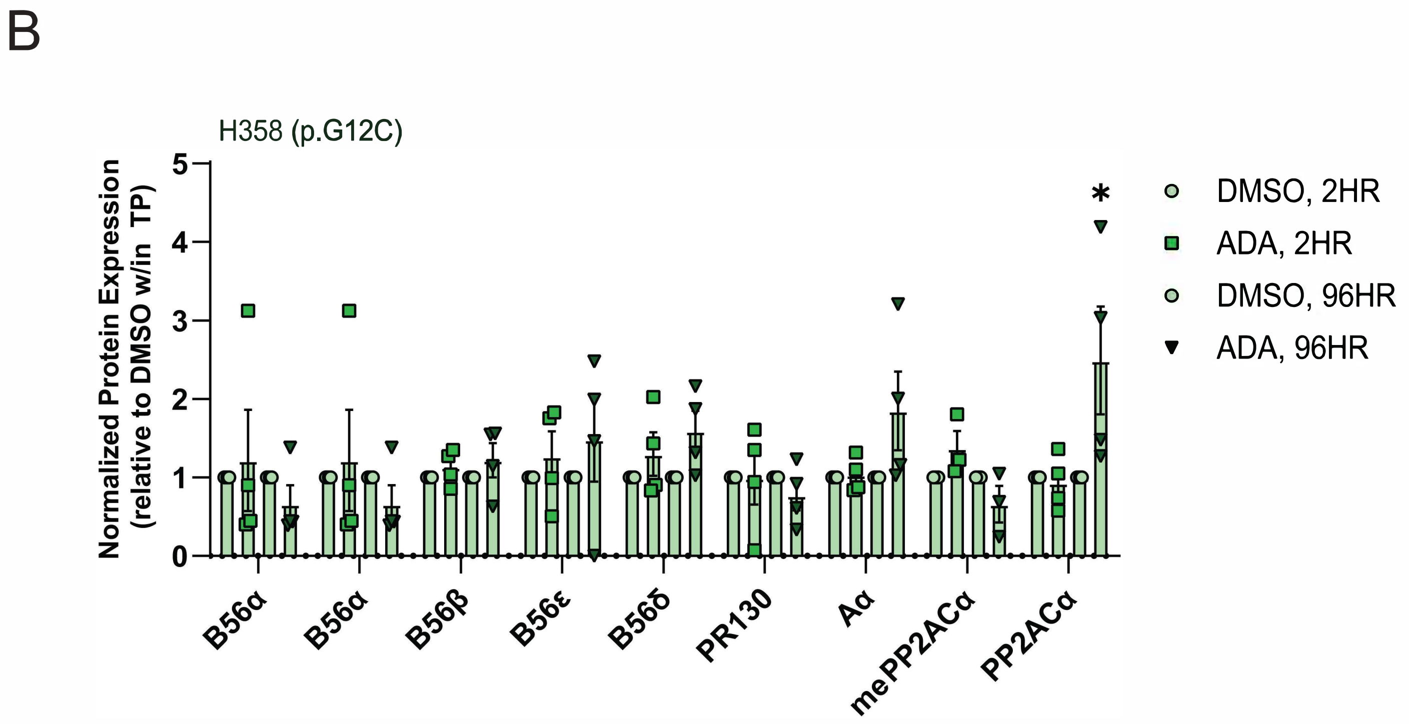

Supplemental Figure 5. Quantification of PP2A components in A549 and NCI-H358 cells following Co-IP

(**A**) Quantitation of B56 regulatory subunits and Aα from V5-tagged PP2A-Aα A549 "INPUT" whole cell lysate (WCL) western blots following treatment with trametinib (30 nM) for the indicated time points as shown in Supplemental Figure 4A. (**B**) Quantitation of the same target proteins in (**A**) from V5-tagged PP2A-Aα NCI- H358 "INPUT" WCL western blots following treatment with adagrasib (300 nM) for the indicated time points as shown in Supplemental Figure 4C. Statistical significance was determined by two-way ANOVA with Šídák's multiple comparisons: *$p \leq 0.05$.

Supplemental Figure 6

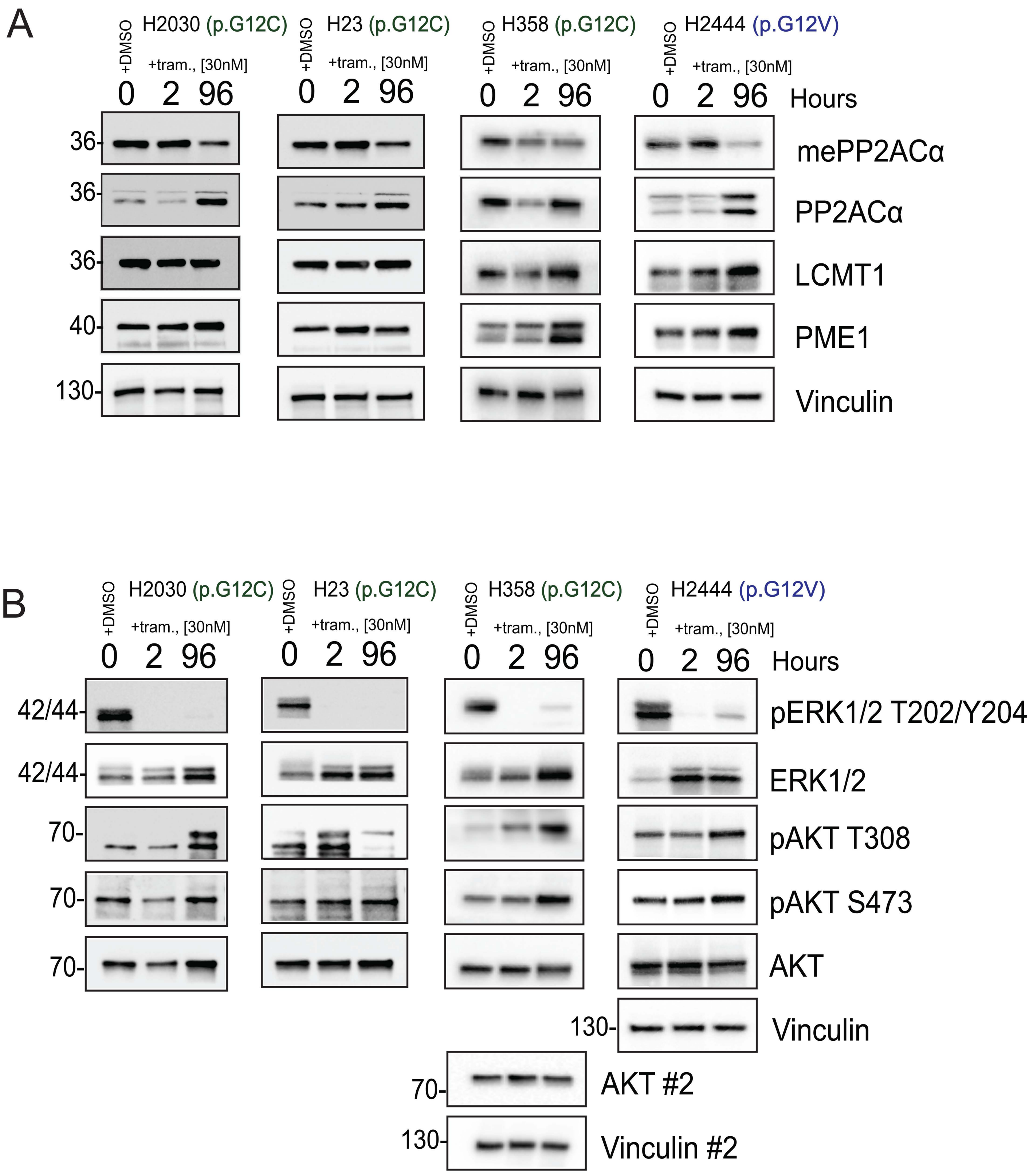

Supplemental Figure 6. Trametinib reduced carboxymethylated PP2AC*α* in multiple *KRAS*- mutant NSCLC cell lines.

(**A**) Western blots of mePP2ACα, PP2ACα, LCMT1, PME1 in the indicated cells lines treated with trametinib (30 nM) for the indicated time points. (**B**) Western blots of pERK, tERK, pAKT (pT308 and pS473), and tAKT in the indicated cell lines treated with trametinib (30 nM) for the indicated time points. Data was analyzed like the other western blots from this study: mePP2ACα, PP2ACα, LCMT1, PME1 are all normalized to Vinculin. pERK and pAKT are normalized to tERK and tAKT, respectively. Densitometry data is expressed relative to the "DMSO, 0HR" condition. Quantitation and statistical analysis of western blots are in the Supporting Data Values file. The "Vinculin" blots for cell lines H2030, H23, and H358 in (**A**) are from the same blots as the signaling blots located in (**B**) unless indicated differently. Data are represented as mean ± SE ($n \geq 3$). Statistical significance was determined by one-way ANOVA with Dunnett's post-hoc analysis: *$p<0.05$,**$p \leq 0.01$, ***$p \leq 0.001$,****$p \leq 0.0001$,ns = not statistically significant.

Supplemental Figure 7

A

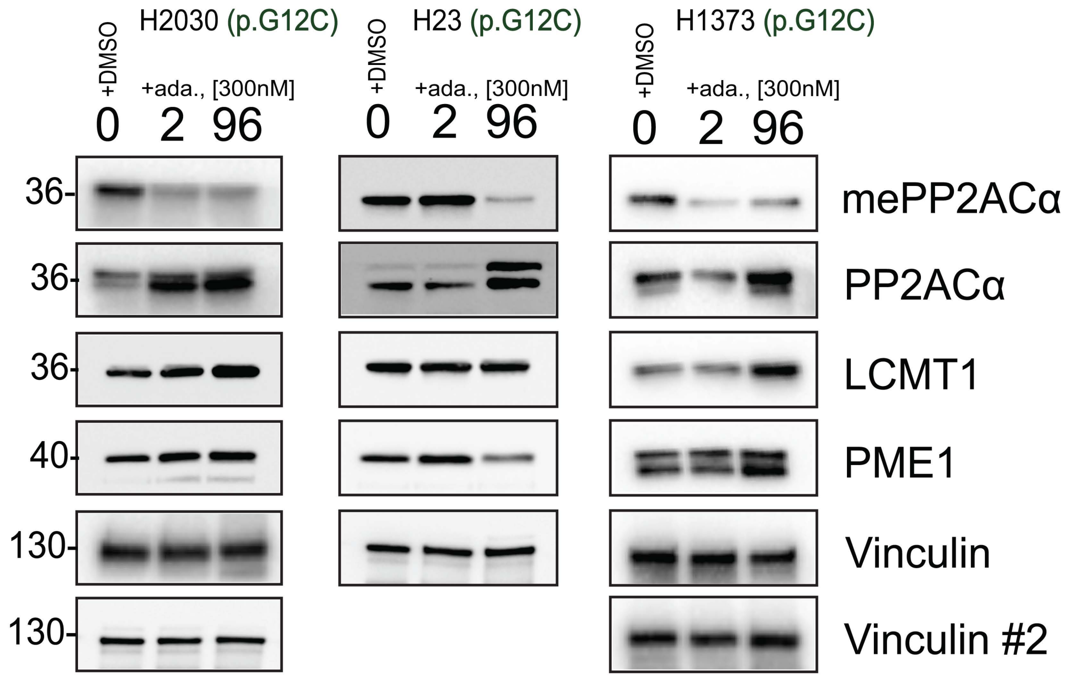

B

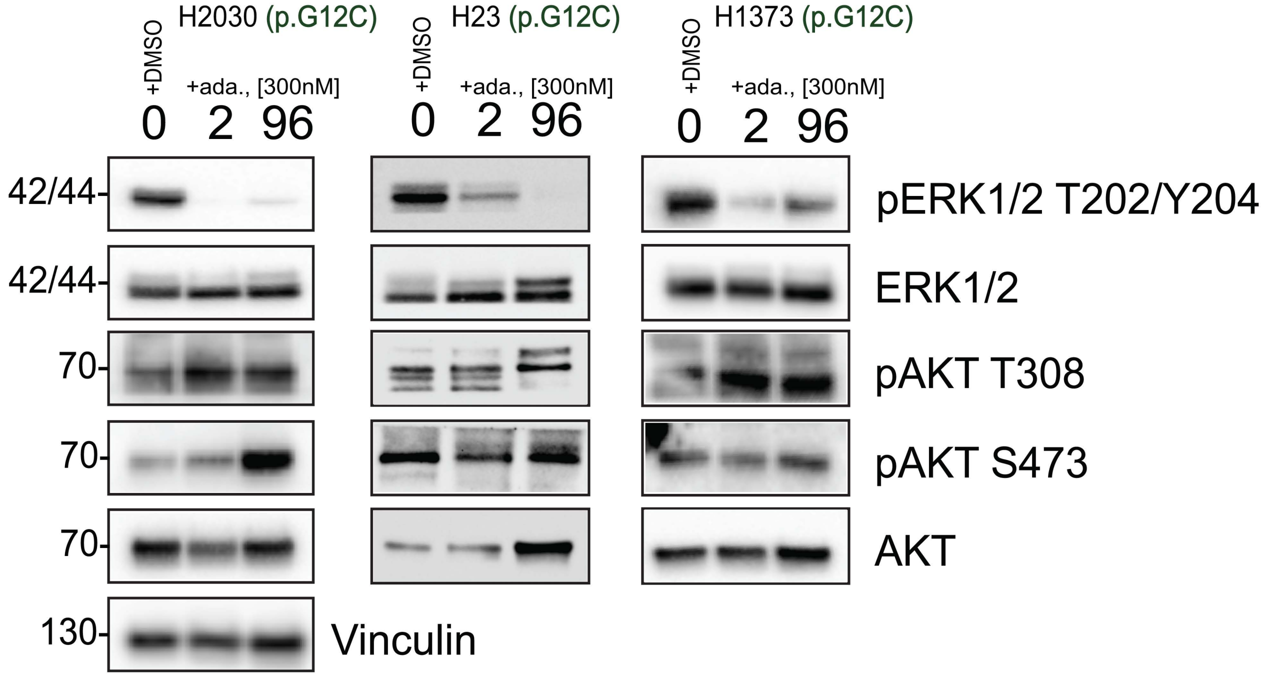

Supplemental Figure 7. Adagrasib reduced carboxymethylated PP2ACα in multiple *KRAS*-mutant NSCLC cell lines.

(**A**) Western blots of mePP2ACα, PP2ACα, LCMT1, PME1 in the indicated cells lines treated with trametinib (30 nM) for the indicated time points. (**B**) Western blots of pERK, tERK, pAKT (pT308 and pS473), and tAKT in the indicated cell lines treated with adagrasib (300 nM) for the indicated time points. Data was analyzed like the other western blots from this study: mePP2ACα, PP2ACα, LCMT1, PME1 are all normalized to Vinculin. pERK and pAKT are normalized to tERK and tAKT, respectively. Densitometry data is expressed relative to the "DMSO, 0HR" condition. Quantification and statistical analysis of western blots are in the Supporting Data Values file. As in the previous figure, some "Vinculin" blots are shared between (A) and (B) and are specified as such in the figure. Data are represented as mean± SEM ($n \geq 3$).

Statistical significance was determined by one-way ANOVA with Dunnett's post-hoc analysis: $^*p<0.05$, $^{**}p\leq0.01$, $^{***}p\leq0.001$, $^{****}p\leq0.0001$, ns = not statistically significant.

Supplemental Figure 8

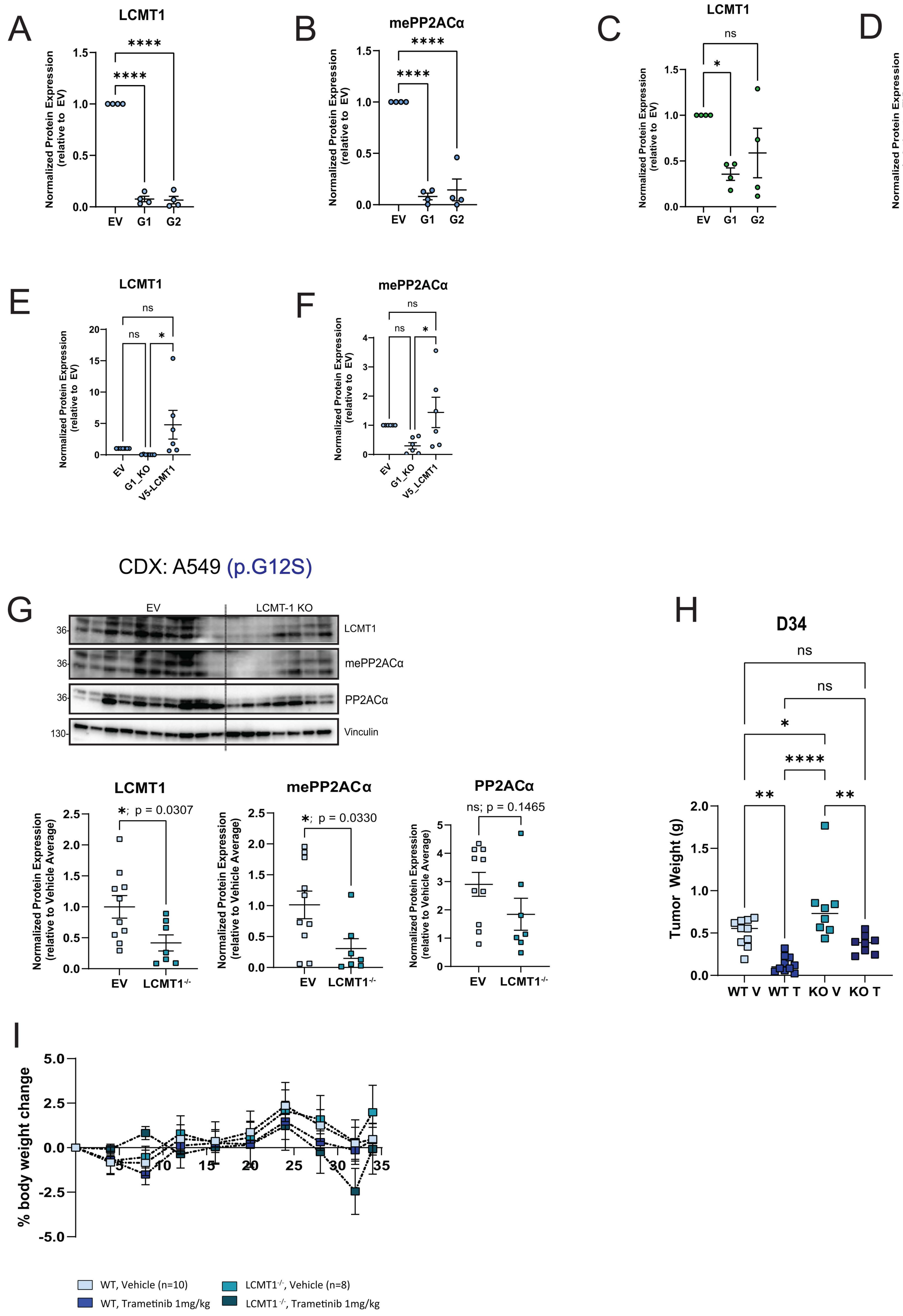

Supplemental Figure 8. LCMT1 KO drives resistance to trametinib in vitro and in vivo.

(**A & B**) Western blot quantification of LCMT1, mePP2ACα, and PP2ACα in A549 cells with empty vector (EV) or LCMT1 knockout (KO) using gRNA #1 and gRNA #2. (**C & D**) Western blot quantification of the same target proteins from (**A & B**) in NCI-H358 cells with EV or LCMT1 KO using gRNA #1 and gRNA #2. (**E &F**) Western blot quantification of the same target proteins as in (**A & B**) in A549 EV, KO, V5-tagged LCMT1 reconstitution cells. Densitometry data is expressed relative to the "EV" condition. Data are represented as mean ± SEM ($n \geq 3$). Statistical significance was determined by one-way ANOVA with Dunnett's post-hoc analysis: *$p \leq 0.05$, **$p \leq 0.01$, ***$p \leq 0.001$, ****$p \leq 0.0001$, ns = not statistically significant. (**G**) Western blot analyses and quantification of LCMT1, mePP2ACα, and PP2ACα from EV and LCMT1 KO Vehicle tumors harvest at the study endpoint. Quantification and statistical analysis of western blots are in the Supporting Data Values file. (**H**) Graph of tumor weights from the tested treatment groups. (**I**) Graph depicting percent body weight change from tested treatment groups.

Supplemental Figure 9

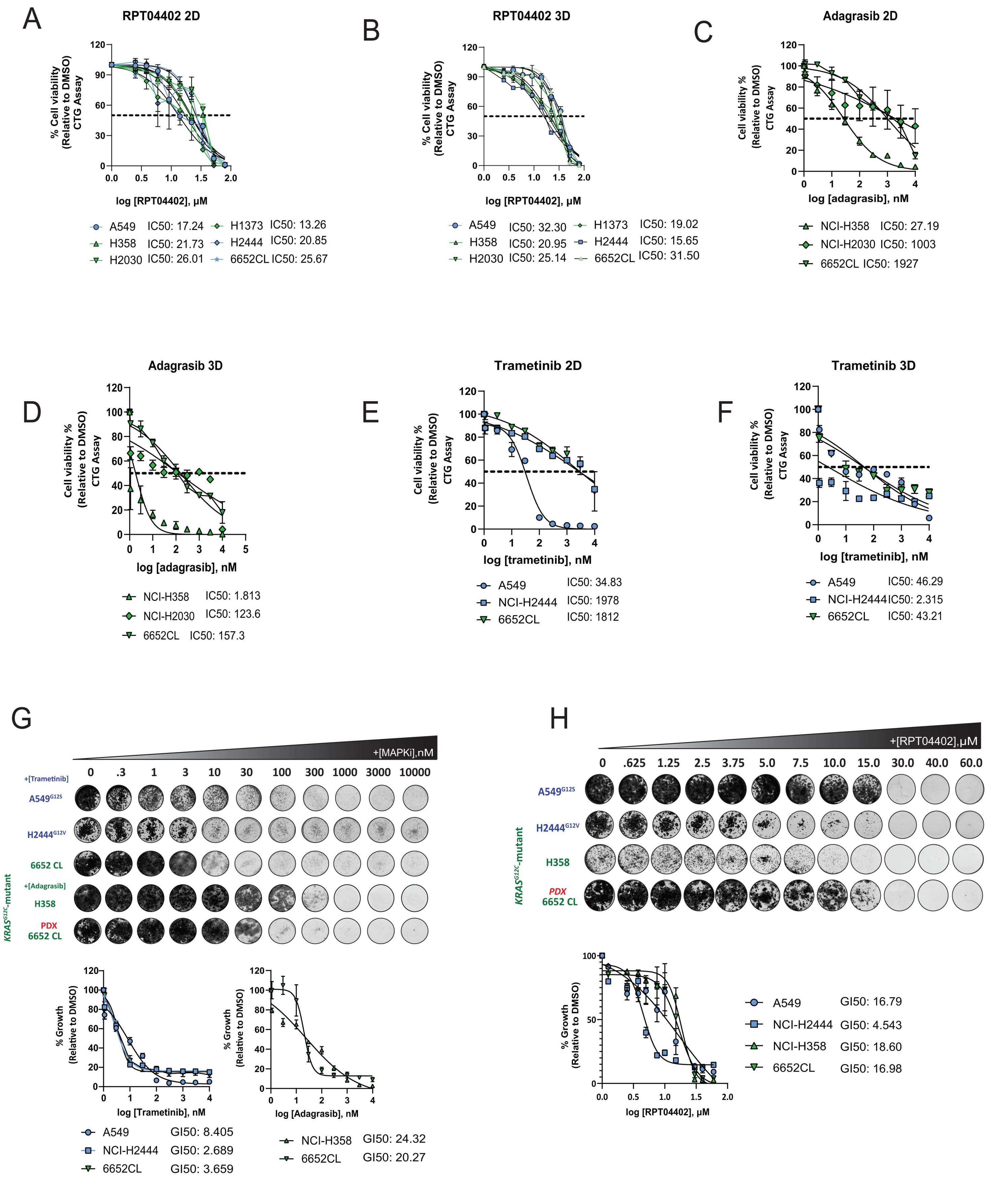

Supplemental Figure 9. Effects of RPT04402, trametinib, and adagrasib on cell viability and cell proliferation.

(**A**) 2D Cell Titer-Glo assay evaluating the effect of RPT04402 on cell viability performed in six *KRAS*-mutant cell lines for 72 hours. (**B**) 3D Cell Titer-Glo assay evaluating the effect of RPT04402 on cell viability in the same cell lines for 72 hours. (**C**) 2D CTG assay (72 hours) performed in KRASG12C-mutant cell lines treated with adagrasib. (**D**) 3D CTG assay measuring the same effect as in (**C**). (**E**) 2D CTG assay (72 hours) performed in three non-KRASG12C-mutant cell lines treated with trametinib. (**F**) 3D CTG assay measuring the same effect as in (**E**). (**G**) Representative image of wells and cell viability curves from clonogenic assays showing A549, NCI-H2444, NCI-H358, and 6652CL cells treated with 0 – 10,000 nM of trametinib or adagrasib for 2 weeks. (**H**) Representative image of wells and cell viability curves from clonogenic assays showing A549, NCI-H2444, NCI-H358, and 6652CL cells treated with 0 – 60 μM of RPT04402 for 2 weeks.

Supplemental Figure 10

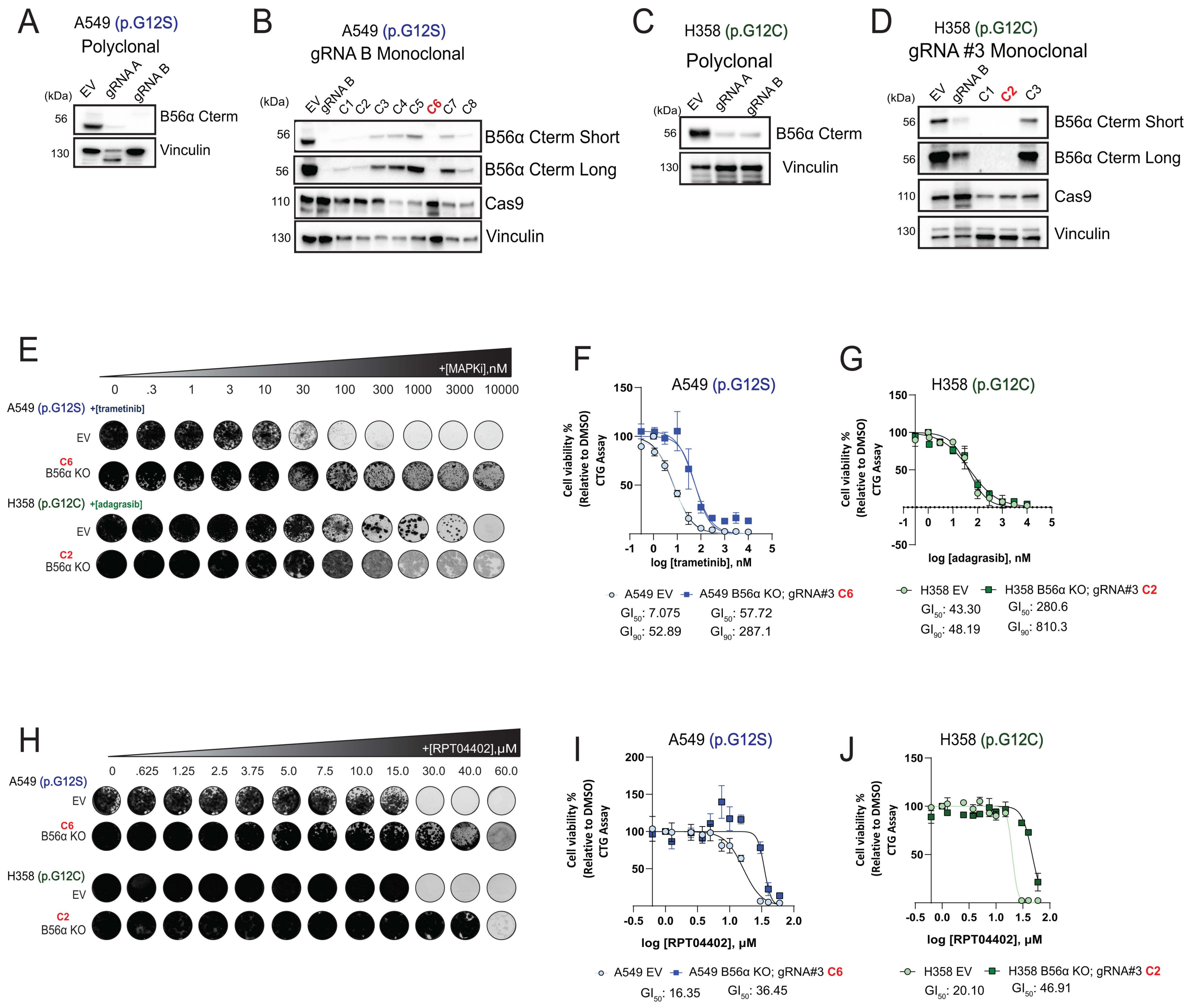

Supplemental Figure 10. B56α knockout increases resistance to trametinib and RPT04402.
(**A**) Western blot analysis of B56α in A549 cells with EV or B56α KO using gRNA A and gRNA B. (**B**) Western blot to confirm knockout of B56α in A549 monoclonal lines. (**C**) Western blot analysis of the same target protein in NCI-H358 cells. (**D**) Western blot to confirm knockout of B56α in NCI-H358 monoclonal lines. (**E, F, G**) Representative image of wells and cell viability curves from clonogenic assays showing A549 (EV and B56α KO C6) and NCI-H358 (EV and B56α KO C2) cells treated with 0 – 10,000 nM of trametinib or adagrasib for 2 weeks. (H, I, J) Representative image of wells and cell viability curves from clonogenic assays showing A549 (EV and B56α KO C6) and NCI-H358 (EV and B56α KO C2) cells treated with 0 – 80 μM of RPT04402 for 2 weeks.

Supplemental Figure 11

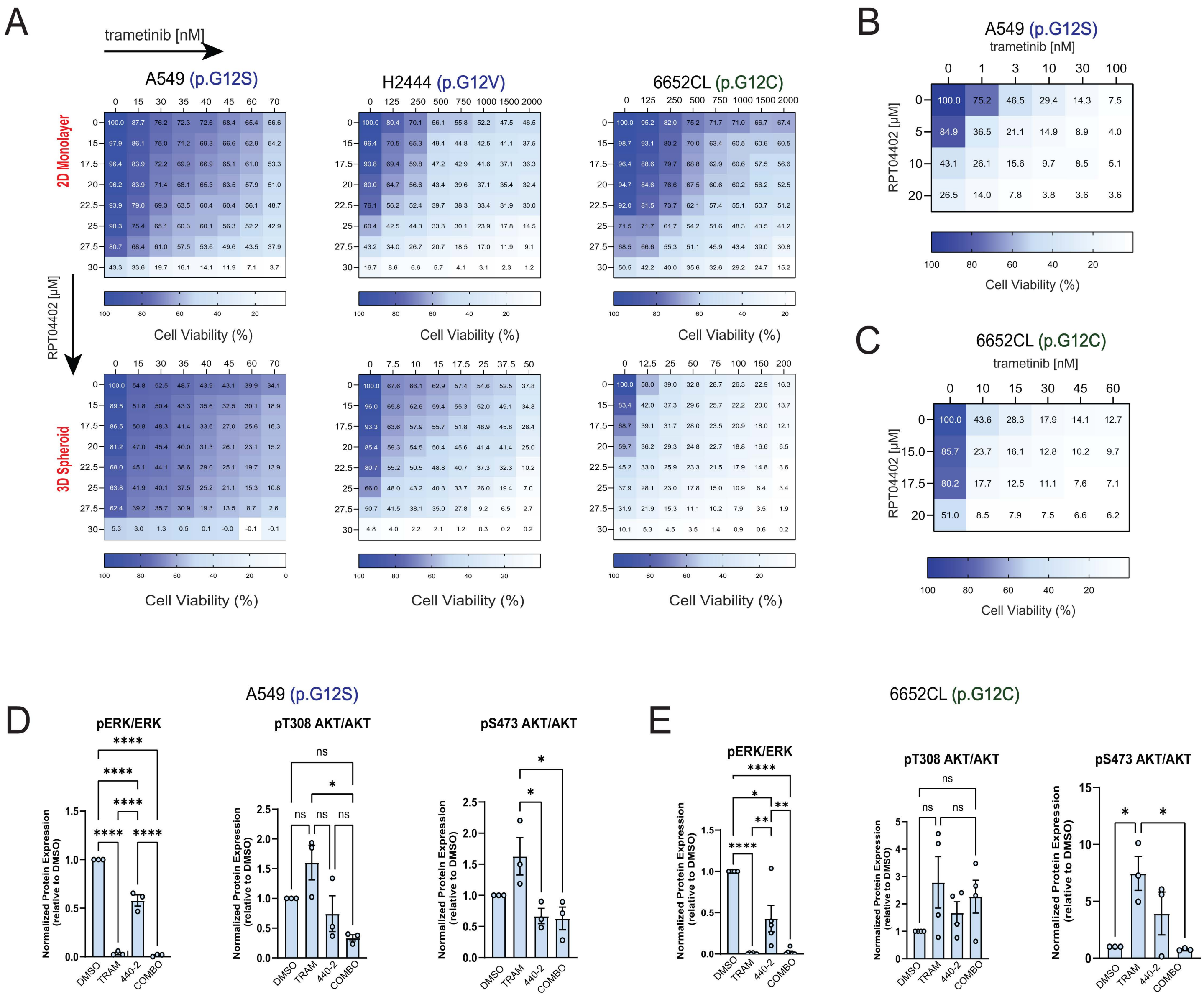

Supplemental Figure 11. Trametinib and RPT04402 synergized to affect cell viability, proliferation, and signaling pathways in *KRAS*-mutant NSCLC.

(**A**) Heatmap of 2D and 3D cell viability values from A549, NCI-H2444, and 6652CL trametinib and RPT04402 synergy studies. Cells were treated with increasing concentrations of RPT04402 or trametinib, either as single treatments or in combination, for 48 hours. (**B**) Heatmap of cell viability from A549 trametinib and RPT04402 combination clonogenic assay. (**C**) Heatmap of cell viability from 6652CL trametinib and RPT04402 combination clonogenic assay. (**D**) Quantification of pERK and pAKT (pT308 and pS473) from western blots of A549 and 6652CL in Figure 3. pERK and pAKT are normalized to tERK and tAKT, respectively. Densitometry data is expressed relative to the "DMSO" condition. Quantification of western blots and statistical analysis are the Supporting Data Values file. Data are represented as mean ± SEM ($n \geq 3$). Statistical significance was determined by one-way ANOVA with Tukey's post-hoc analysis: *$p \leq 0.05$, **$p \leq 0.01$, ****$p \leq 0.0001$, ns = not statistically significant.

Supplemental Figure 12

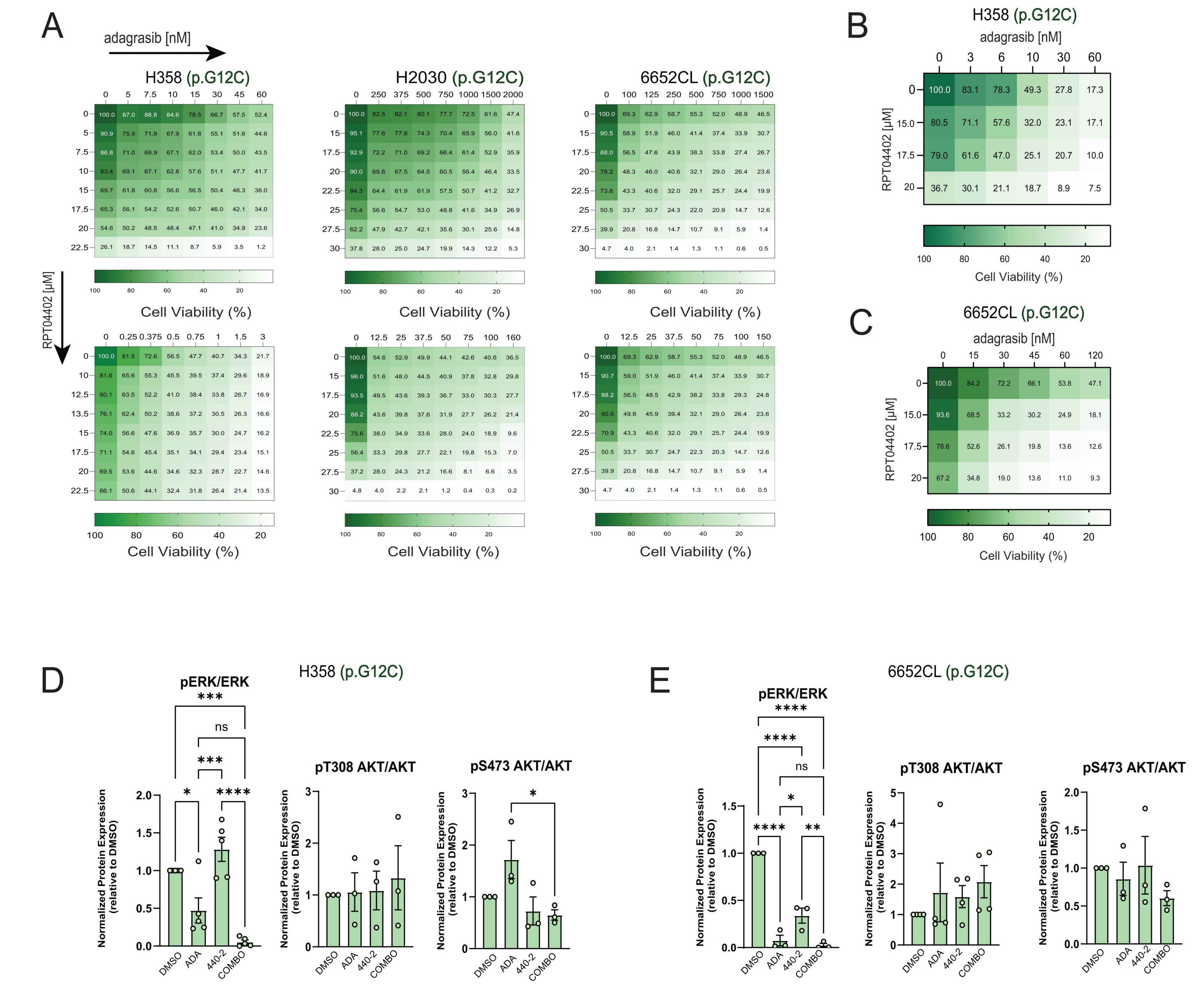

Supplemental Figure 12. Adagrasib and RPT04402 synergized to affect cell viability, proliferation, and signaling pathways in *KRAS*-mutant NSCLC.

(**A**) Heatmap of 2D and 3D cell viability values from NCI-H358, NCI-H2030, and 6652CL adagrasib and RPT04402 synergy studies. Cells were treated with increasing concentrations of RPT04402 or adagrasib, either as single treatments or in combination, for 48 hours. (**B**) Heatmap of cell viability from NCI-H358 adagrasib and RPT04402 combination clonogenic assay. (**C**) Heatmap of cell viability from 6652CL adagrasib and RPT04402 combination clonogenic assay. (**D**) Quantification of pERK and pAKT (pT308 and pS473) from western blots of NCI- H358 and 6652CL in Figure 4. pERK and pAKT are normalized to t-ERK and t-AKT, respectively. Densitometry data is expressed relative to the "DMSO" condition. Quantification of western blots and statistical analysis are in the Supporting Data Values file. Data are represented as mean ± SEM ($n \geq 3$). Statistical significance was determined by one-way ANOVA with Tukey's post-hoc analysis: *$p \leq 0.05$,**$p \leq 0.01$, ****$p \leq 0.0001$, ns = not statistically significant.

Supplemental Figure 13

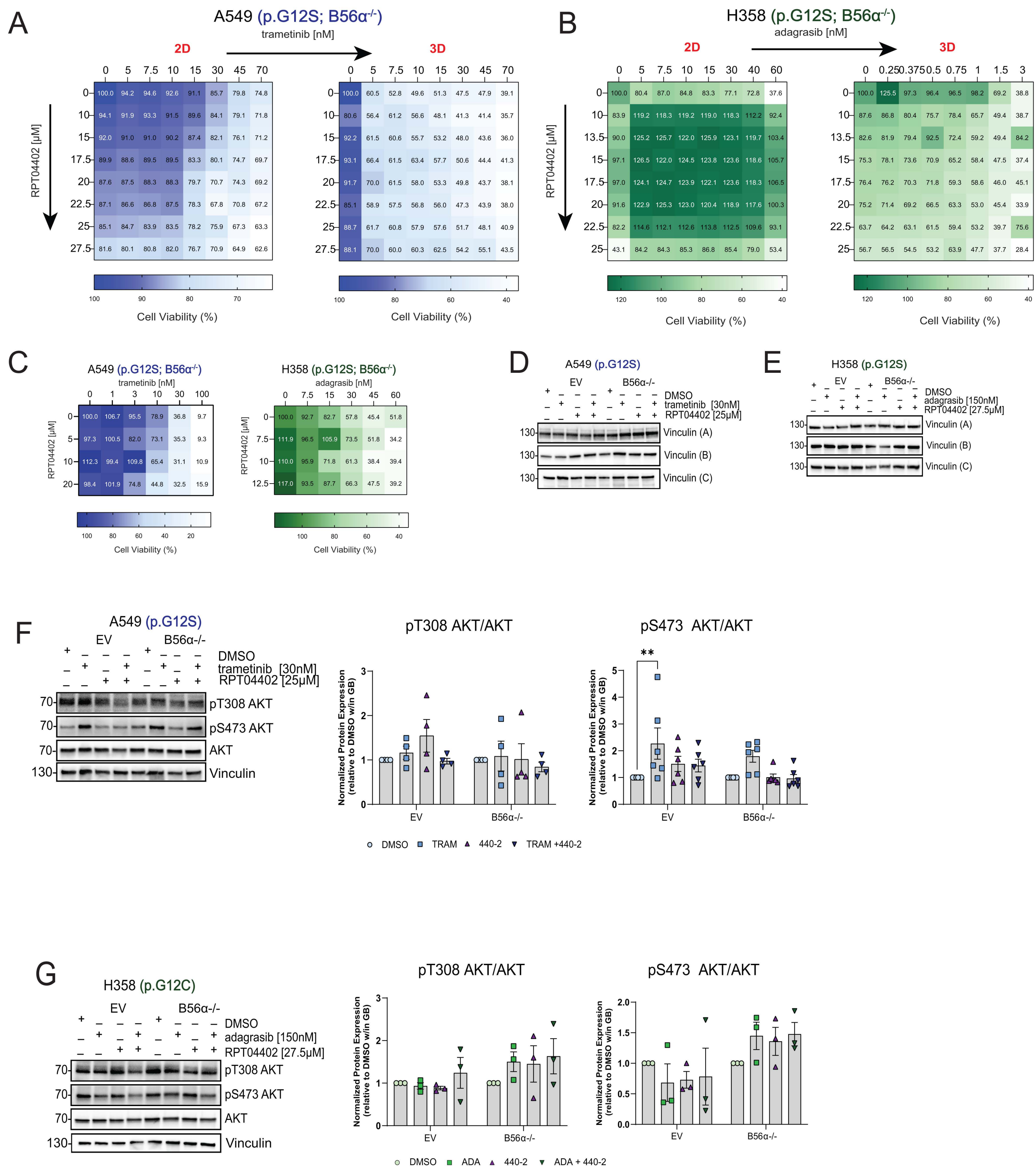

Supplemental Figure 13. B56*α* knockout abolished the synergism between RAS-MAPK inhibitors and RPT04402.

(**A**) Heatmap of 2D and 3D cell viability values from A549 B56α knockout lines. Cells were treated with increasing concentrations of RPT04402 or trametinib, either as single treatments or in combination, for 48 hours. (**B**) Heatmap of 2D and 3D cell viability values from NCI-H358 B56α knockout lines. As in (**A**), NCI-H358 were treated with increasing concentrations of RPT04402, adagrasib, and the combination for 48 hours. (**C**) Heatmap of cell viability values from clonogenic assays of A549 and NCI- H358 B56α knockout cells treated with increasing concentrations of trametinib (A549), adagrasib (NCI- H358), RPT04402, or the combination, and cultured for up to 2 weeks. (**D**) Vinculin loading control western blots for Figure 5E; Vinculin (A) is for B56α, Vinculin (B) is for pERK , Vinculin (C) is for mePP2ACα and total PP2ACα. (**E**) Vinculin loading control western blots for Figure 5F; Vinculin (A) is for B56α, Vinculin (B) is for pERK , Vinculin (C) is for mePP2ACα and total PP2ACα. (**F**) Western blots and quantitation of pAKT (pT308 and pS473), and tAKT from A549 EV and B56α cells treated with DMSO, trametinib, RPT04402 and the combination for 48 hours. (**G**) Western blots and quantification of pAKT (pT308 and pS473), and tAKT from NCI-H358 EV and B56α cells treated with DMSO, adagrasib, RPT04402 and the combination for 48 hours. pAKT is normalized to tAKT. Densitometry data is expressed relative to the "DMSO" condition. Quantification of western blots are located to the right of the blots and statistical analysis are in the Supporting Data Values file. Data are represented as mean ± SE ($n \geq 3$). Statistical significance was determined by one-way ANOVA with Tukey's post-hoc analysis: ns = not statistically significant.

Supplemental Figure 14

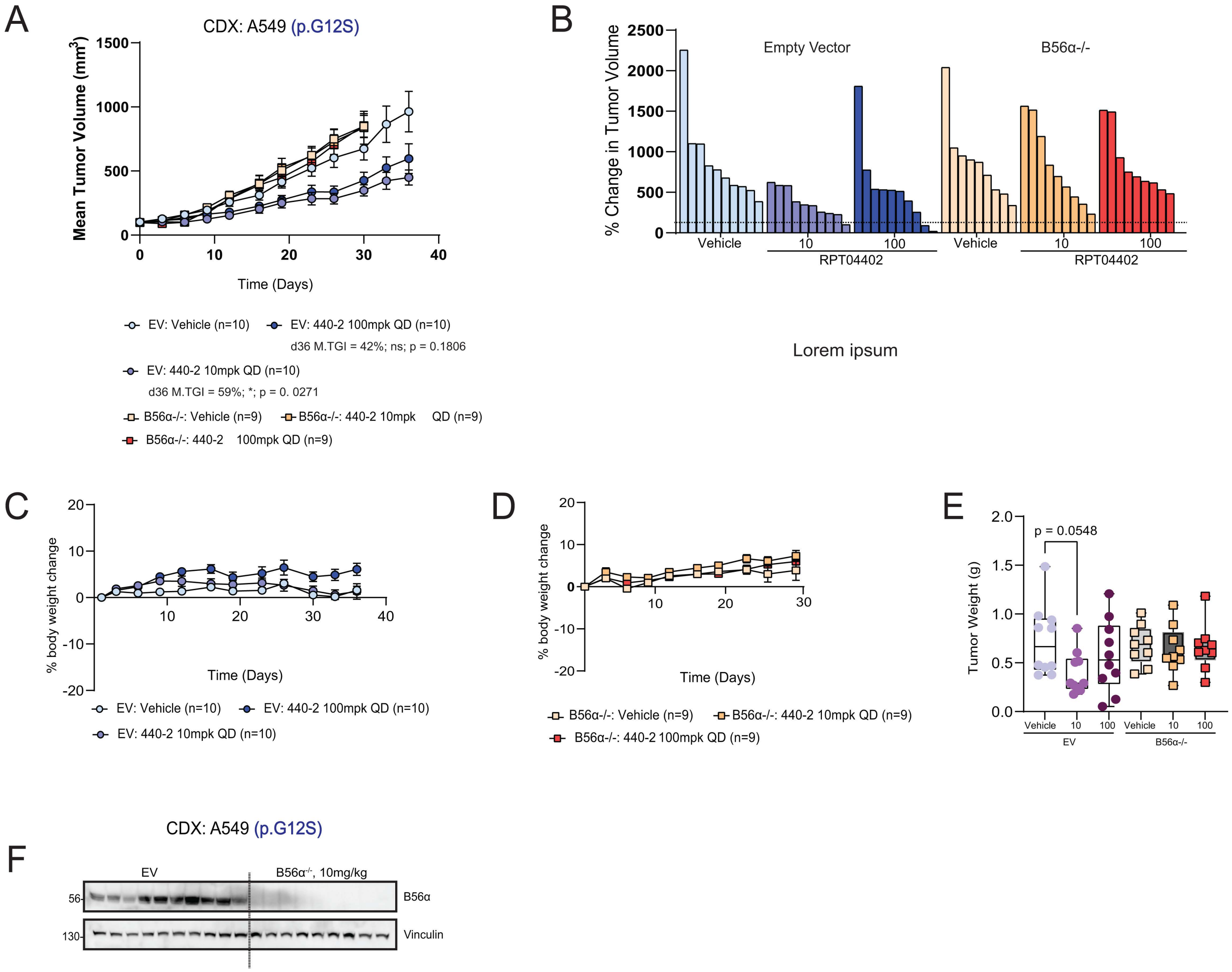

Supplemental Figure 14. Knockout of B56*α* drives resistance to RPT04402 in vivo.

(**A**) Tumor growth curves for EV and B56α$^{-/-}$ A549 CDX mice treated with vehicle (WT: n=10; B56α$^{-/-}$: n = 9), RPT04402 10 mg/kg QD (WT: n=10; B56α$^{-/-}$: n = 9), and RPT04402 100 mg/kg QD (WT: n=10; B56α$^{-/-}$: n = 9). TGI was assessed at the study endpoint for the vehicle groups (36 days). Statistical significance was determined using two-way repeated measures ANOVA with Tukey's multiple comparisons. Only TGI data and statistical significance at the vehicle endpoint are shown on the curve:*$p<0.05$. (**B**) Waterfall plot displaying the percentage change in tumor volume on day 36 for each treatment arm. The plot indicates responses based on RECIST criteria: PD (progressive disease), SD (stable disease), PR (partial response), and CR (complete response). (**C**) Percent body weight change graph from WT-EV A549 CDX mice. (**D**) Percent body weight change graph from A549 B56α$^{-/-}$ CDX mice. (**E**) Graph of tumor weights (grams) from EV and B56α$^{-/-}$ tumors harvested after 36 days of treatment. (**F**) Western blot of B56α from EV and B56α$^{-/-}$ (vehicle) tumors.

Supplemental Figure 15

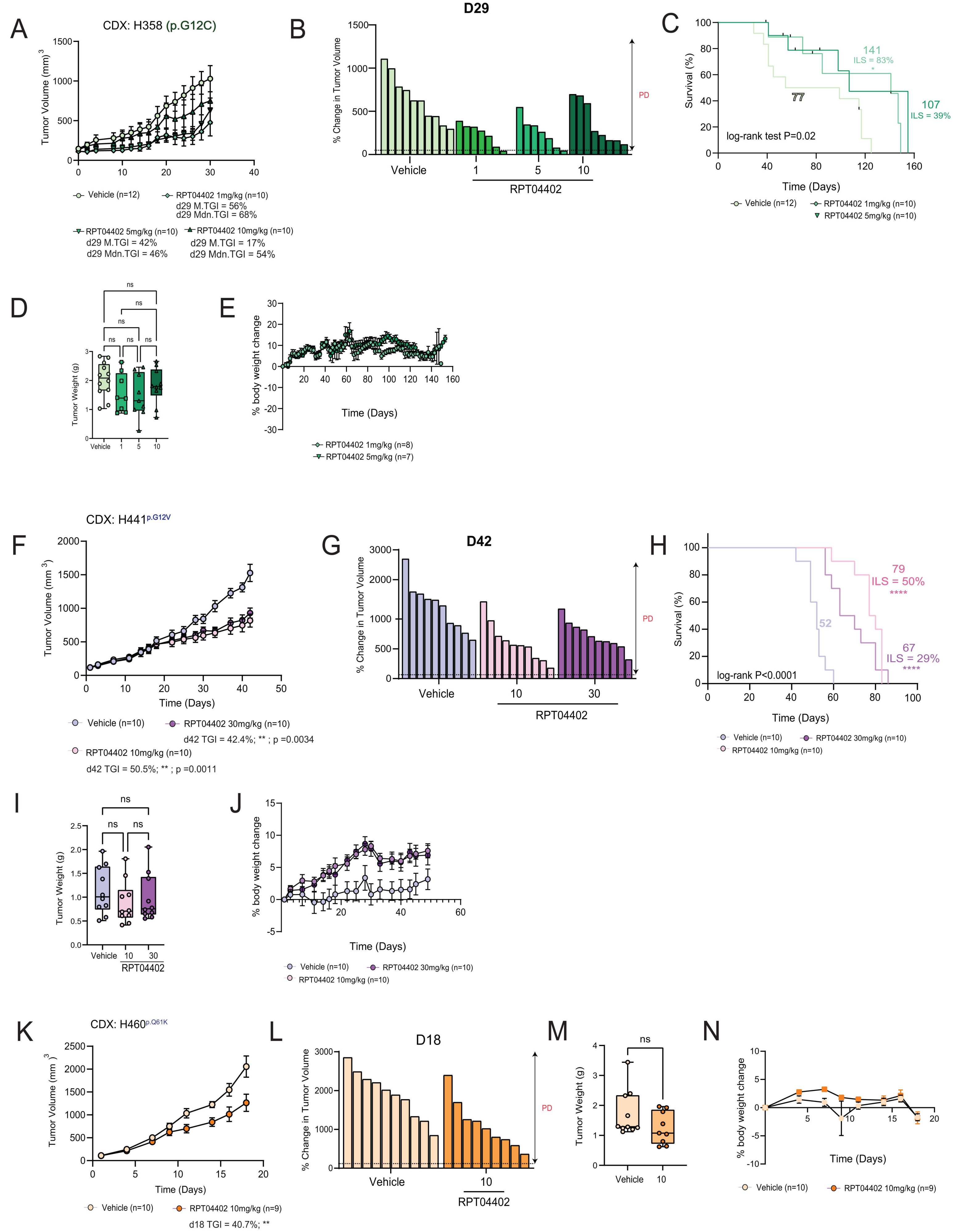

Supplemental Figure 15. RPT04402 promoted tumor growth inhibition in multiple *KRAS*-mutant cell-line derived xenografts.

(**A**) Tumor growth curves for NCI-H358 CDX mice treated with vehicle (n = 10), RPT04402 1 mg/kg QD (n = 8), RPT04402 5 mg/kg QD (n = 7) or, RPT04402 10 mg/kg QD (n = 8). TGI was assessed at the study endpoint for the vehicle groups (36 days). The vehicle and 10mg/kg RPT04402 are from the same cohort as the mice in **Figure 6, D-F**. All statistics were run to consider all groups – raw numbers are in the Supporting Values Data file. (**B**) Waterfall plot displaying the percentage change in tumor volume on day 29 for each treatment arm. The plot indicates responses based on RECIST criteria: PD (progressive disease). (**C**) Kaplan-Meier plot showing survival curves for the vehicle, RPT04402 1 mg/kg, RPT04402 5 mg/kg and, RPT04402 10 mg/kg treatment groups. Median survival (days) and ILS are shown directly on the graph. Statistical significance was determined using the Log-rank (Mantel-Cox) test. (**D**) Graph of tumor weights (grams) from vehicle and RPT04402 (1, 5, and 10 mg/kg) tumors harvested after endpoint. (**E**) Percent body weight change graph of RPT04402 1 and 5 mg/kg treatment groups. (**F**) Tumor growth curves for NCI-H441 CDX mice treated with vehicle (n = 10), RPT04402 10 mg/kg QD (n = 10), and RPT04402 30 mg/kg QD (n = 10). TGI was assessed at the study endpoint for the vehicle groups (42 days). Statistical significance was determined using two-way repeated measures ANOVA with Tukey's multiple comparisons. Only TGI data and statistical significance at the vehicle endpoint are shown on the curve: $^{**}p \leq 0.01$. (**G**) Waterfall plot displaying the percentage change in tumor volume on day 42 for each treatment arm. The plot indicates responses based on RECIST criteria: PD (progressive disease). (**H**) Kaplan-Meier plot showing survival curves for the vehicle, RPT04402 10 mg/kg, and RPT04402 30 mg/kg treatment groups. Median survival (days) and ILS are shown directly on the graph. Statistical significance was determined using the Log-rank (Mantel-Cox) test. (**I**) Graph of tumor weights (grams) from vehicle and RPT04402 (10 and 30 mg/kg) tumors harvested after endpoint. (**J**) Percent body weight change graph of vehicle, RPT04402 10 and 30 mg/kg treatment groups. (**K**) Tumor growth curves for NCI-H460 CDX mice treated with vehicle (n = 10) or RPT04402 10 mg/kg QD (n = 9). TGI was assessed at the study endpoint for the vehicle groups (42 days). Statistical significance was determined using a mixed-effects analysis with Tukey's multiple comparisons test. Only TGI data and statistical significance at the Vehicle endpoint are shown on the curve: $^{**}p \leq 0.01$. (**L**) Waterfall plot displaying the percentage change in tumor volume on day 18 for each treatment arm. The plot indicates responses based on RECIST criteria: PD (progressive disease). (**M**) Graph of tumor weights (grams) from vehicle and RPT04402 30 mg/kg tumors harvested after endpoint. (**N**) Percent body weight change graph of vehicle and RPT04402 10 mg/kg treatment groups.

Supplemental Figure 16

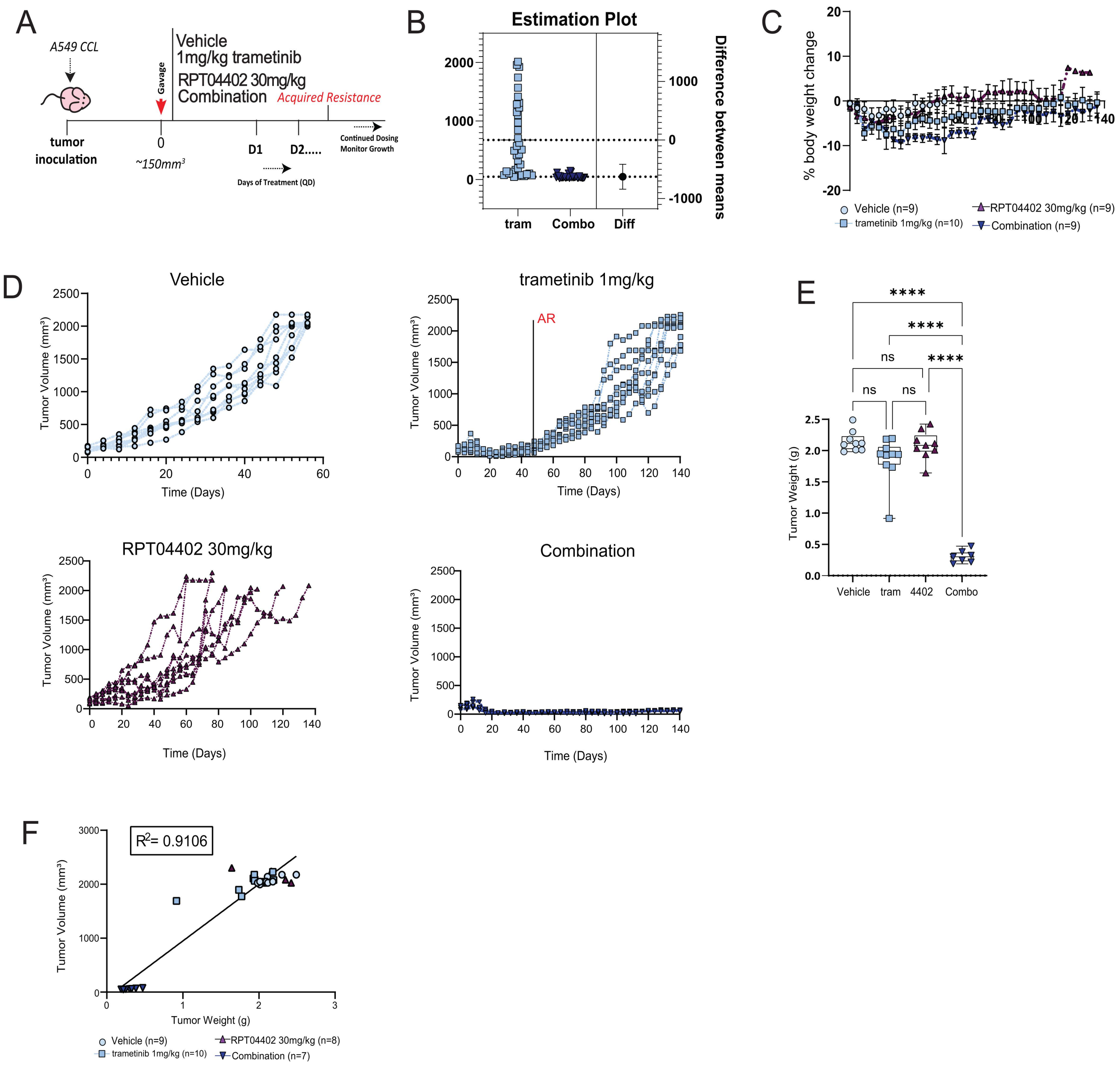

Supplemental Figure 16. RPT04402 synergized with trametinib in vivo.

(**A**) Schematic of the A549 CDX combination study. Mice were treated daily with Vehicle, trametinib (1 mg/kg), RPT04402 (30 mg/kg), or the combination. Mice were removed from treatment when tumors reached the study endpoint of ~ 2000mm^3. (**B**) Estimation plot comparing tumor volume means between the trametinib and combination treatment arms. (**C**) Percent body weight change of indicated groups for the indicated time points. (**D**) Tumor growth curves of individual mice for each treatment arm. "AR" = onset of acquired resistance. (**E**) Graph of tumor weights (grams) from vehicle, trametinib, RPT04402, and combination treatment arms. Statistical significance was determined by one-way ANOVA with Tukey's post-hoc analysis: ****$p \leq 0.0001$. (**F**) Correlation plot between tumor volume (mm^3) and tumor weights from each of the harvested tumors at the study endpoint.

Supplemental Figure 17

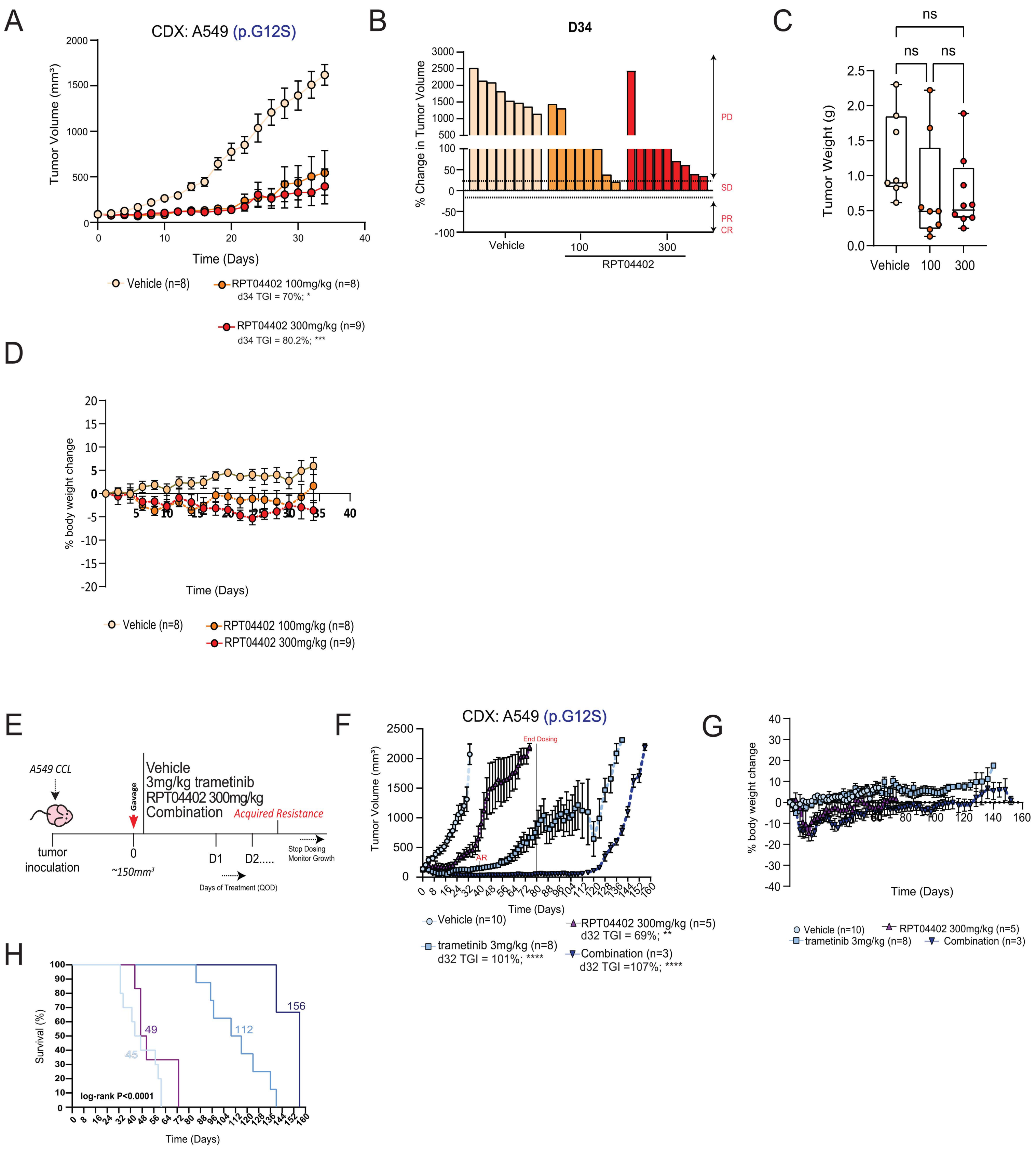

	0	40	60	80	100	140	160
Vehicle	10(0)	7(0)	2(0)	0(0)	0(0)	0(0)	0(0)
Tram	10(0)	8(2)	8(2)	8(2)	5(2)	1(2)	0(2)
RPT04402	9(0)	6(3)	2(3)	2(3)	0(3)	0(3)	0(3)
Combo	9(0)	3(6)	3(6)	3(6)	3(6)	3(6)	3(6)

Supplemental Figure 17. Dose efficacy study and pilot combination of RPT04402 in A549 CDX. (**A**) Tumor growth curves for A549 CDX mice treated with vehicle (n = 8), RPT04402 100 mg/kg QOD (n = 8), or RPT04402 300 mg/kg QOD (n = 9). TGI was assessed at the study endpoint for the vehicle groups (34 days). Statistical significance was determined using two-way repeated measures ANOVA with Tukey's multiple comparisons. Only TGI data and statistical significance at the vehicle endpoint are shown on the curve: ****$p \leq 0.0001$. (**B**) Waterfall plot displaying the percentage change in tumor volume on day 34 for each treatment arm. The plot indicates responses based on RECIST criteria: PD (progressive disease), SD (stable disease), PR (partial response), and CR (complete response). (**C**) Graph of tumor weights from vehicle and RPT04402 (100 and 300 mg/kg) tumors harvested after endpoint. (**D**) Percent body weight change graph of RPT04402 100 and 300 mg/kg treatment groups. (**E**) Schematic of the pilot A549 CDX combination study. Mice were treated every other day (QOD) with vehicle, trametinib (3 mg/kg), RPT04402 (300 mg/kg), or the combination. Mice were removed from treatment when tumors reached the study endpoint of ~ 2000-2500 mm^3. (**F**) Tumor growth curves for A549 CDX. Mice were treated with vehicle, trametinib, RPT04402, or the combination QOD for 80 days before stopping treatment. Mice were continuously monitored until the tumors regrew and reached the study endpoint. Tumor growth inhibition and statistical significance on the graph represent day 32 – when the final vehicle mouse reached its tumor volume endpoint. Statistical significance was determined using two-way repeated measures ANOVA with Tukey's multiple comparisons. (**G**) Percent body weight change of indicated groups for the indicated time points. (**H**) Kaplan-Meier plot showing survival curves for the vehicle, trametinib, RPT04402, and combination. Median survival (days) is shown directly in the graph. Statistical significance was determined using the Log-rank (Mantel-Cox) test.

SupplementalFigure 18

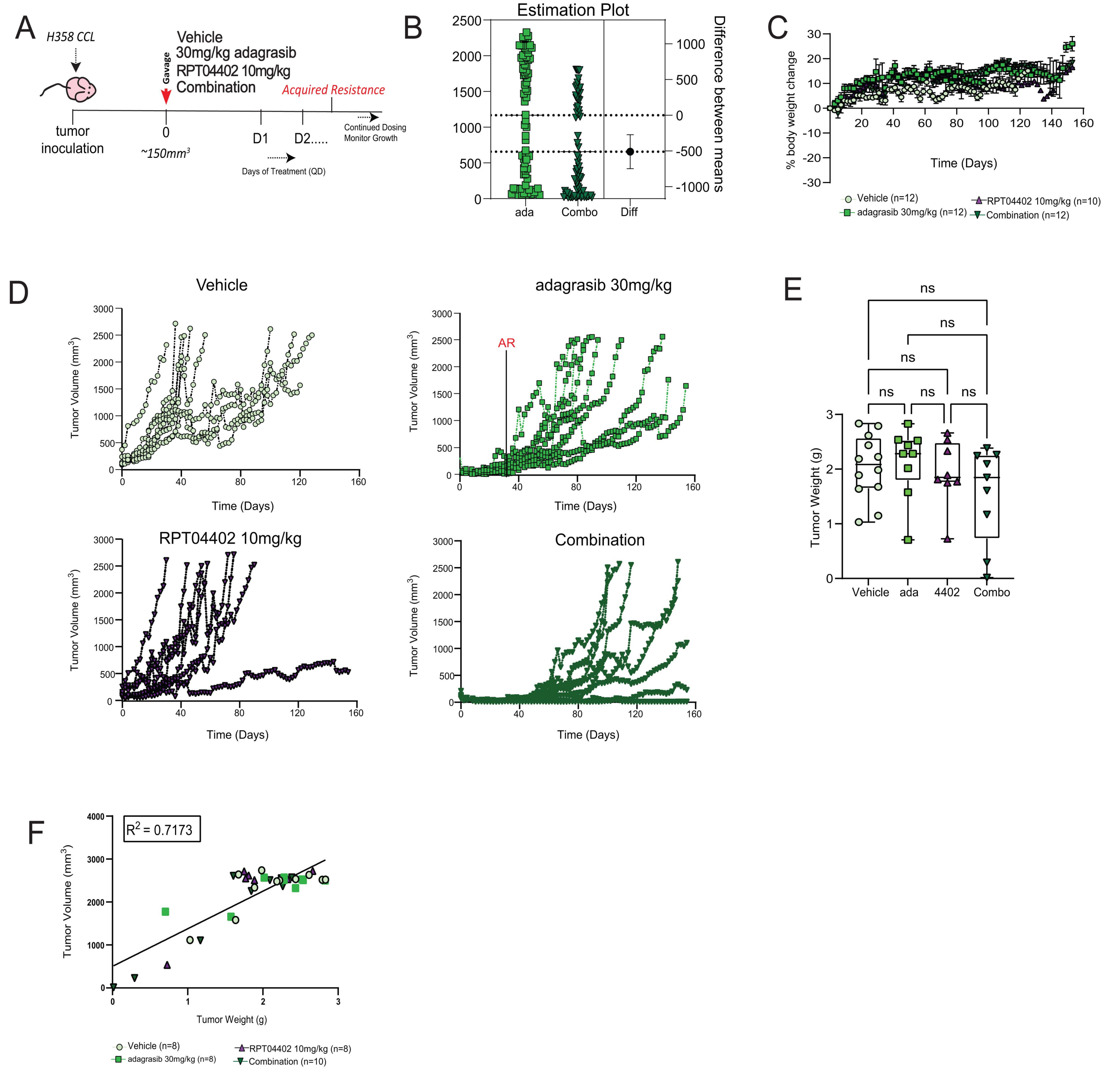

Supplemental Figure 18. RPT04402 potentiated adagrasib in vivo.
(**A**) Schematic of the NCI-H358 CDX combination study. Mice were treated daily with vehicle, adagrasib (30 mg/kg), RPT04402 (10 mg/kg), or the combination. Mice were removed from treatment when tumors reached the study endpoint of ~ 2000mm^3. (**B**) Estimation plot comparing tumor volume means between the adagrasib and combination treatment arms. (**C**) Percent body weight change of indicated groups for the indicated time points. (**D**) Tumor growth curves of individual mice for each treatment arm. "AR" = onset of acquired resistance (**E**) Graph of tumor weights (grams) from vehicle, adagrasib, RPT04402, and combination treatment arms. Statistical significance was determined by one-way ANOVA with Tukey's post-hoc analysis: ****$p \leq 0.0001$. (**F**) Correlation plot between tumor volume (mm^3) and tumor weights from each of the harvested tumors at the study endpoint.

Supplemental Figure 19

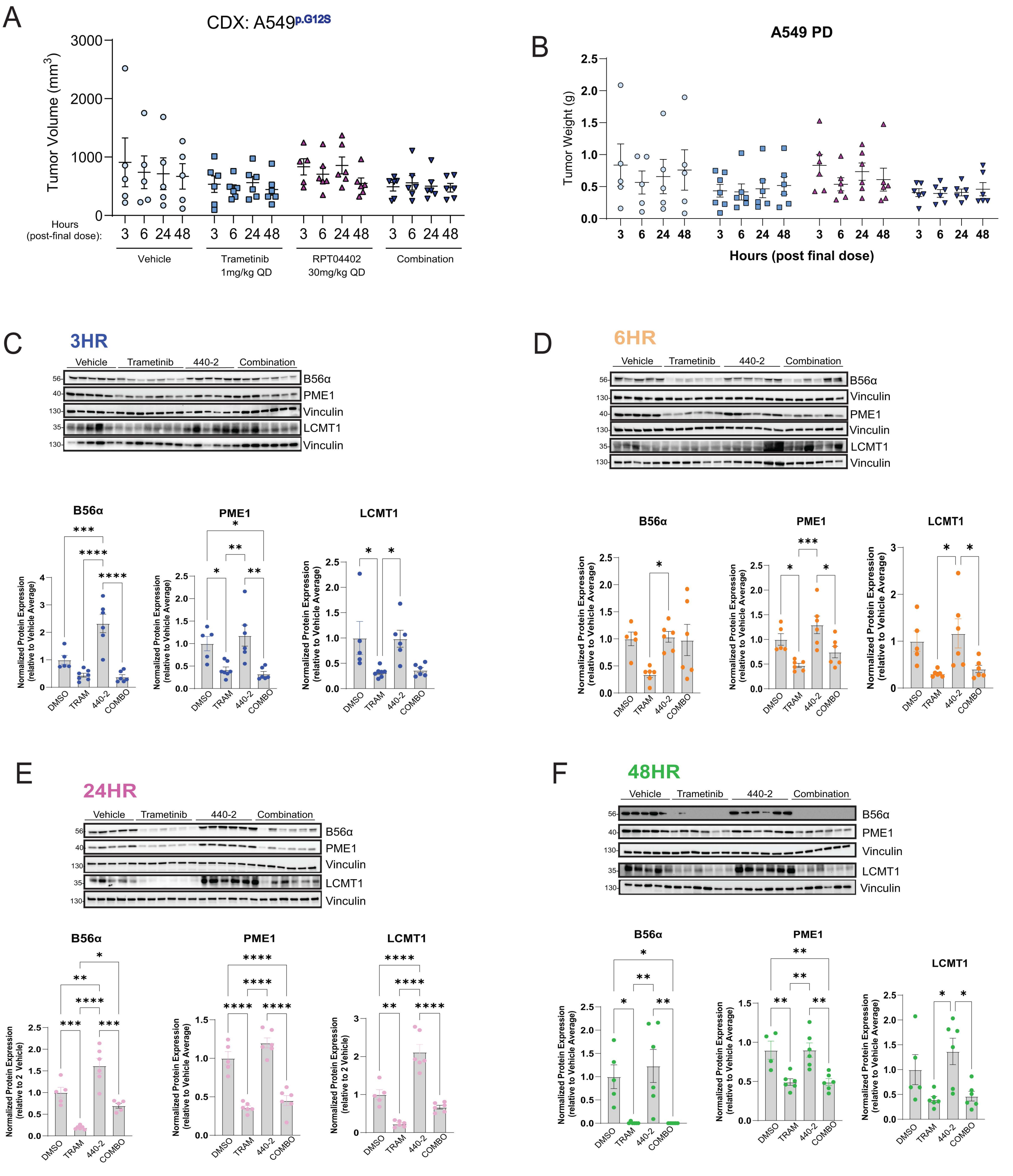

Supplemental Figure 19. MEK1/2 inhibition and RPT04402 synergized to rescue B56*α* expression in vivo.

(**A**) Separated scatter plot of tumor volume (mm^3) at the study endpoint of the A549 CDX PD study. Mice were treated daily for 7 days with vehicle, trametinib (1 mg/kg), RPT04402 (30 mg/kg), or the combination. Mice were euthanized 3, 6, 24, or 48 hours after the final dose for analysis. (**B**) Separated scatter plot of tumor weights from each timepoint and treatment group. Statistical significance for the tumor volume and weights was determined by mixed-effects analysis using Tukey's multiple comparisons test.(**C**) Western blot and quantification of B56α, PME1, and LCMT1 from A549 PD tumor lysates 3 hours post-final dose. (**D**) Western blot and quantification of the same target proteins 6 hours post-final dose. (**E**) Western blot and quantification of the same target proteins 24 hours post-final dose. (**F**) Western blot and quantification of the same target proteins 48 hours post-final dose. All target proteins were normalized to the "Vinculin" loading control. Densitometry data is expressed relative to the average of the "vehicle" treatment group. Statistical significance was determined by one-way ANOVA using Tukey's post-hoc analysis. Only significant results from the post-hoc analysis are shown: *p≤0.5, **p≤0.01, ***p≤0.001. Raw data and analyses are in the Supporting Data Values file.

Supplemental Figure 20

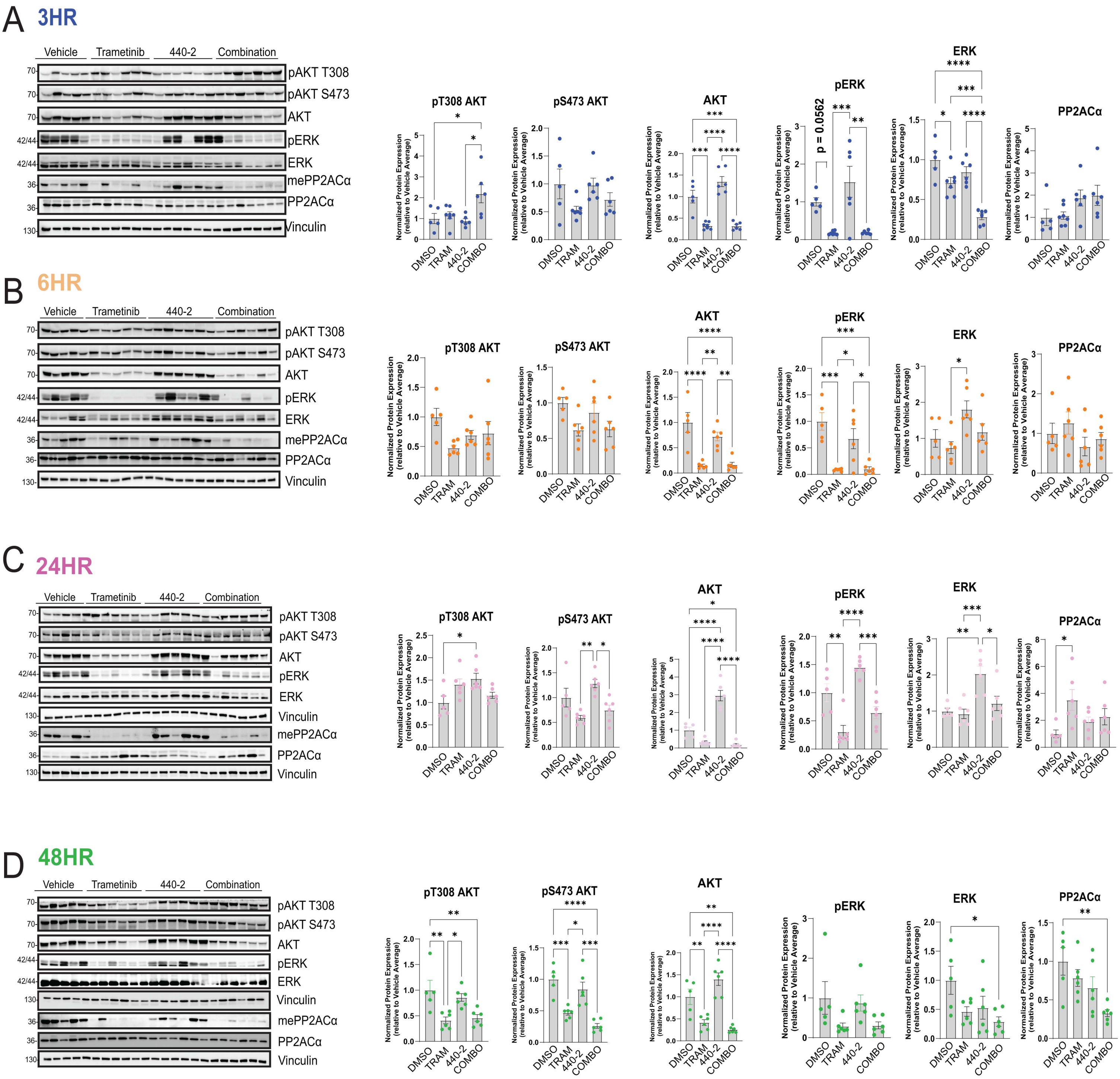

Supplemental Figure 20. Effects of MEK1/2 inhibition and RPT04402 on ERK and AKT signaling in vivo.

(**A**) Western blot analysis and quantification of pERK, pAKT (pT308 and pS473), mePP2ACα, and PP2ACα in tumors from A549 CDX PD treated with vehicle, trametinib (1 mg/kg), RPT04402 (30 mg/kg), and the combination harvested 3 hours post final dose. (**B**) Western blot and quantification of the same target proteins 6 hours post-final dose. (**C**) Western blot and quantification of the same target proteins 24 hours post-final dose. (**D**) Western blot and quantification of the same target proteins 48 hours post-final dose. All target proteins were normalized to the "Vinculin" loading control and the specific loading control blots are indicated on the figures. Notably, some vinculin blots are duplicated between the figures as some targets where run on the same blot. This is indicated in the figure. Densitometry data is expressed relative to the average of the "vehicle" treatment group. All data and analyses, including quantification for tERK and tAKT normalized to the loading control, are in the Supporting Data Values file. Statistical significance was determined by one-way ANOVA using Tukey's post-hoc analysis. Only significant results from the post-hoc analysis are shown: *$p \leq 0.5$, **$p \leq 0.01$, ***$p \leq 0.001$. Note, mePP2ACα blots appear twice in Figure 7 and here.

Supplemental Figure 21

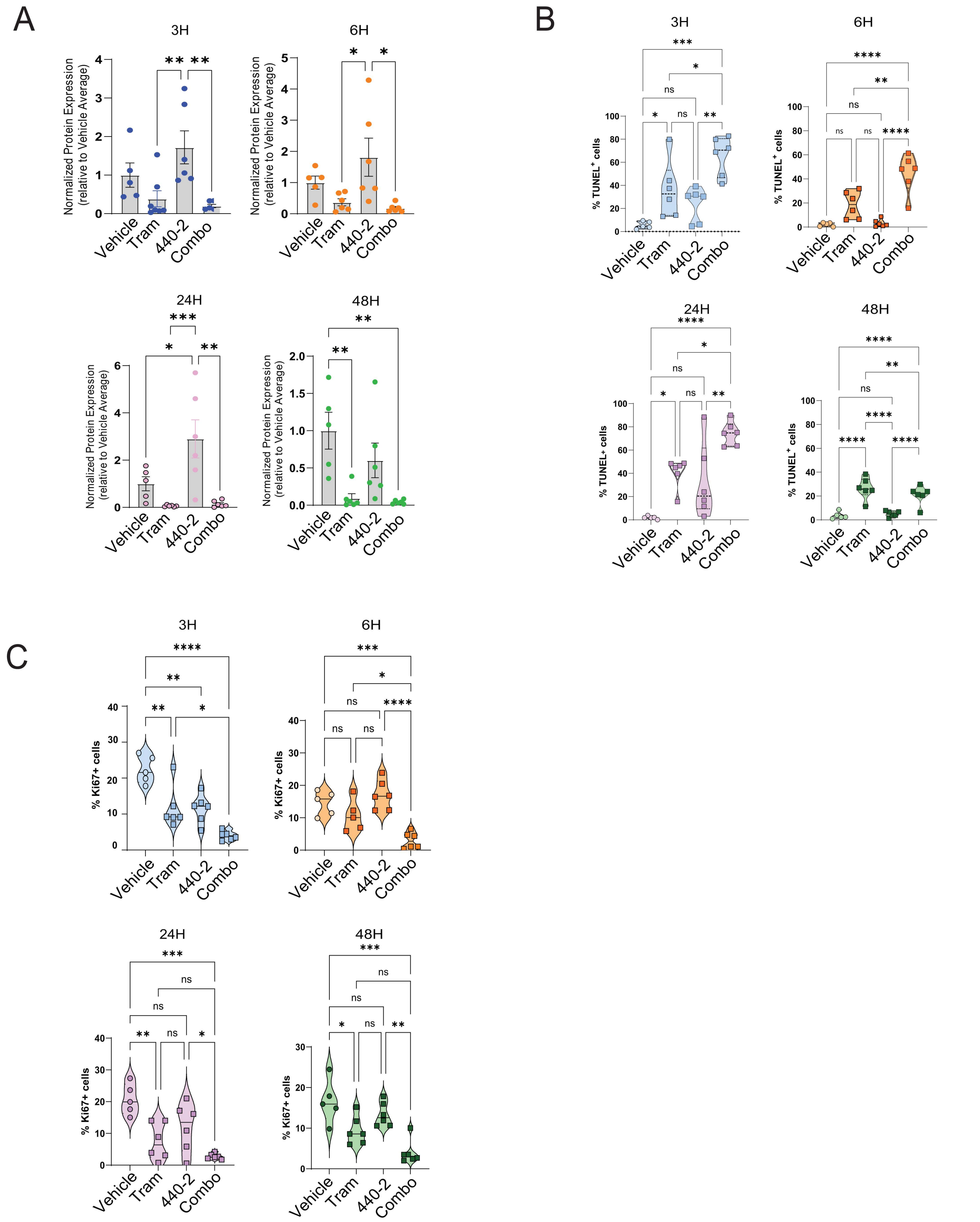

Supplemental Figure 21. Quantification of pharmacodynamic (PD) markers in A549 CDX tumors from Figure 7.

(**A**) Quantification of mePP2ACα levels from western blots shown in Figure 7C. (**B**) Quantification of TUNEL IHC staining at 3, 6, 24, 48 hours after the final dose. (**C**) Quantification of Ki67 IHC staining at the same time points. Statistical significance for each time point was determined using One-way ANOVA with Tukey's post-hoc analysis: *p≤0.5, **p≤0.01, ***p≤0.001, ****p≤0.0001.
