# Supplementary material for: A PP2A molecular glue overcomes RAS/MAPK inhibitor resistance in KRAS-mutant non–small cell lung cancer
Source: J Clin Invest. 2025 Oct 14;135(23):e193790. doi: 10.1172/JCI193790 (PMC12646674; doi:10.1172/JCI193790)
Supplement: Unedited blot and gel images [file jci-135-193790-s078.pdf]

Uncropped Western Blots

Raines B, et al. A PP2A Molecular Glue Overcomes RAS/MAPK Inhibitor Resistance in *KRAS*-mutant Non-Small Cell Lung Cancer

Full unedited blot/gel for Supplemental Figure 1A

Red boxes indicate the images used in Supplemental Figure 1A.

A549

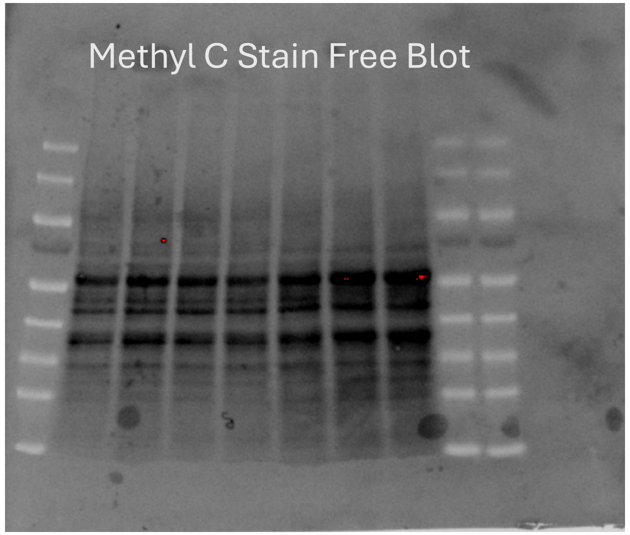

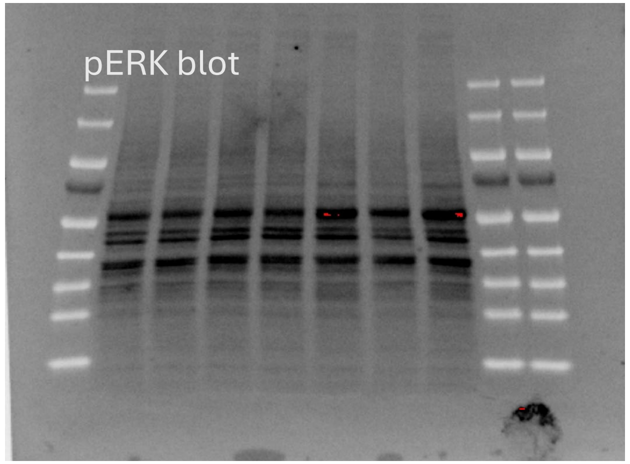

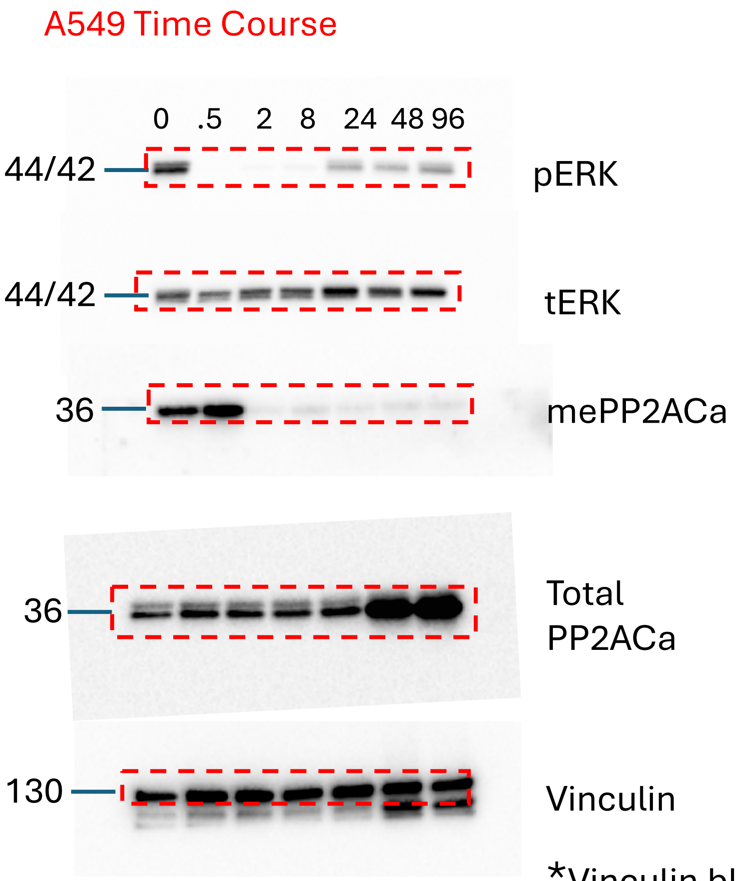

*Vinculin blot is in Supplemental Figure 2A

Full unedited blot/gel for Supplemental Figure 1B

Red boxes indicate the images used in Supplemental Figure 1B.

A549

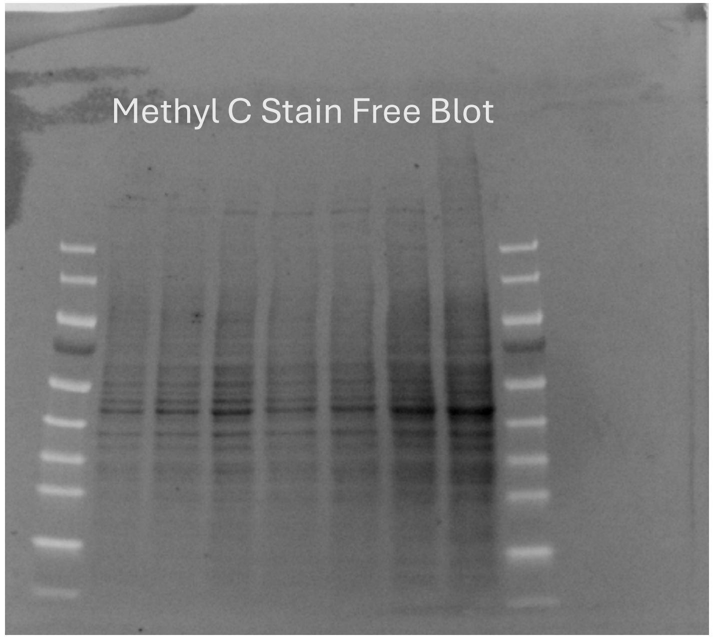

H358 Time Course

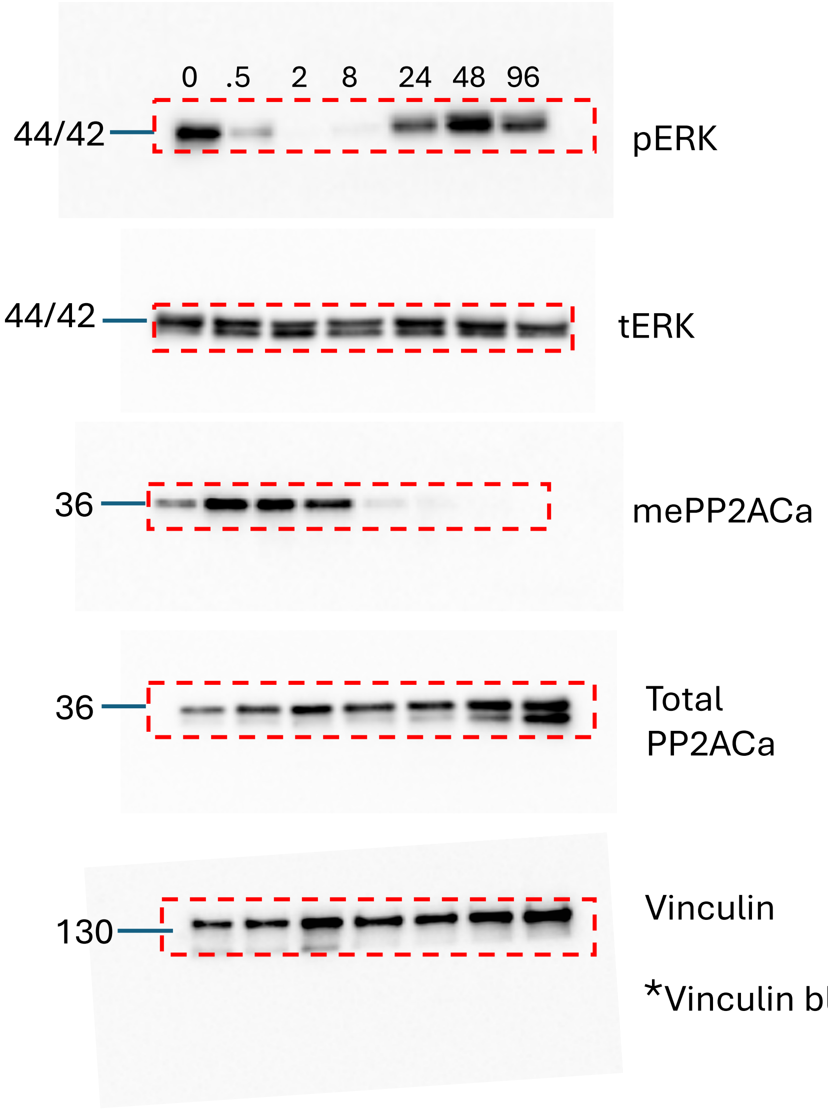

*Vinculin blot is in Supplemental Figure 2D

Full unedited blot/gel for Supplemental Figure 1, E and F

Red boxes indicate the images used in Supplemental Figure 1, E and F.

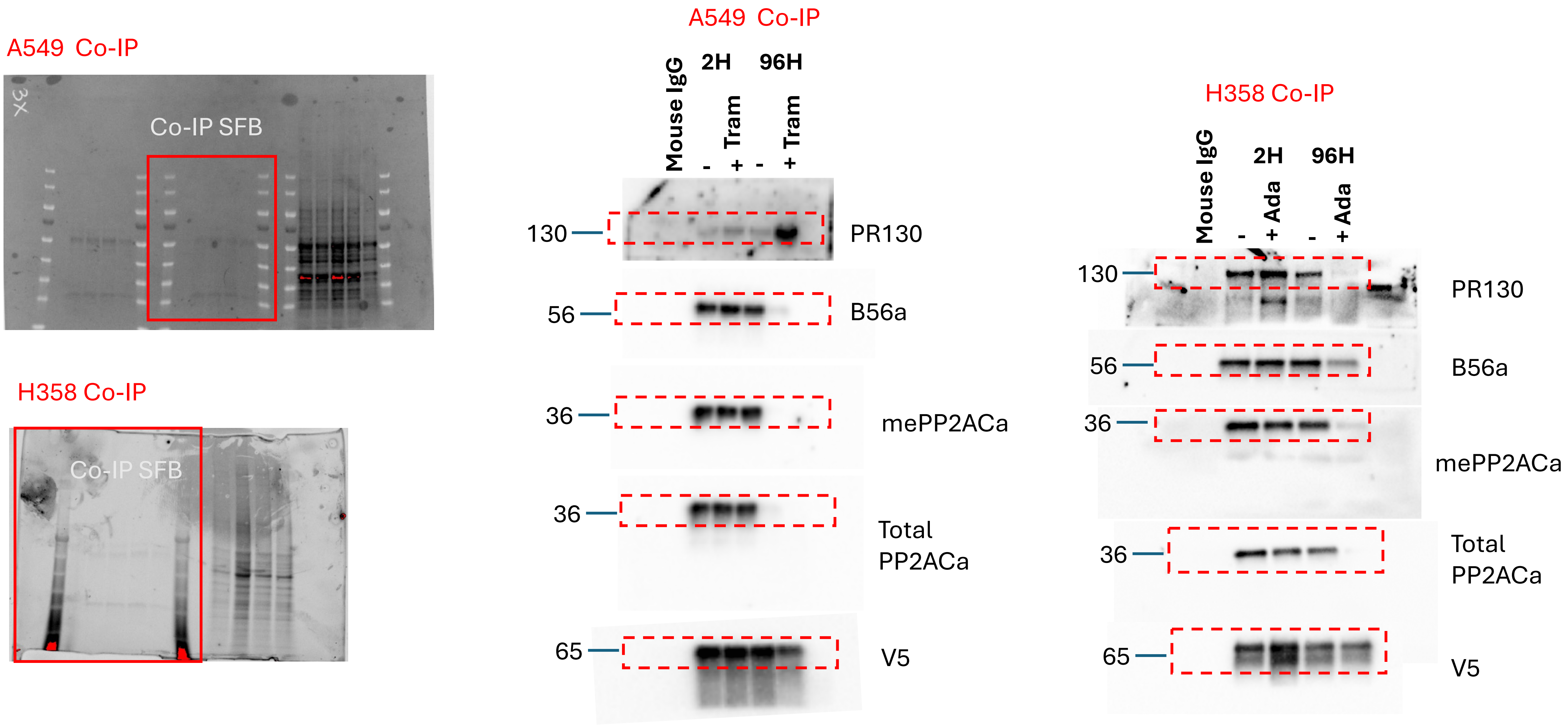

Full unedited blot/gel for Supplemental Figure 2, A and B, E

Red boxes indicate the images used in Supplemental Figure 2, A and B, E.

A549

Representative Western

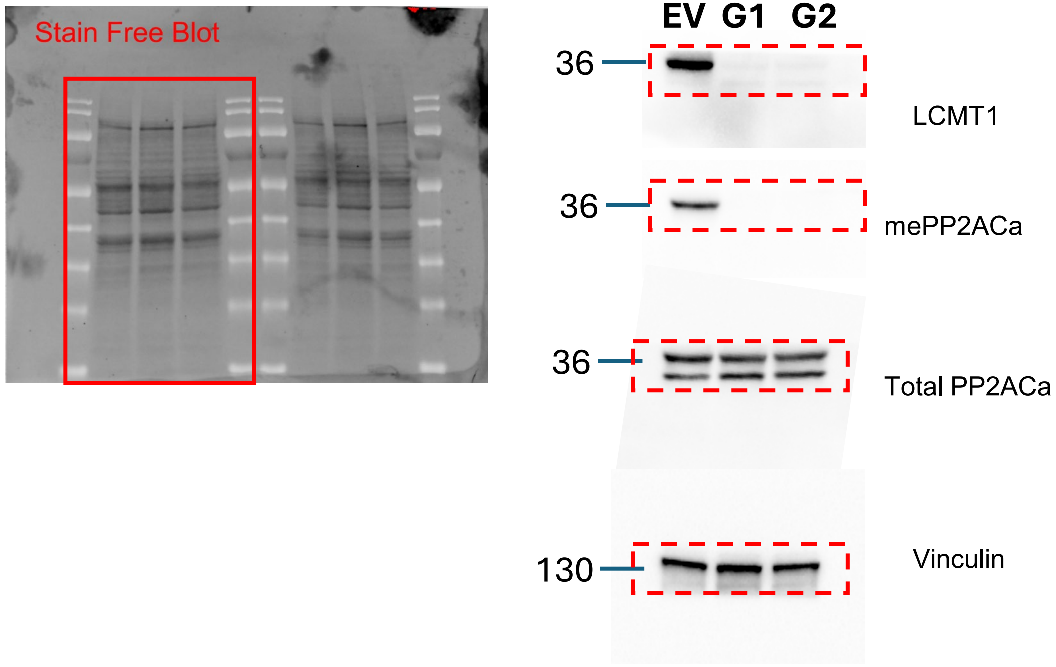

H358

Representative Western

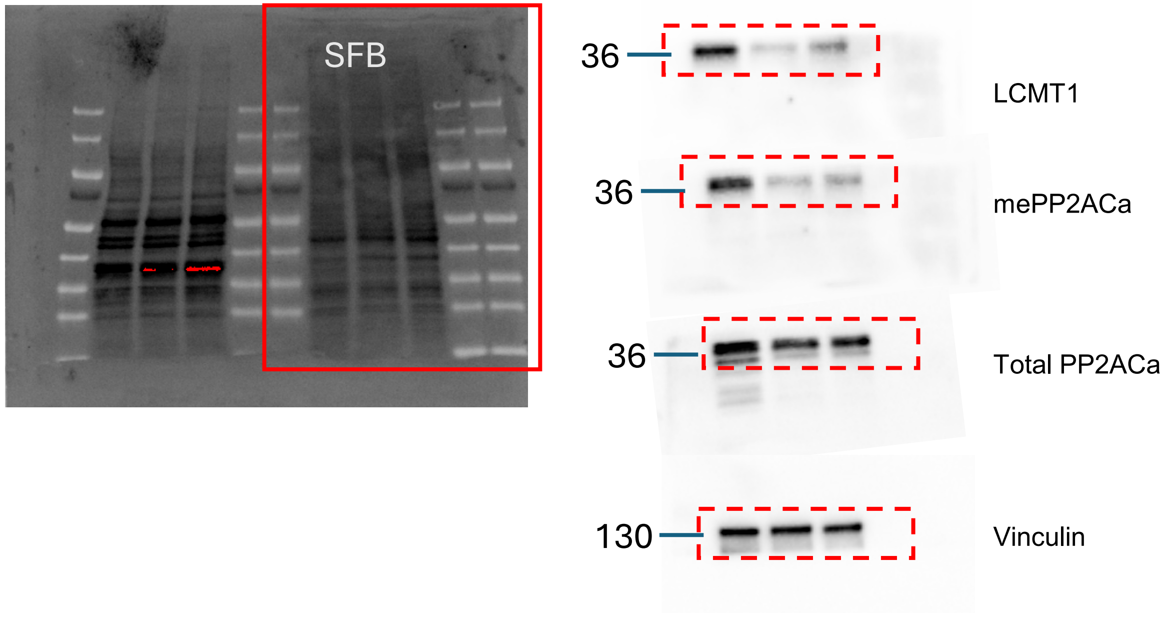

Full unedited blot/gel for Supplemental Figure 2, A and B, E continued

Red boxes indicate the images used in Supplemental Figure 2, A and B, E.

A549

Representative Western, 2E

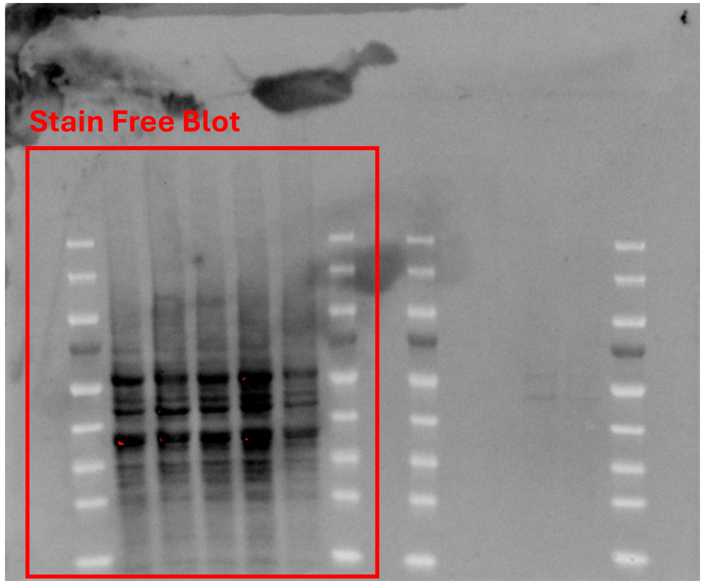

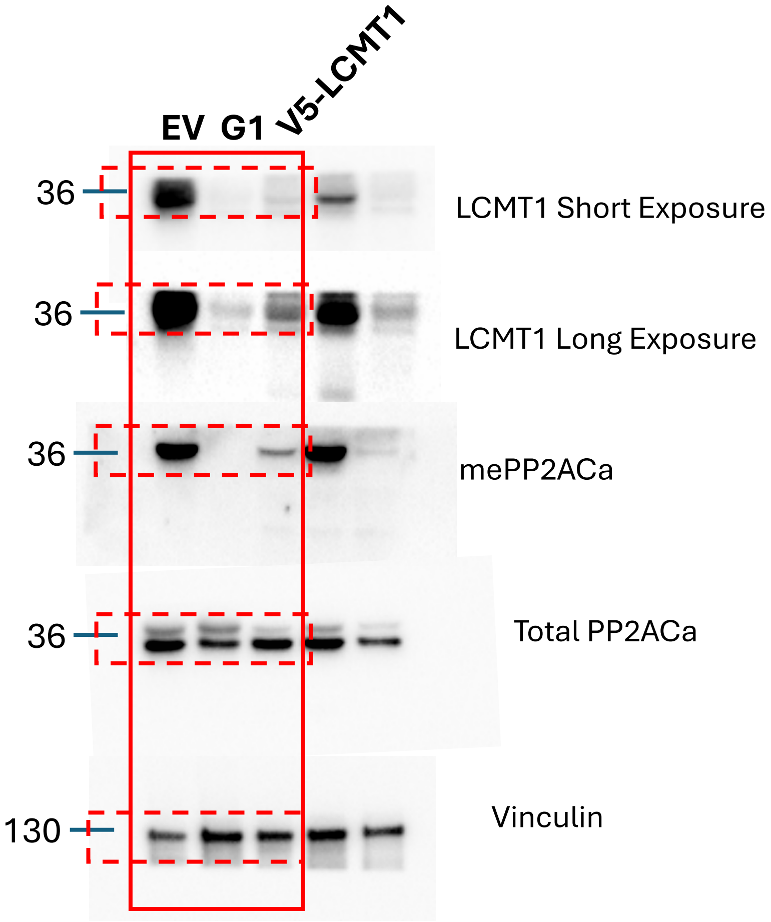

Full unedited blot/gel for Supplemental Figure 3F

Red boxes indicate the images used in Supplemental Figure 3F.

A549

Representative Western, 3F

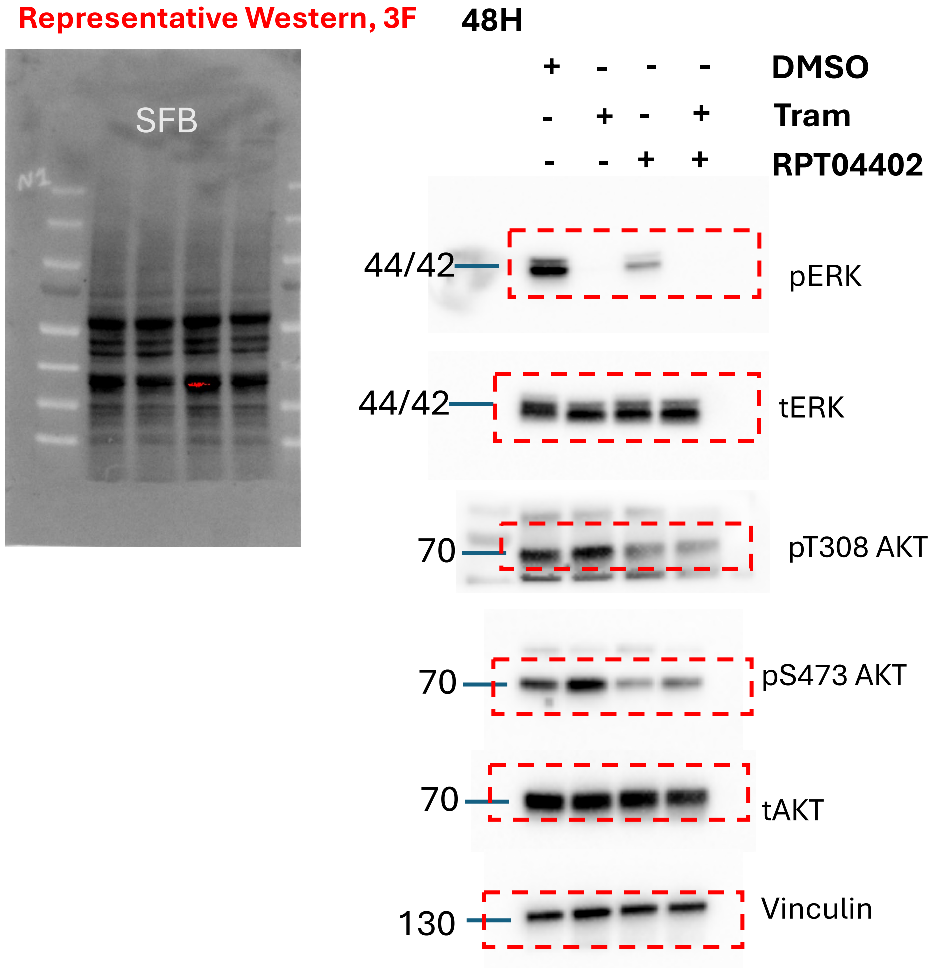

6652 CL

Representative Western, 3F

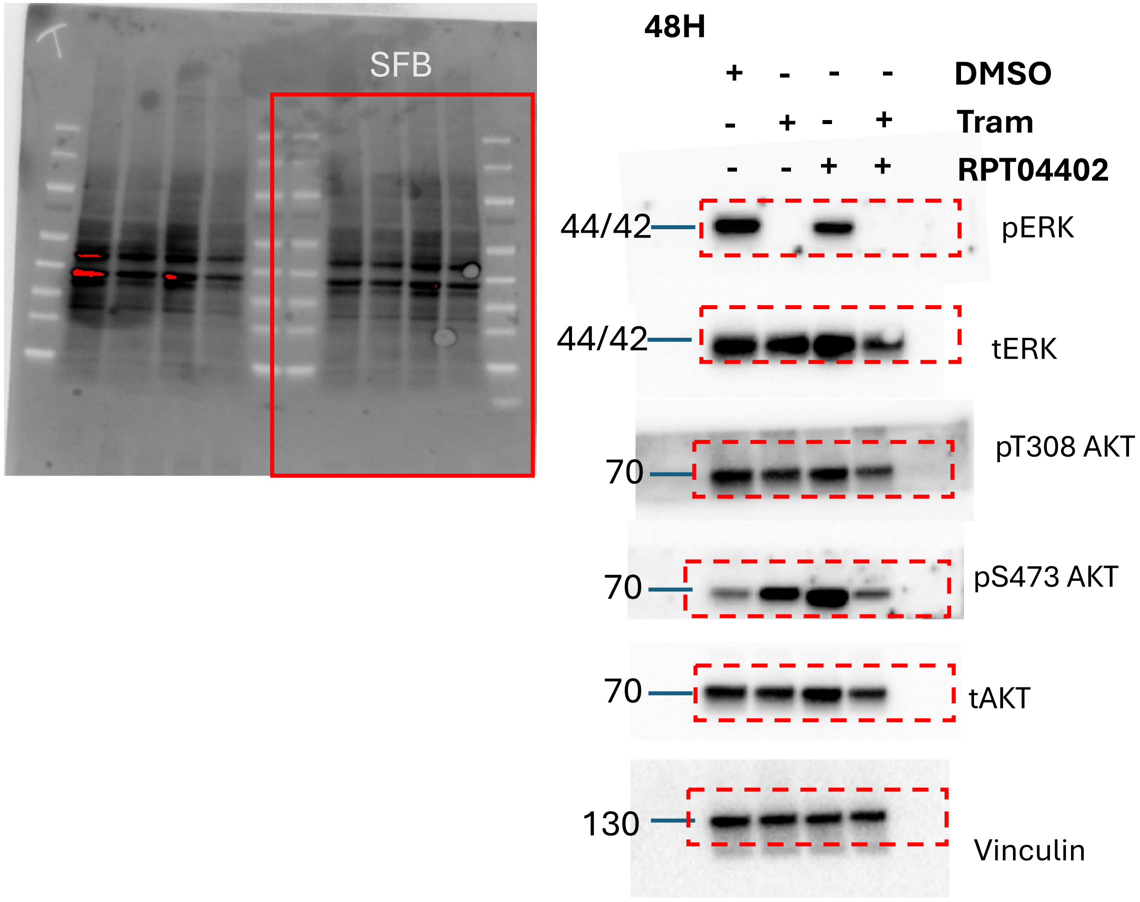

Full unedited blot/gel for Supplemental Figure4

Red boxes indicate the images used in Supplemental Figure 4F.

H358

Representative Western, 4F

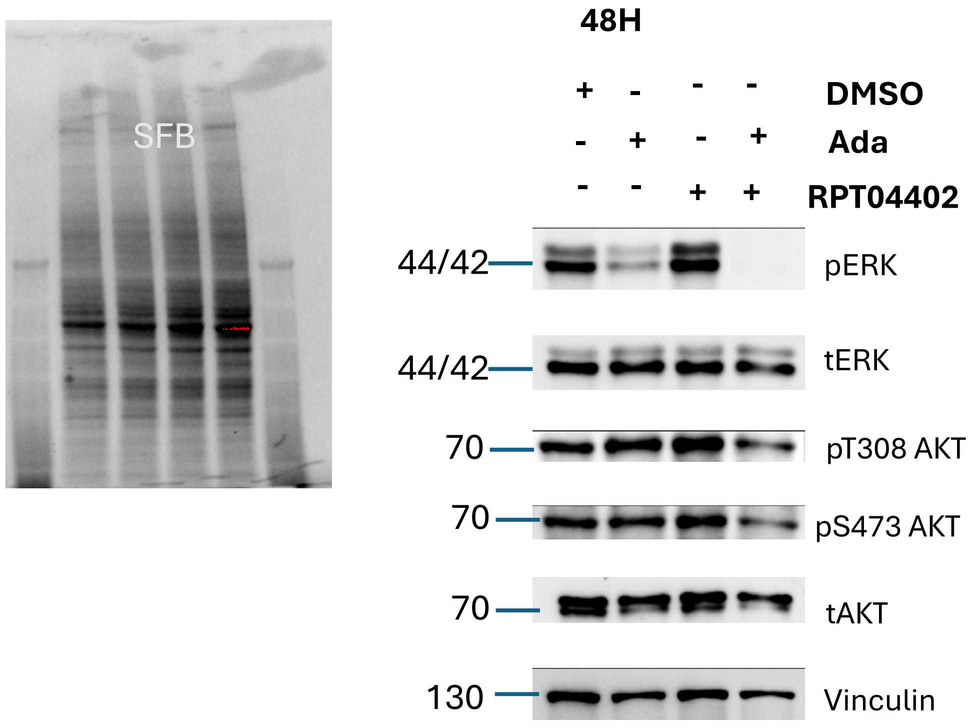

6652 CL

Representative Western, 4F

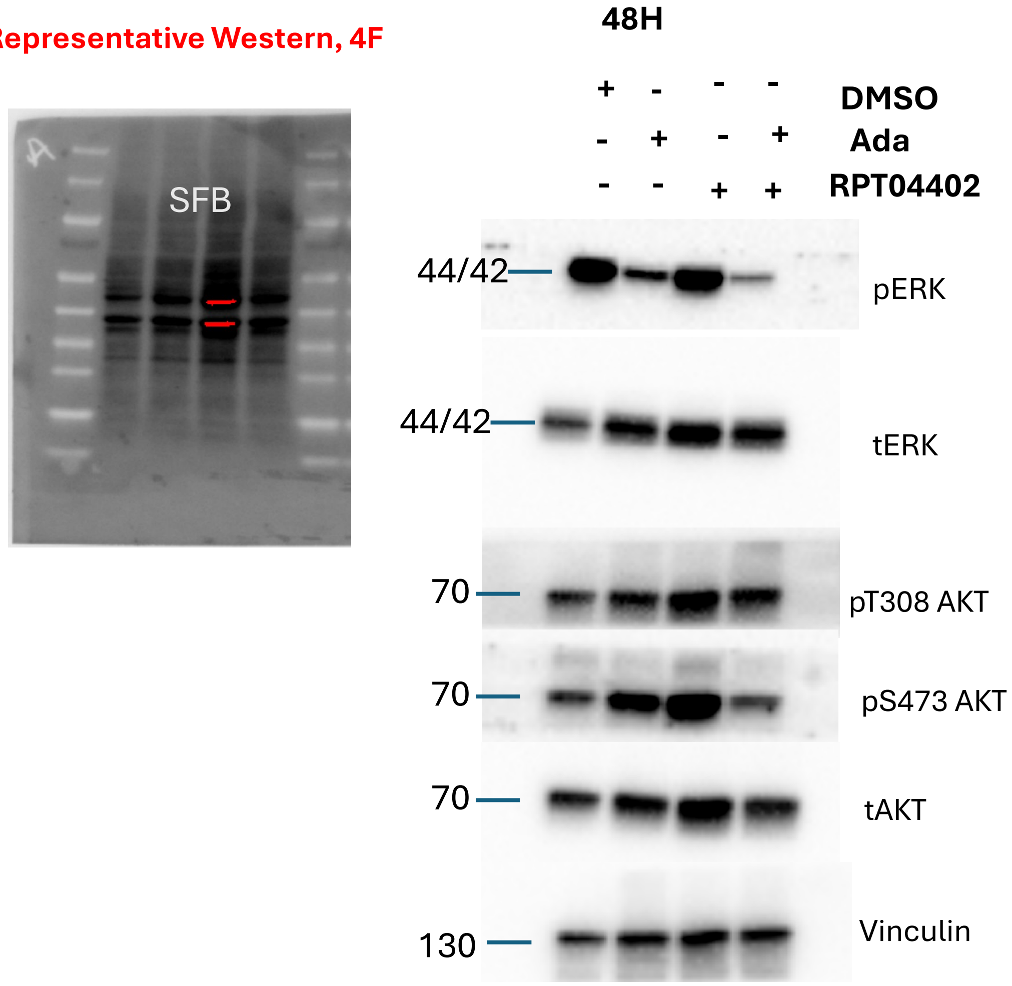

Full unedited blot/gel for Supplemental Figure 5E

Red boxes indicate the images used in Supplemental Figure 5E.

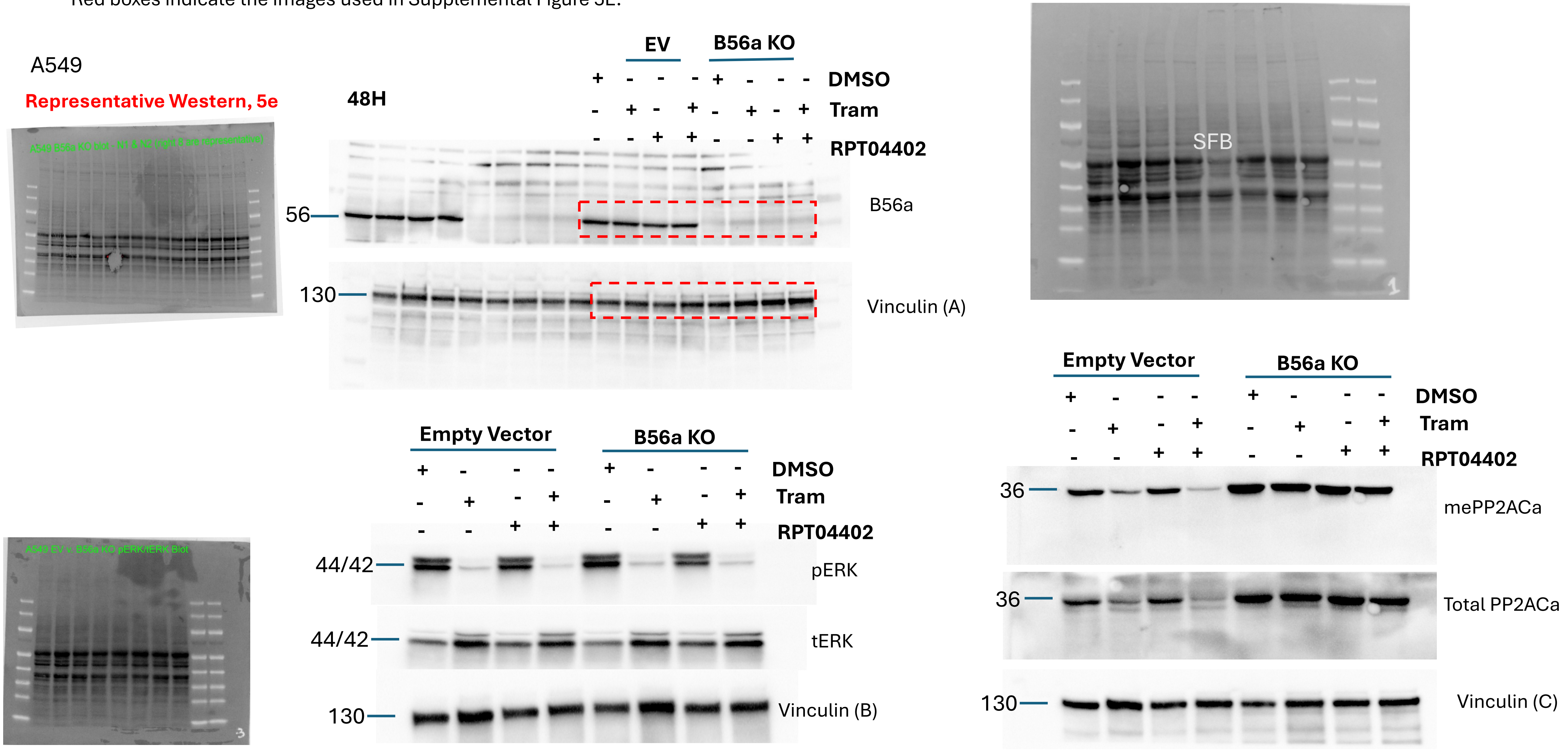

All vinculin blots correspond to Supplemental Figure 13D

Full unedited blot/gel for Supplemental Figure 5F

Red boxes indicate the images used in Supplemental Figure 5F.

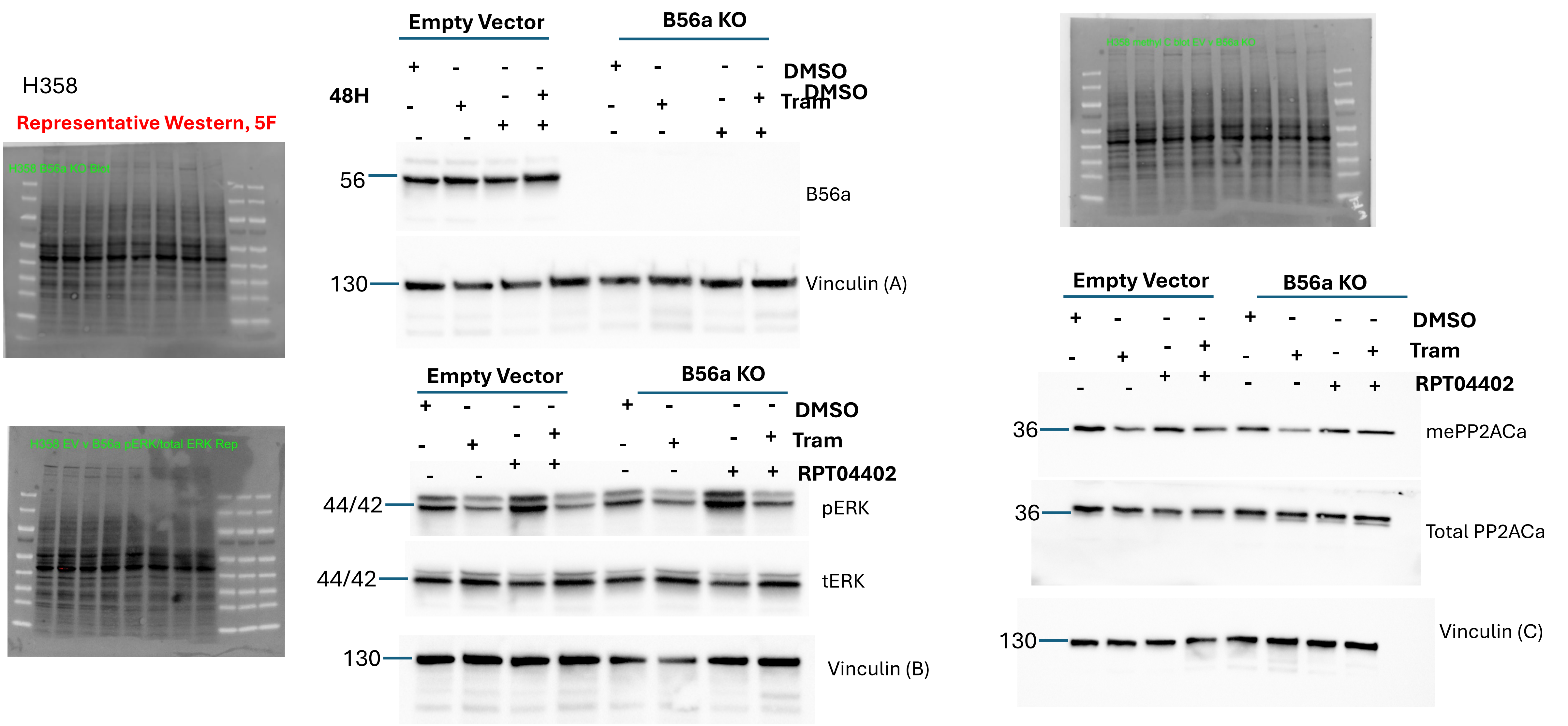

All vinculin blots correspond to Supplemental Figure 13E

Full unedited blot/gel for Supplemental Figure 1, A & B

Red boxes indicate the images used in Supplemental Figure 1, A & B.

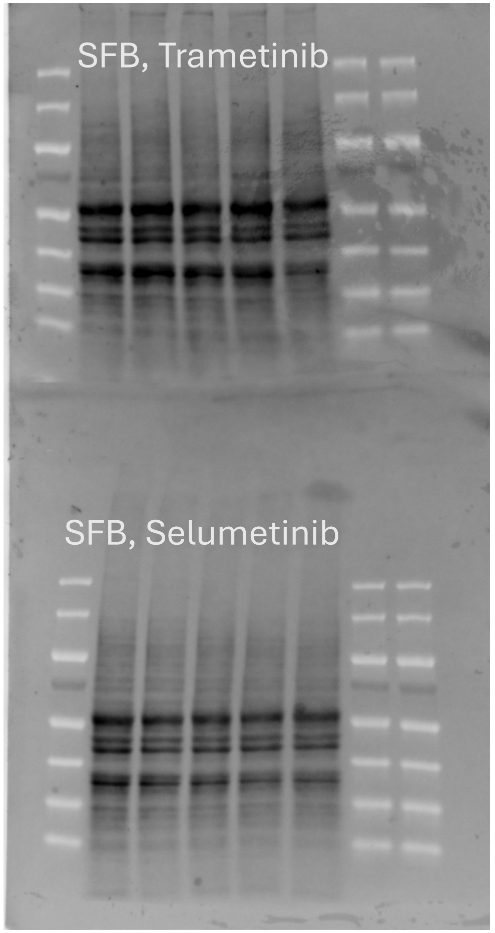

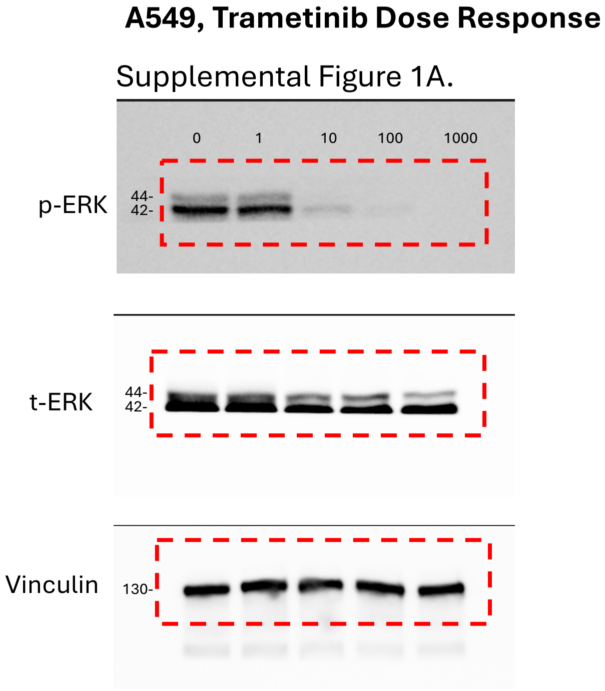

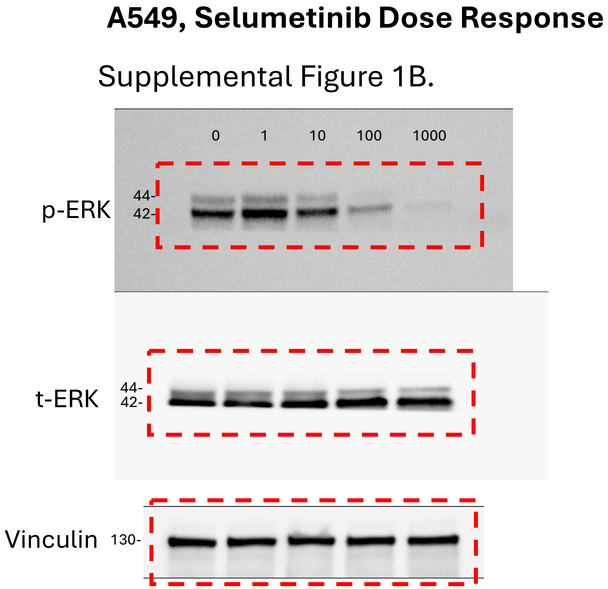

Full unedited blot/gel for Supplemental Figure 1, C & D

Red boxes indicate the images used in Supplemental Figure 1, C & D.

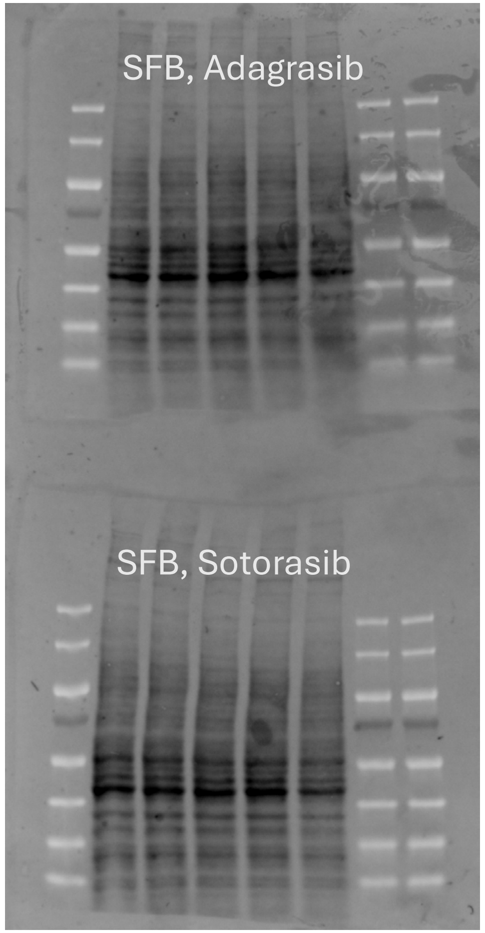

H358, Adagrasib Dose Response

Supplemental Figure 1C.

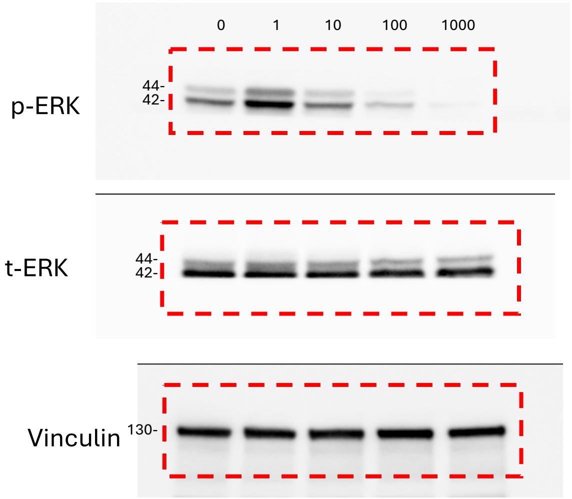

H358, Sotorasib Dose Response

Supplemental Figure 1D.

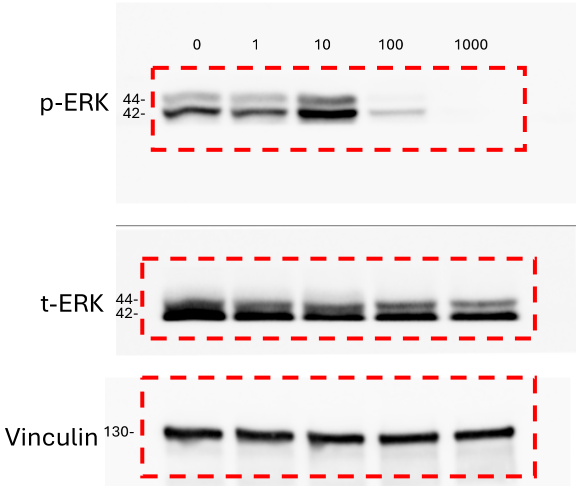

Full unedited blot/gel for Supplemental Figure 2

Red boxes indicate the images used in Supplemental Figure 2.

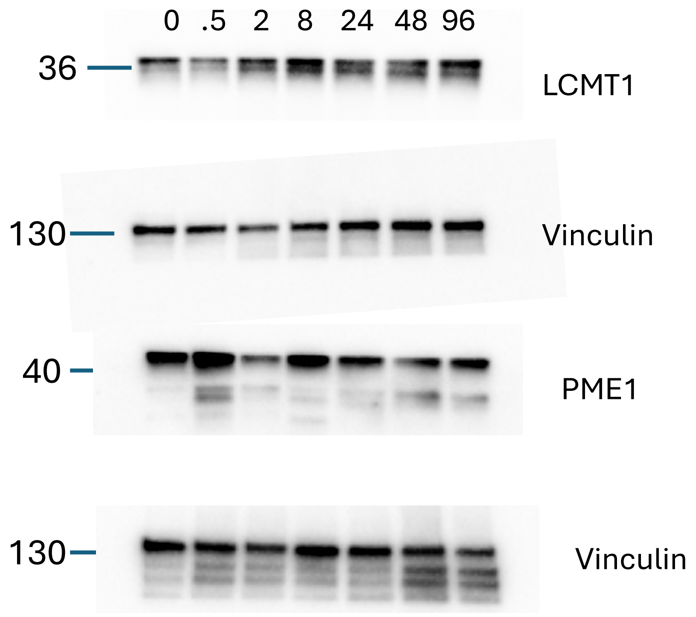

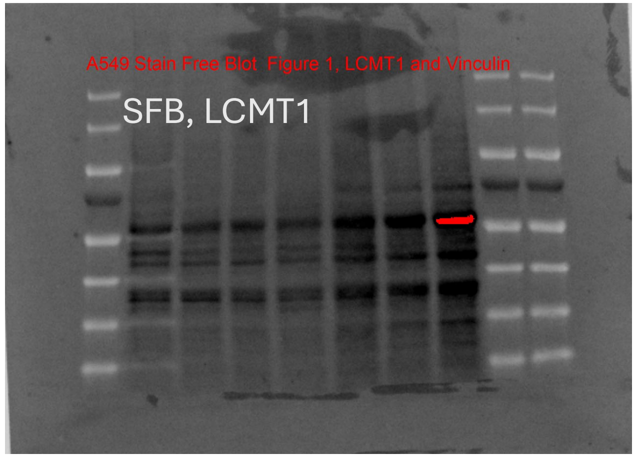

*No sfb for pme1 blot

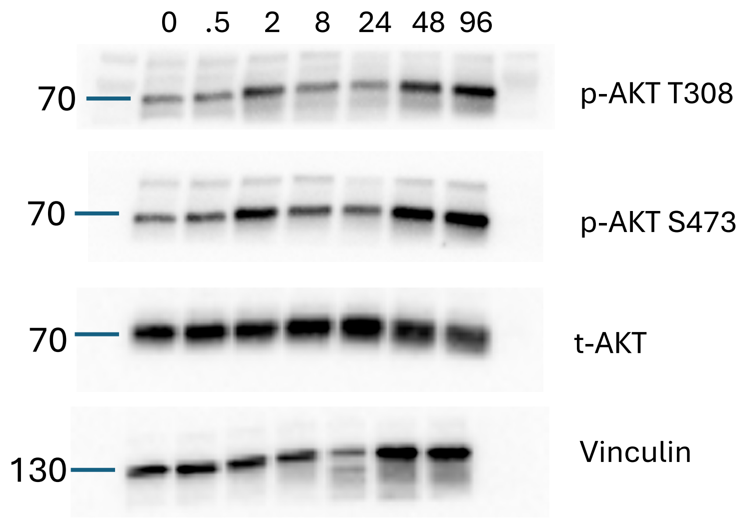

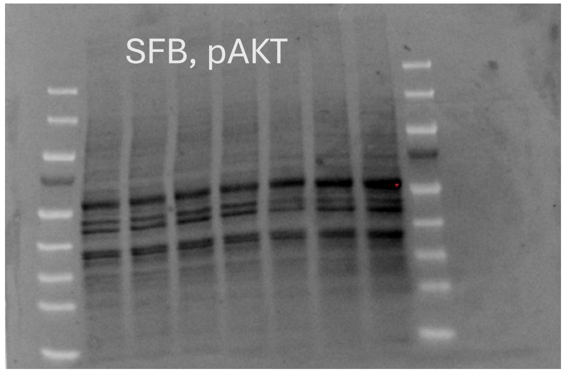

Full unedited blot/gel for Supplemental Figure 3A

Red boxes indicate the images used in Supplemental Figure 3A.

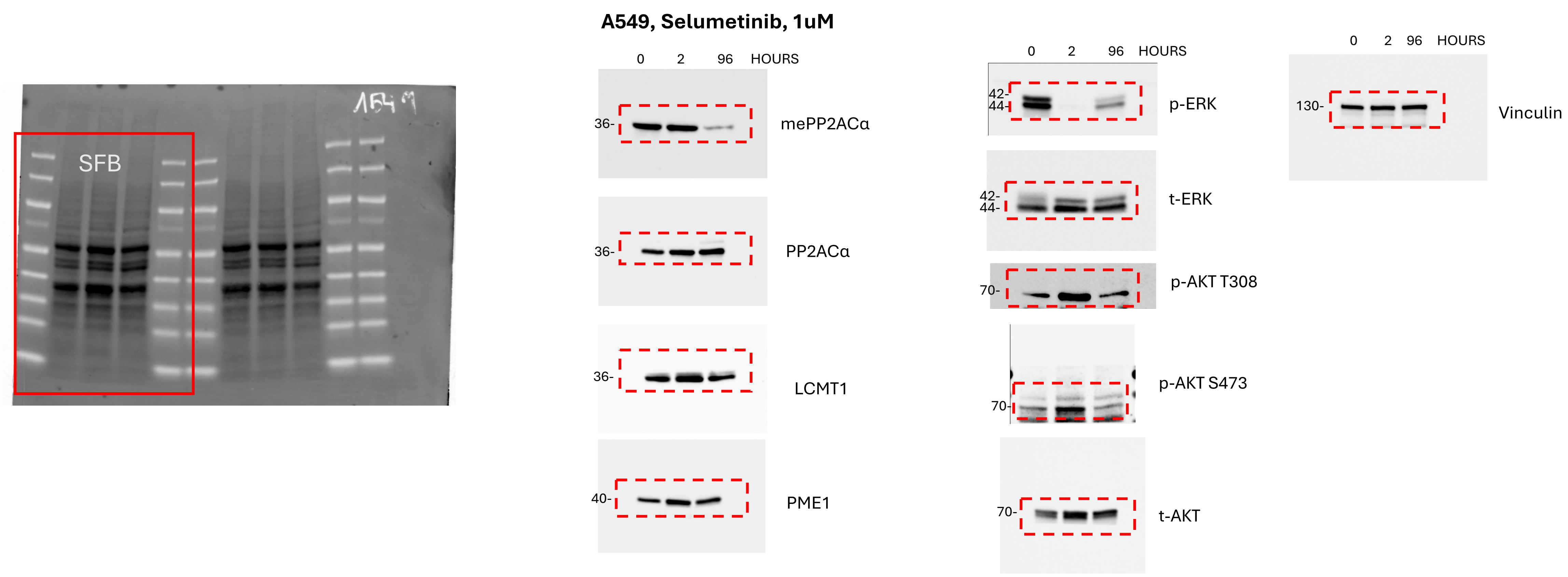

Full unedited blot/gel for Supplemental Figure 3A

Red boxes indicate the images used in Supplemental Figure 3A.

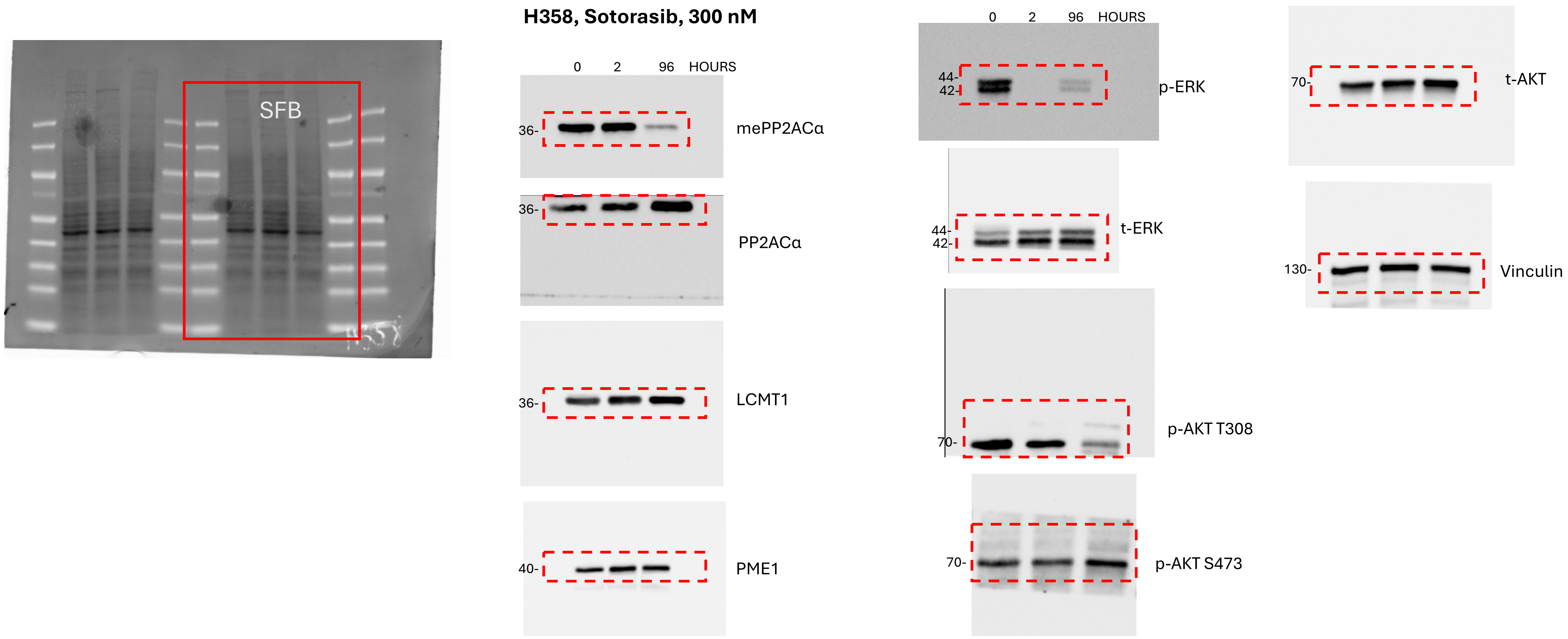

Full unedited blot/gel for Supplemental Figure 4A

Red boxes indicate the images used in Supplemental Figure 4A.

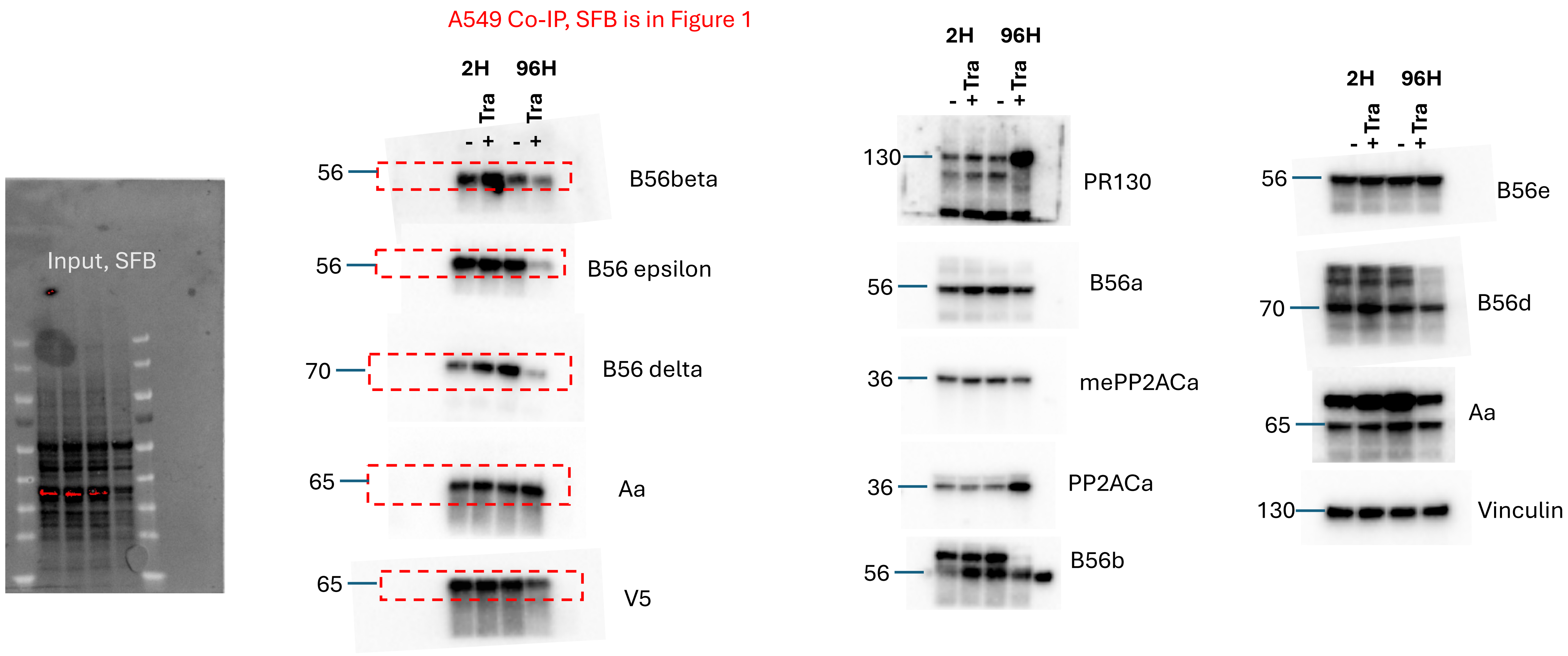

V5 appears in Figure 1 and SF4. It is shown again to complete the blot for the IP

Full unedited blot/gel for Supplemental Figure 4C

Red boxes indicate the images used in Supplemental Figure 4C.

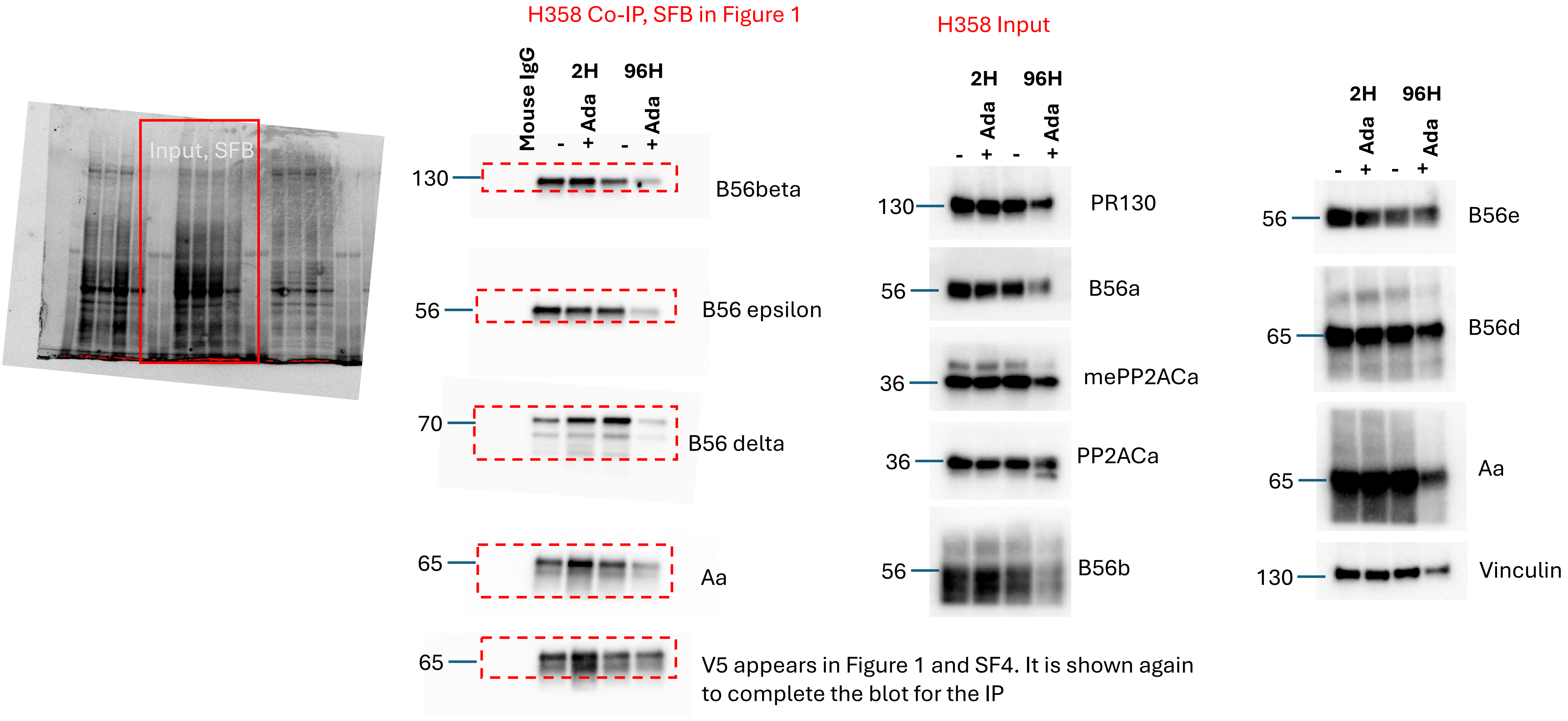

Full unedited blot/gel for Supplemental Figures 6 & 7

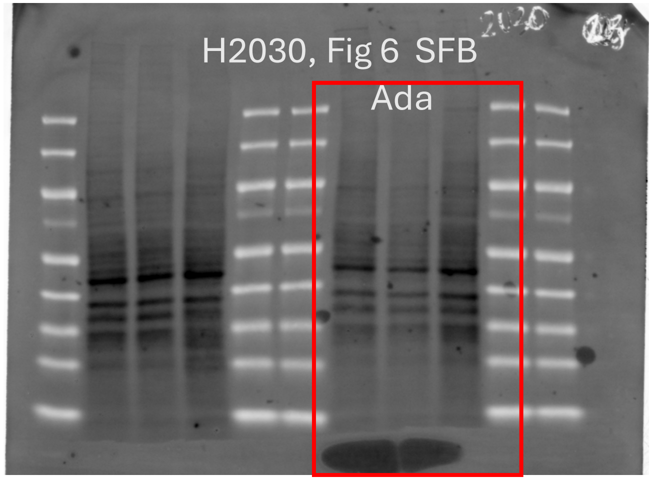

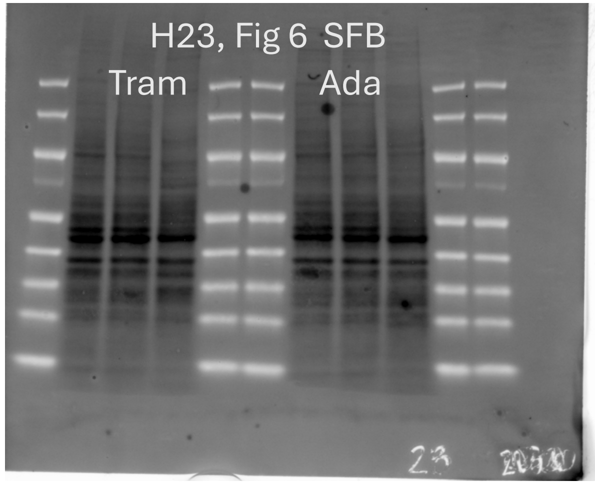

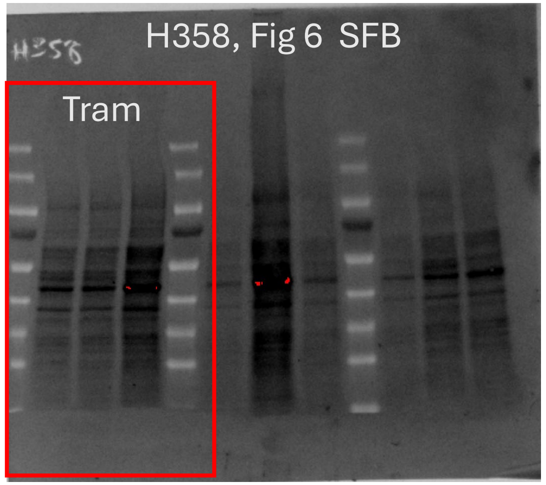

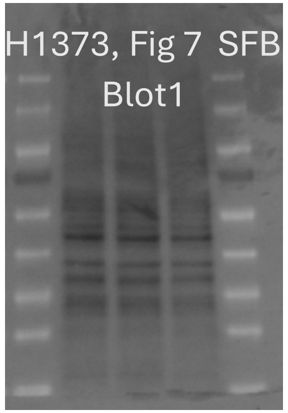

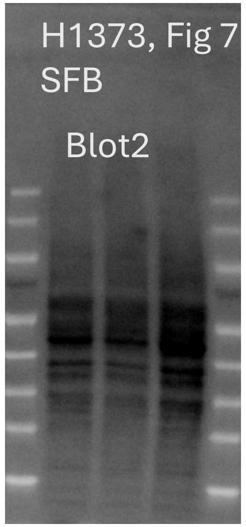

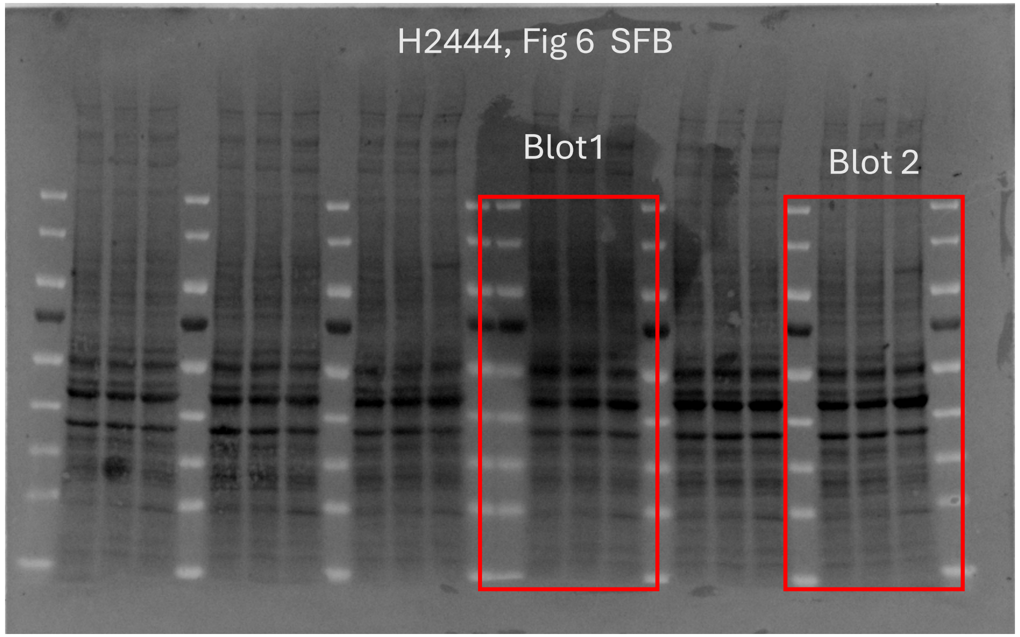

Full unedited blot/gel for Supplemental Figure 6A

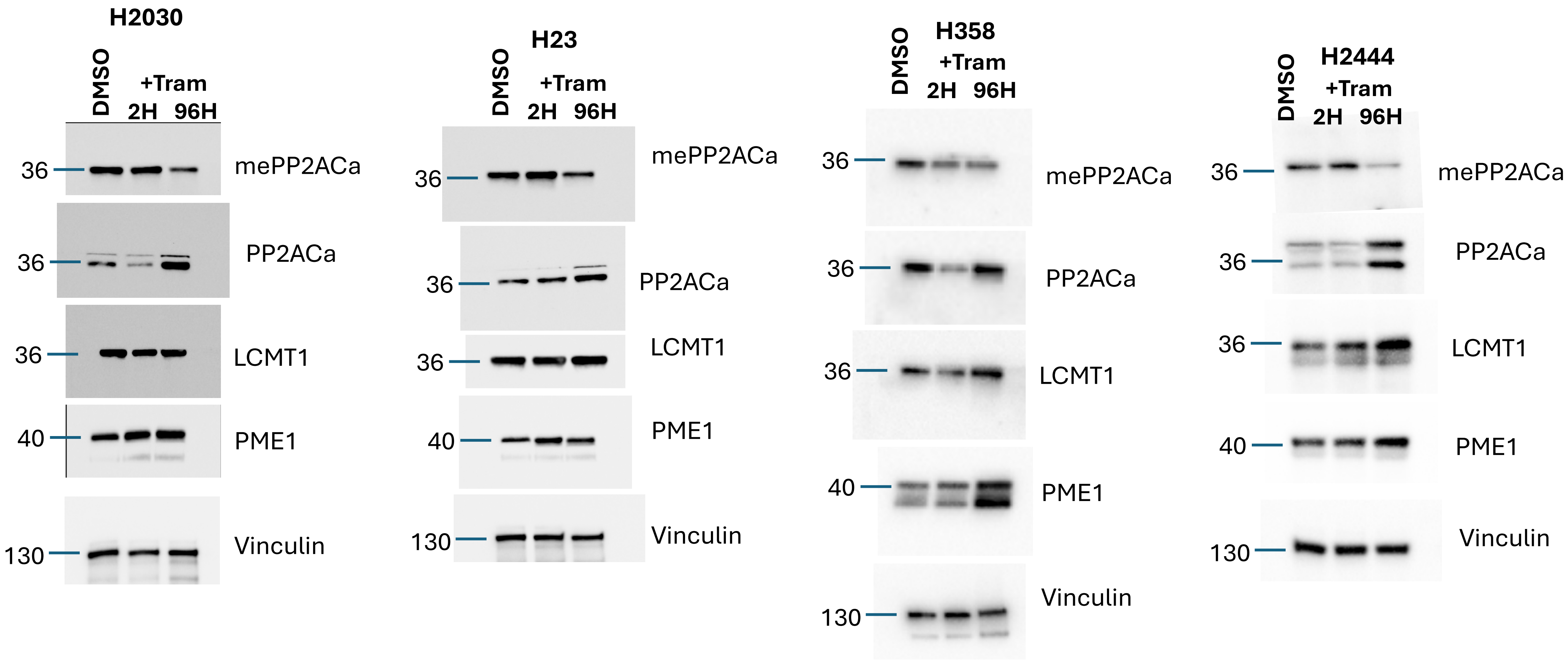

Full unedited blot/gel for Supplemental Figure 6B

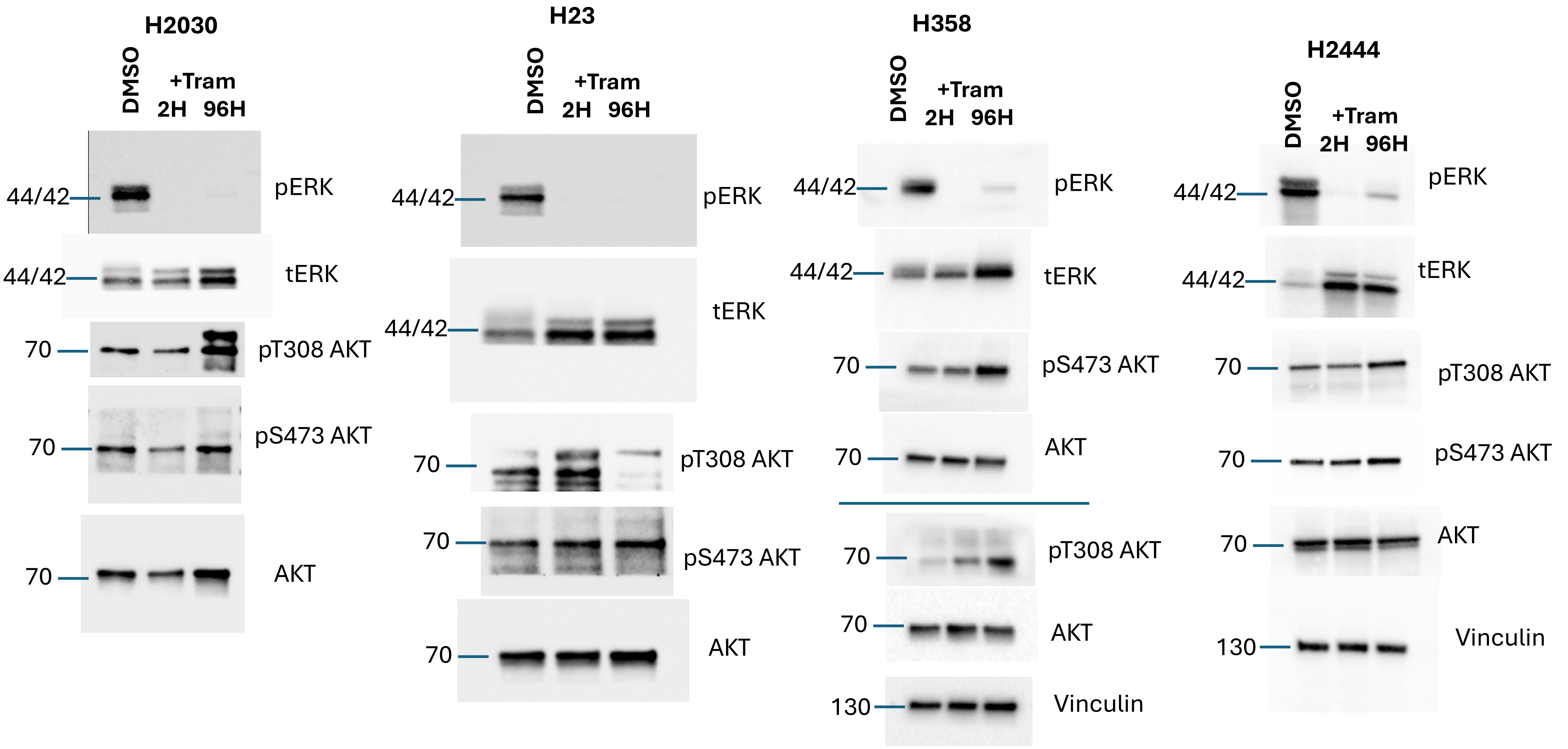

Full unedited blot/gel for Supplemental Figure 7A

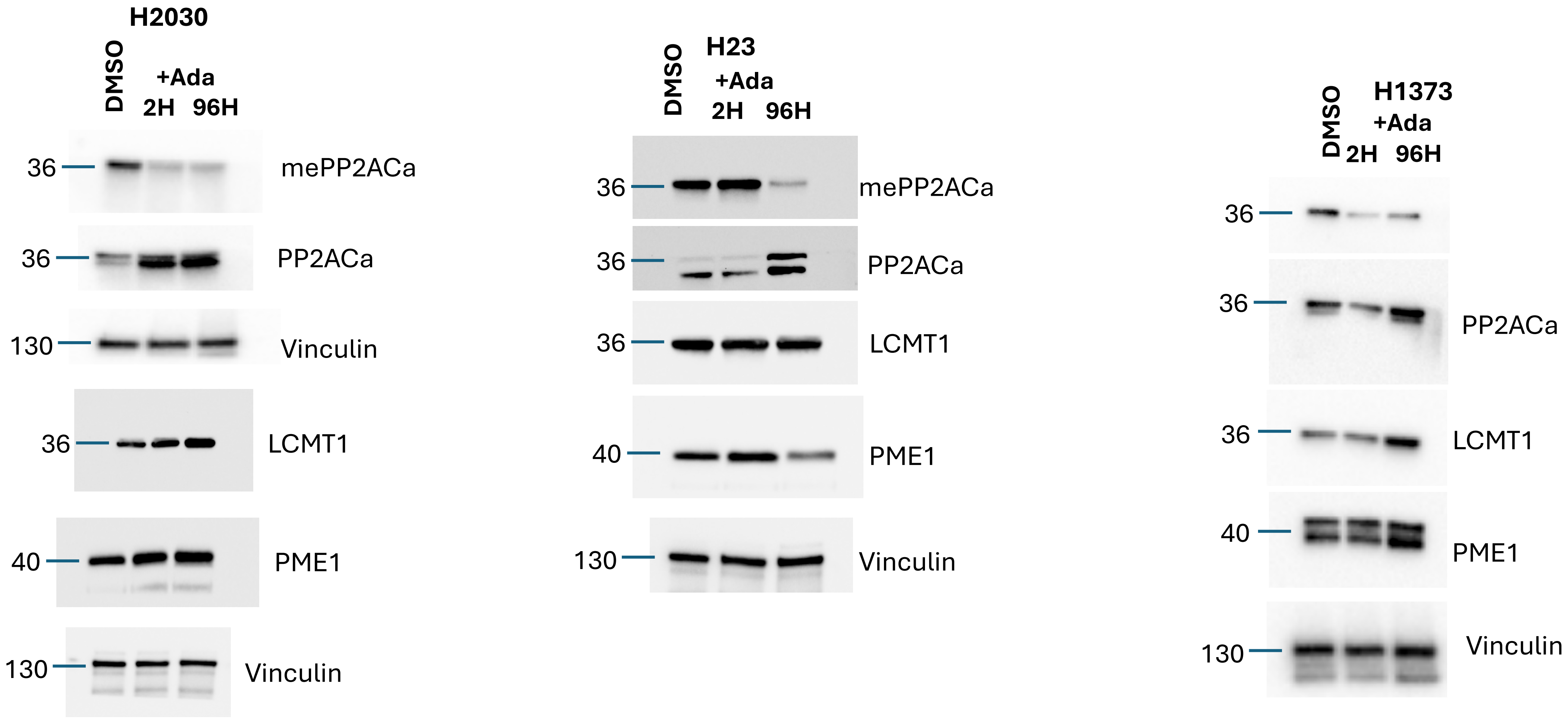

Full unedited blot/gel for Supplemental Figure 7B

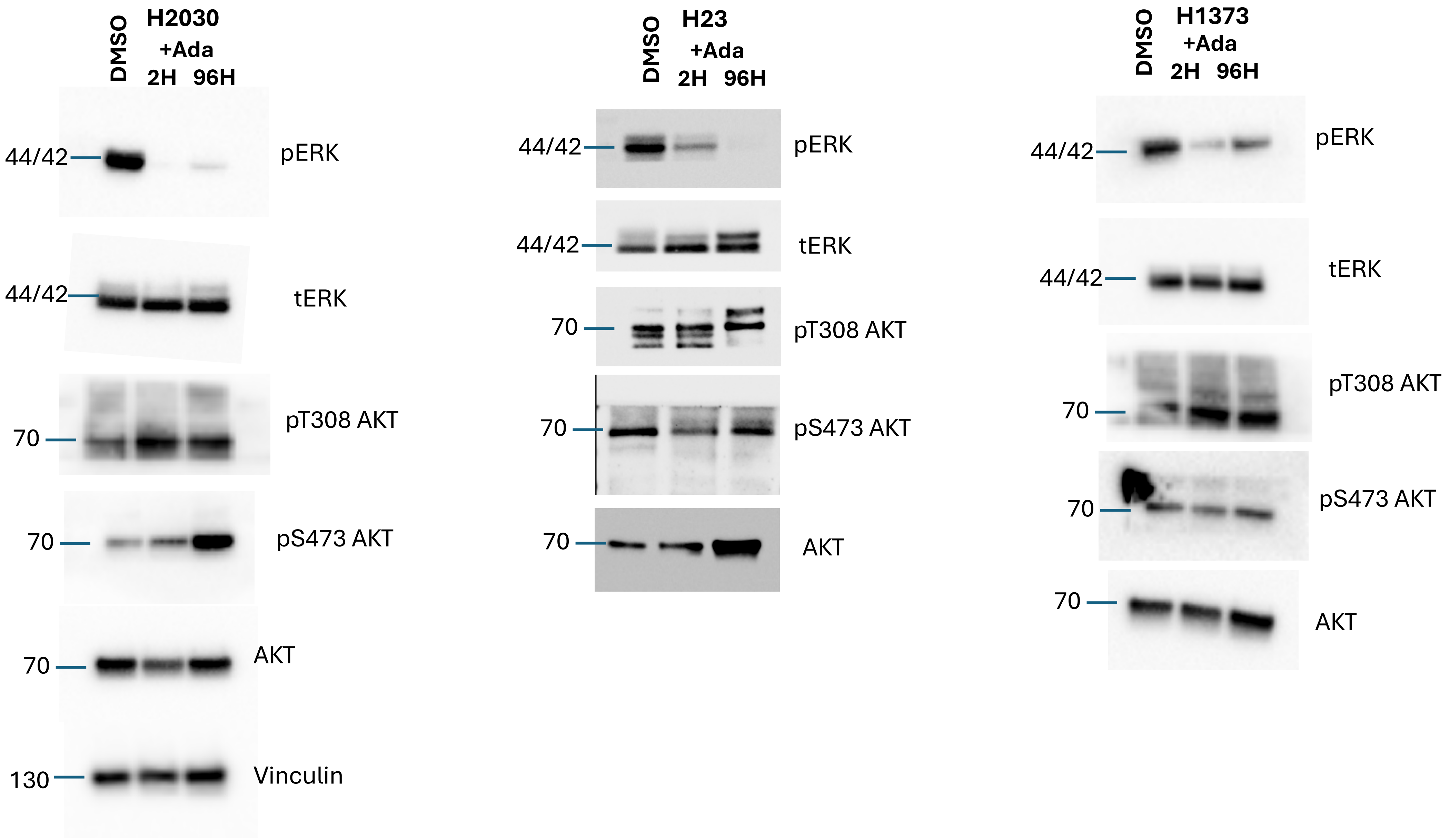

Full unedited blot/gel for Supplemental Figure 8G

Red boxes indicate the images used in Supplemental Figure 8G.

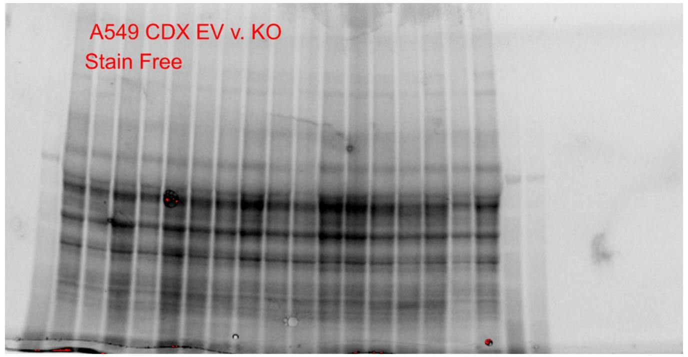

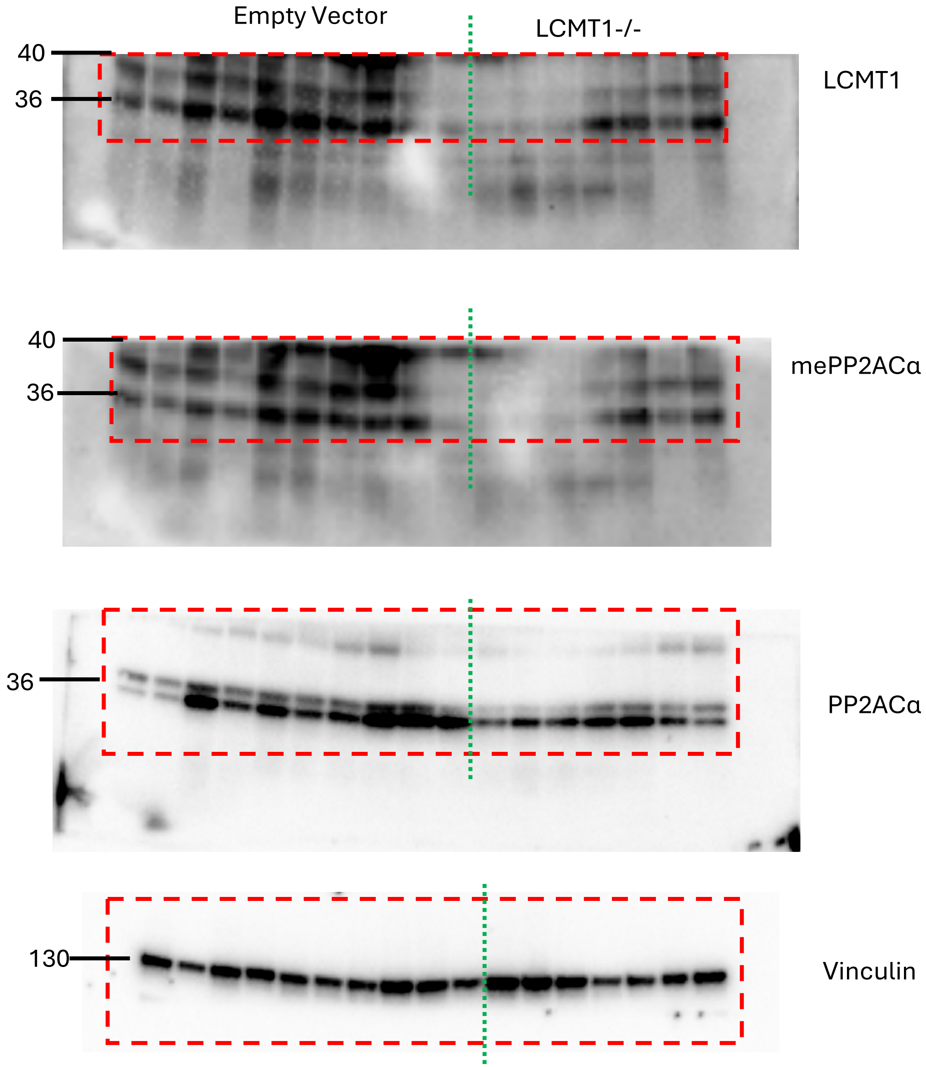

Full unedited blot/gel for Supplemental Figure 10, A and B

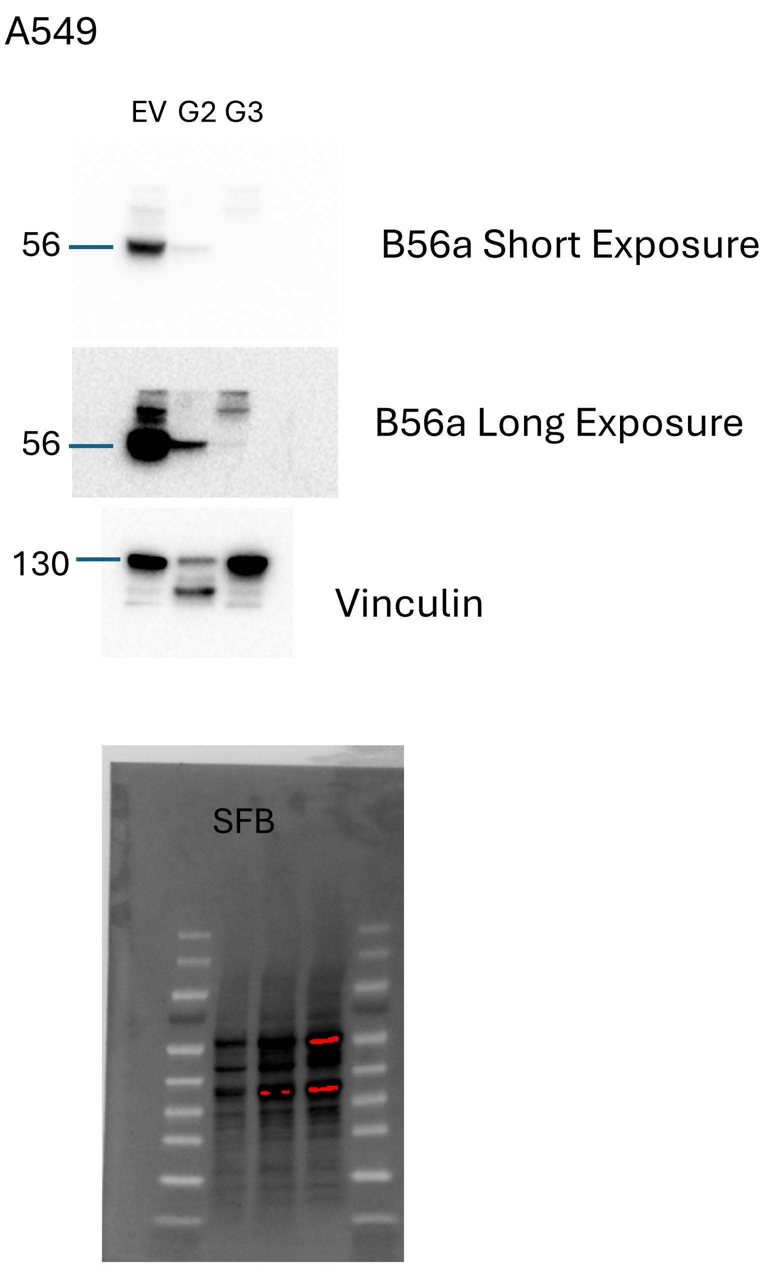

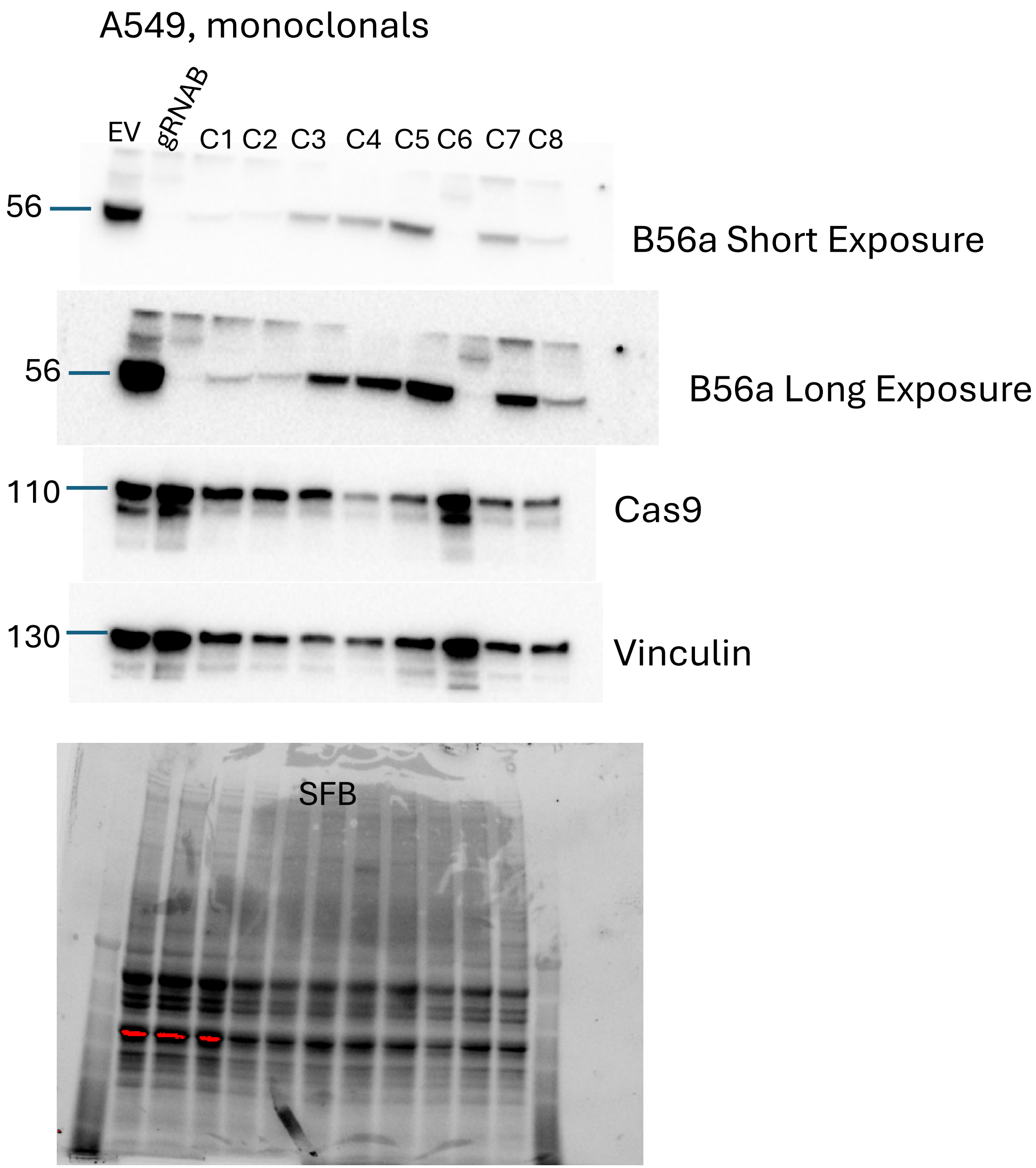

Full unedited blot/gel for Supplemental Figure 10, C and D

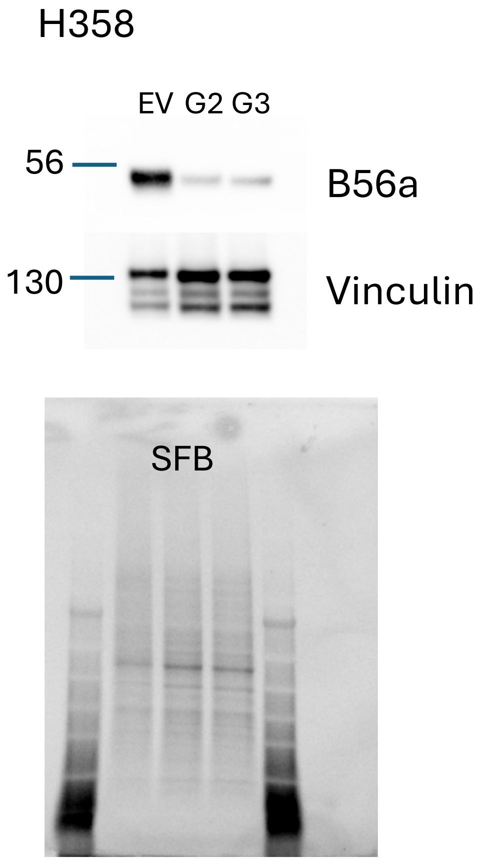

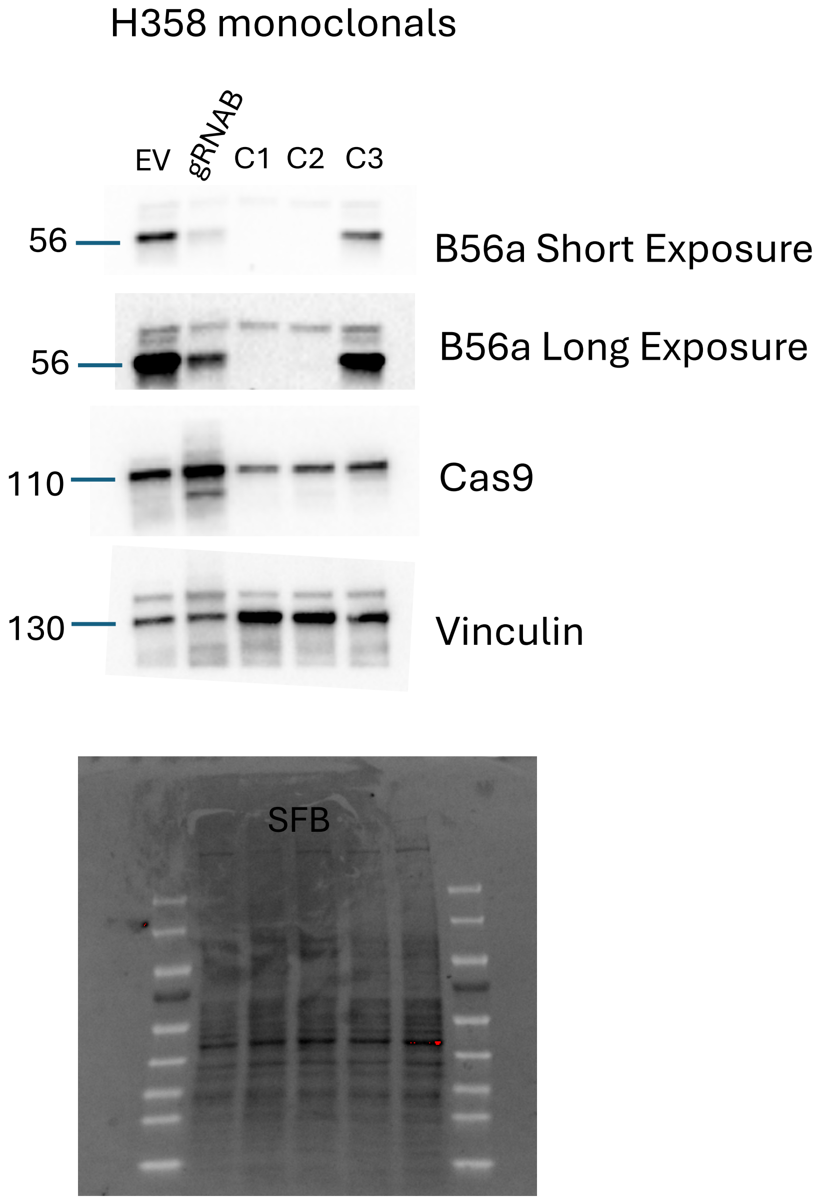

Full unedited blot/gel for Supplemental Figure 13F

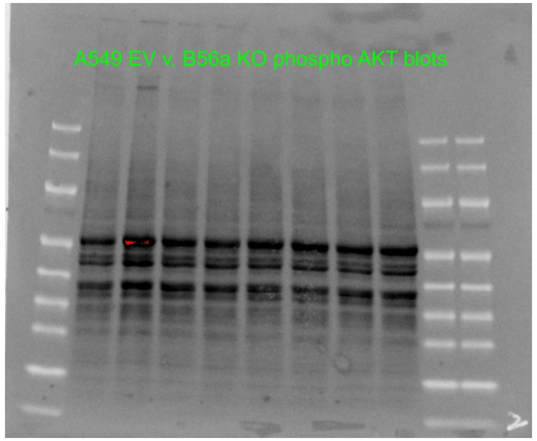

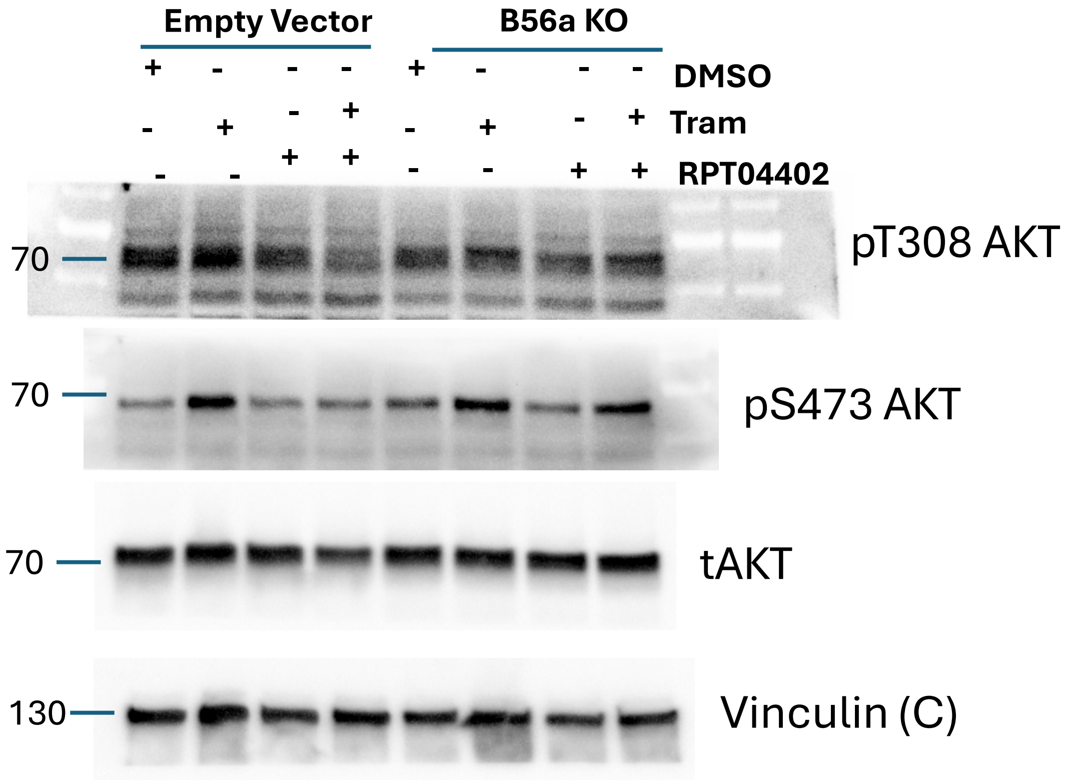

Full unedited blot/gel for Supplemental Figure 13G

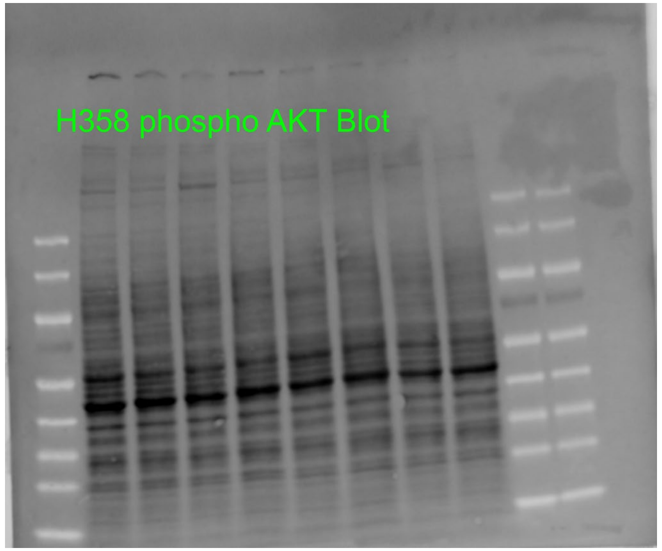

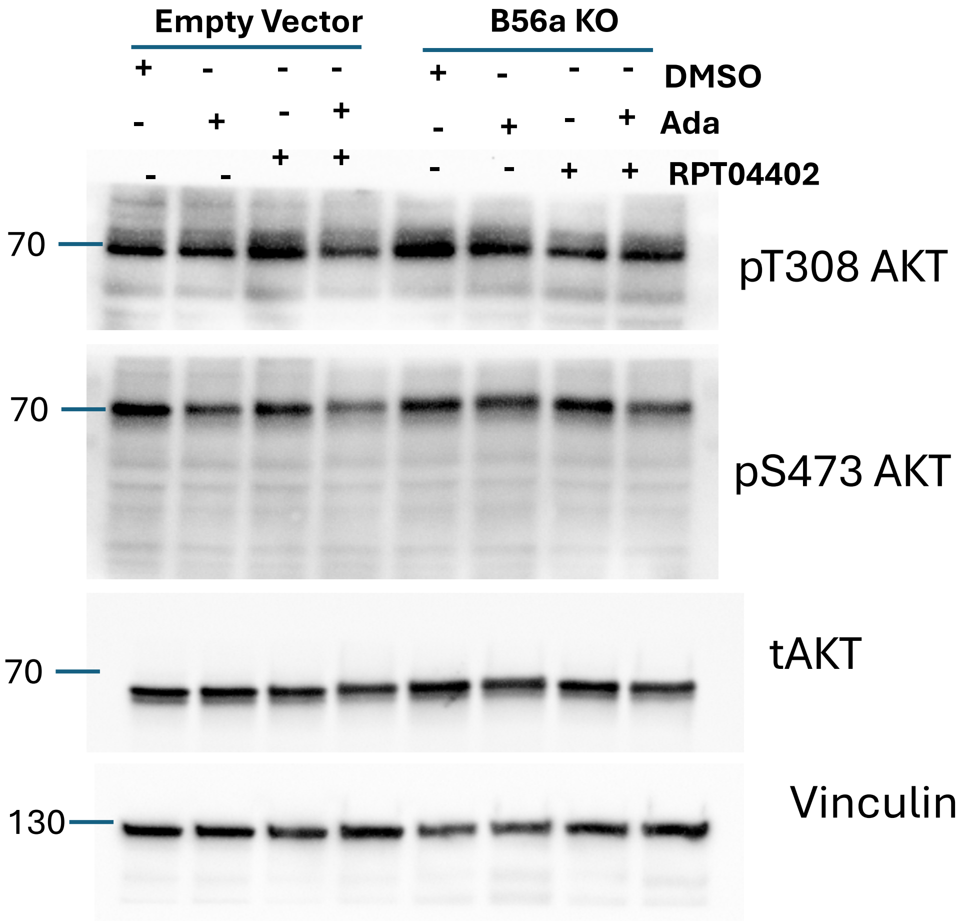

Full unedited blot/gel for Supplemental Figure 14F

Red boxes indicate the images used in Figure 14F.

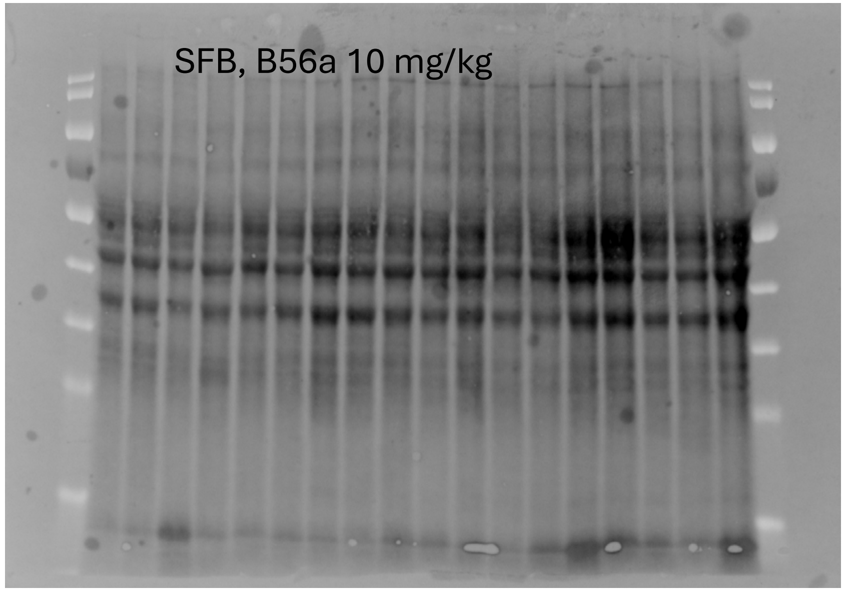

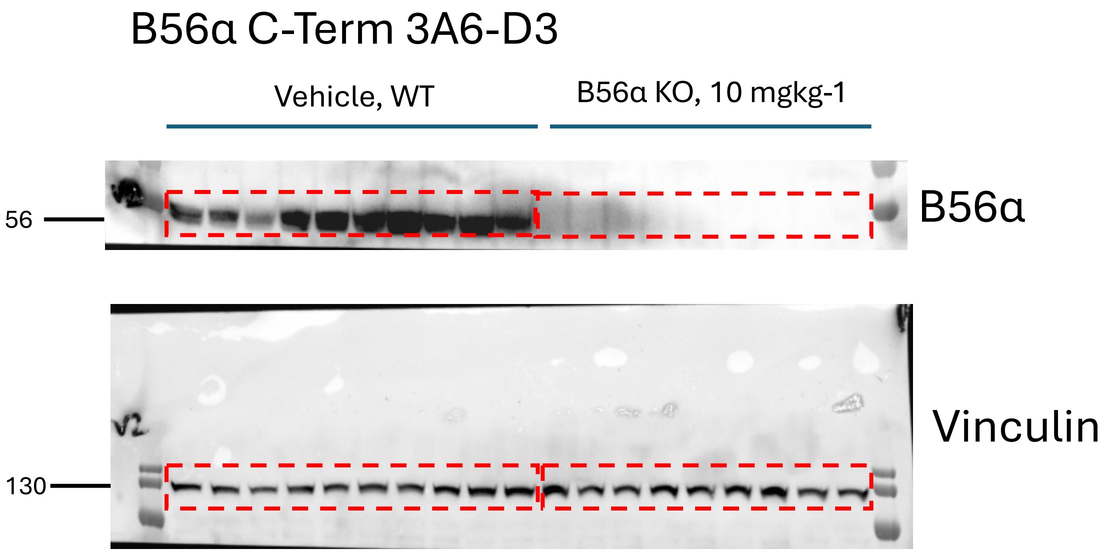

Full unedited blot/gel for Supplemental Figure 19C

Boxes indicate the images used in Figure 19C.

- Vehicle
- Trametinib
- RPT04402
- Combination

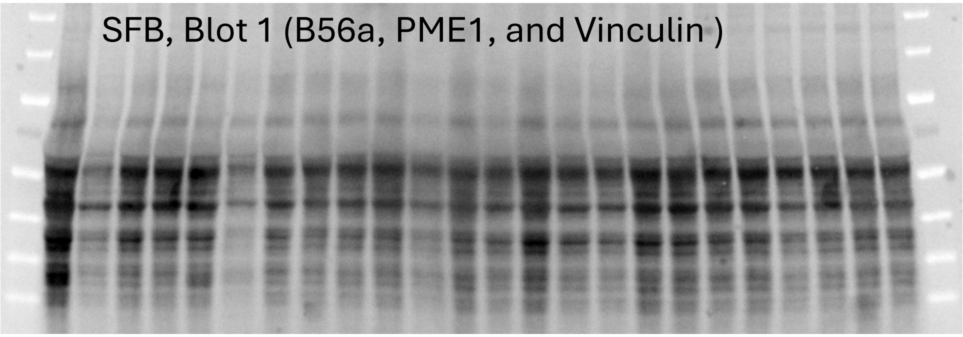

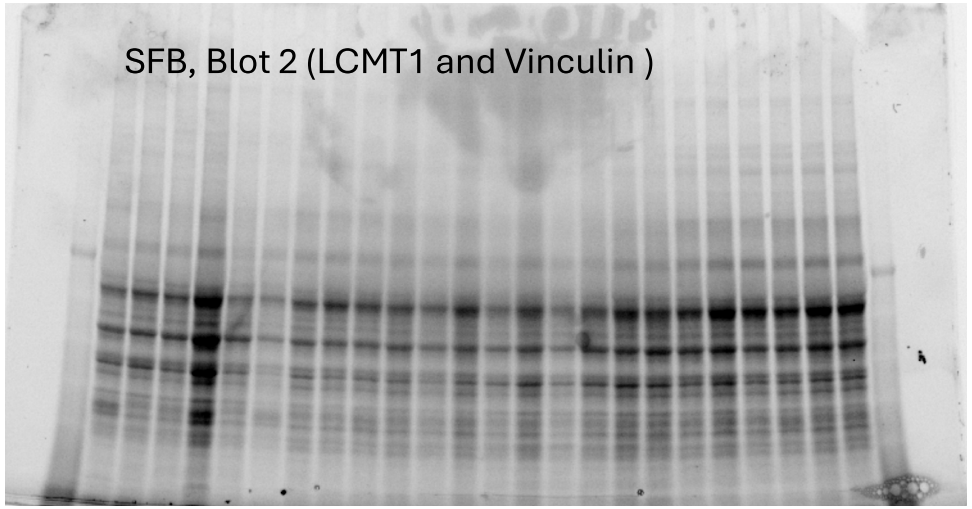

A549 PD, 3HR Post Final Dose

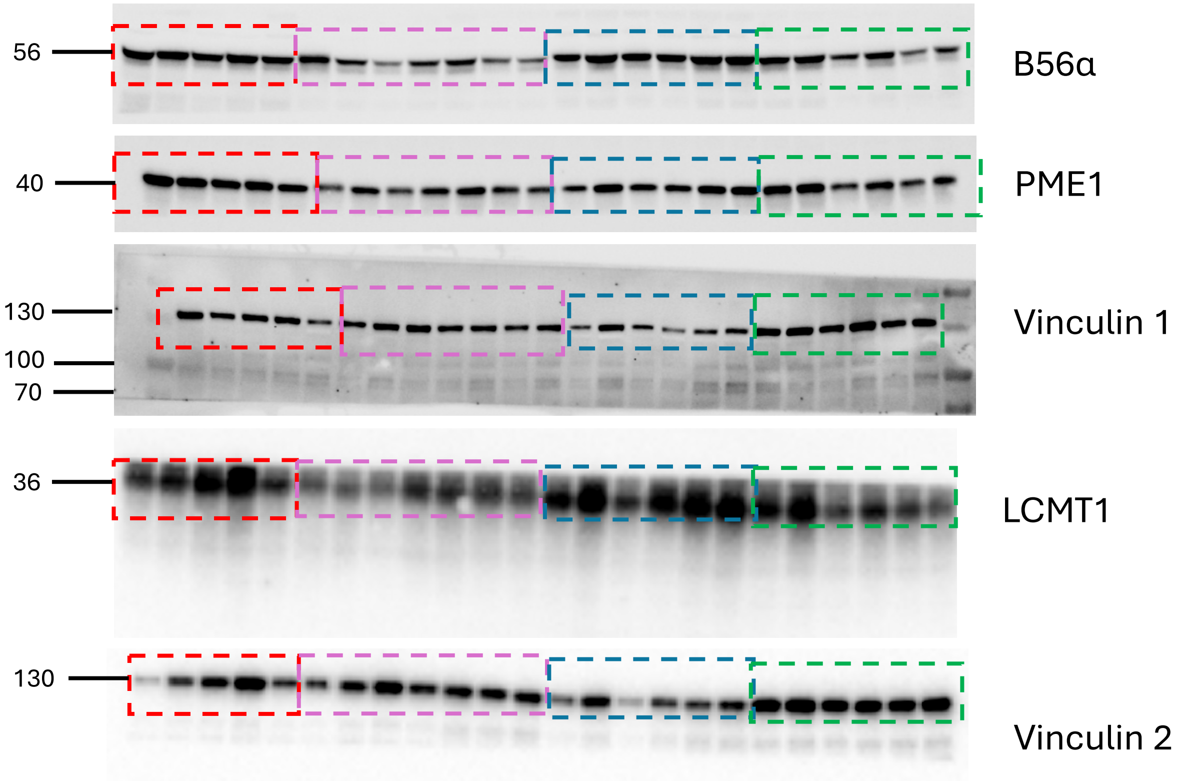

Full unedited blot/gel for Supplemental Figure 19D

Vehicle

Trametinib

RPT04402

Combination

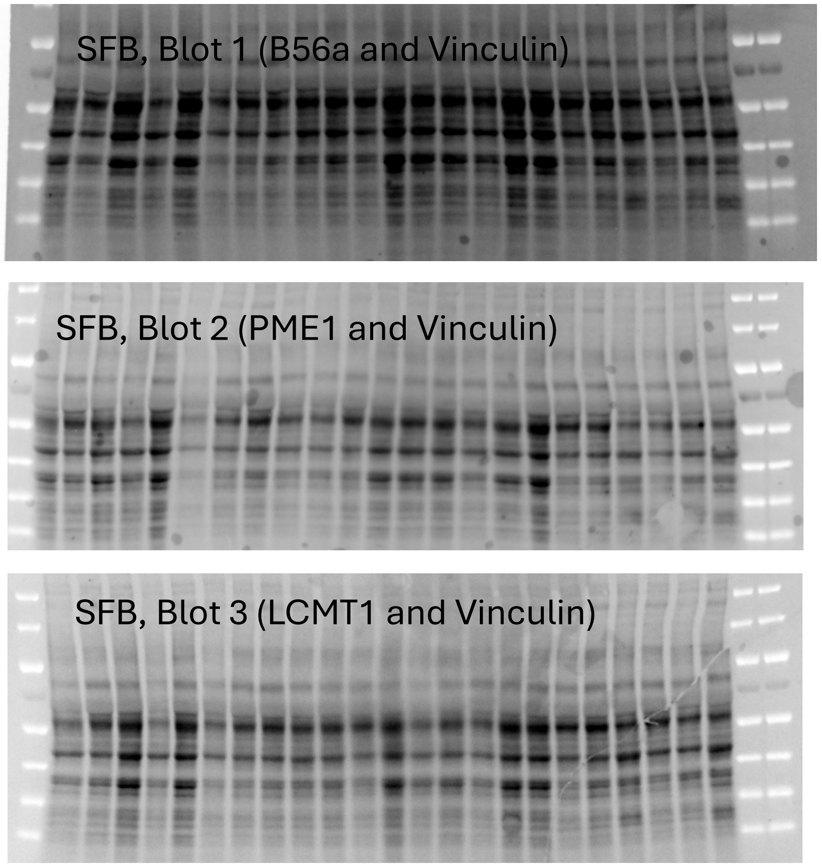

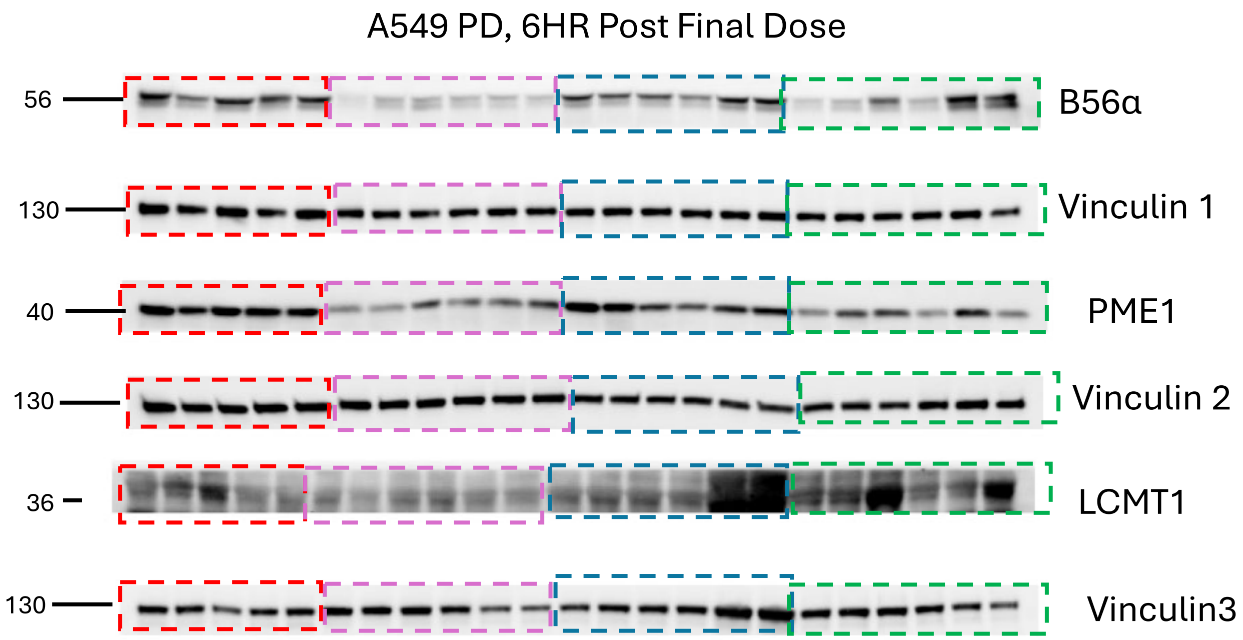

Full unedited blot/gel for Supplemental Figure 19E

Vehicle

Trametinib

RPT04402

Combination

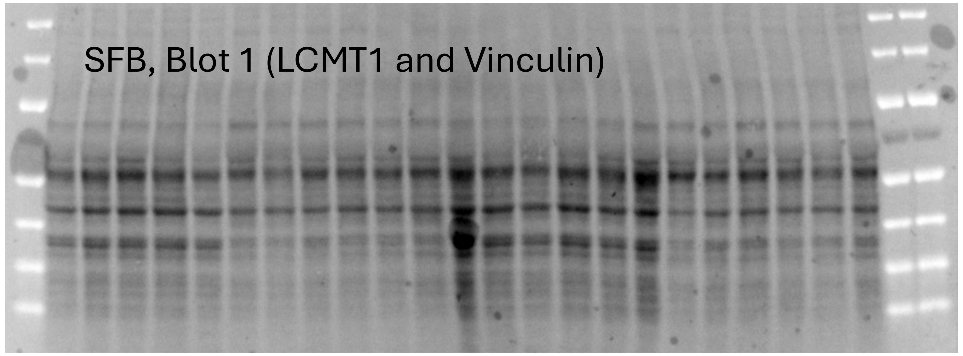

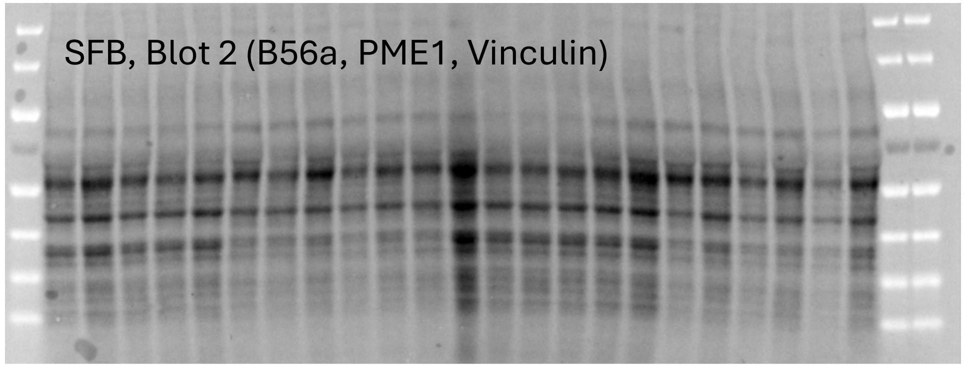

A549 PD, 24HR Post Final Dose

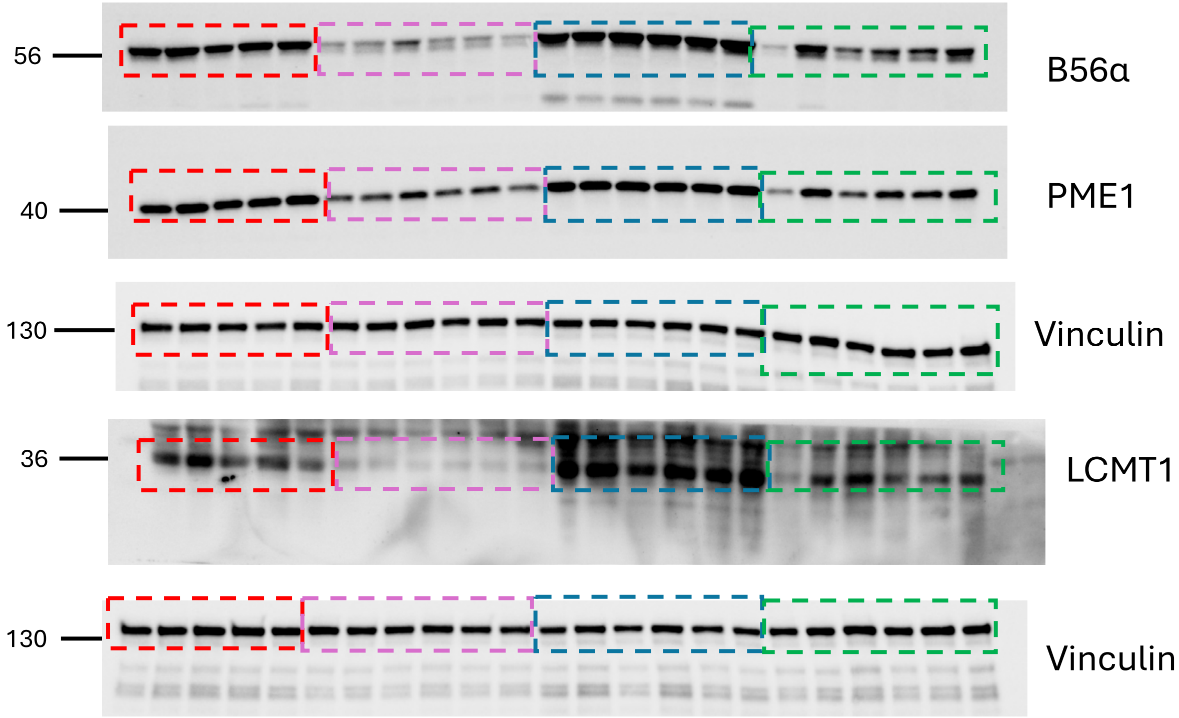

Full unedited blot/gel for Supplemental Figure 19F

Vehicle
Trametinib
RPT04402
Combination

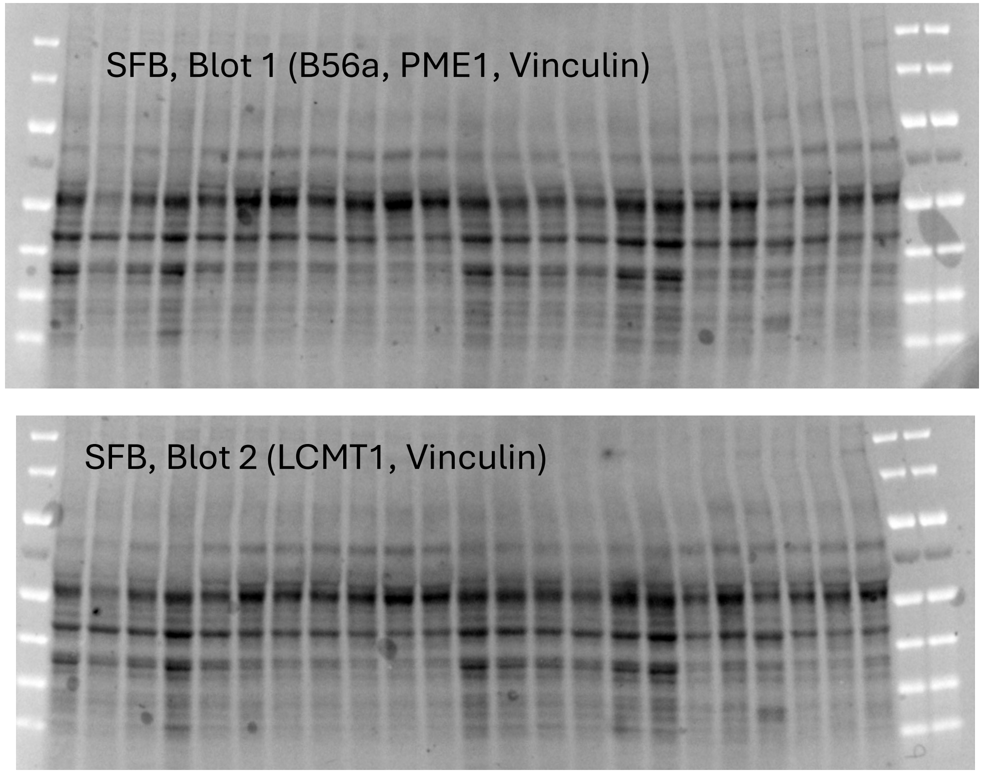

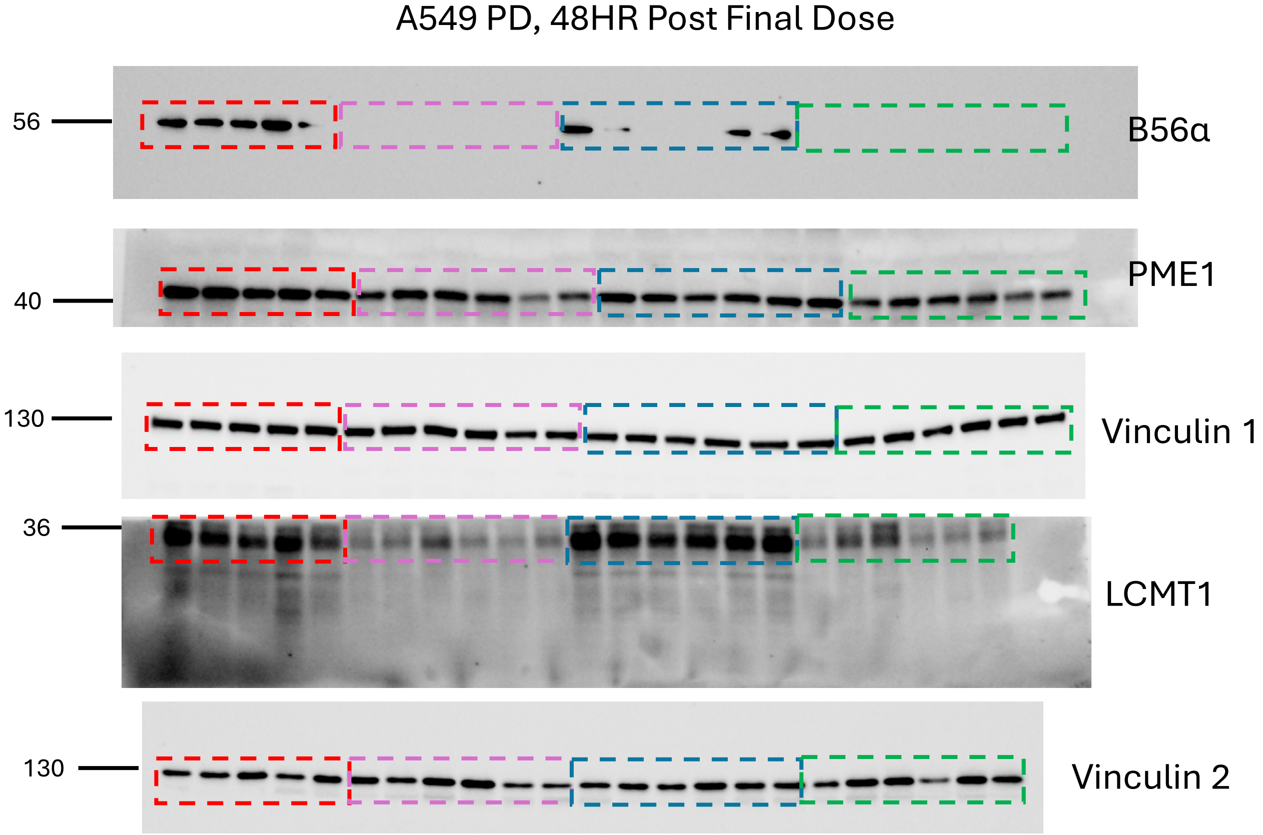

Full unedited blot/gel for Supplemental Figure 20A

Vehicle
Trametinib
RPT04402
Combination

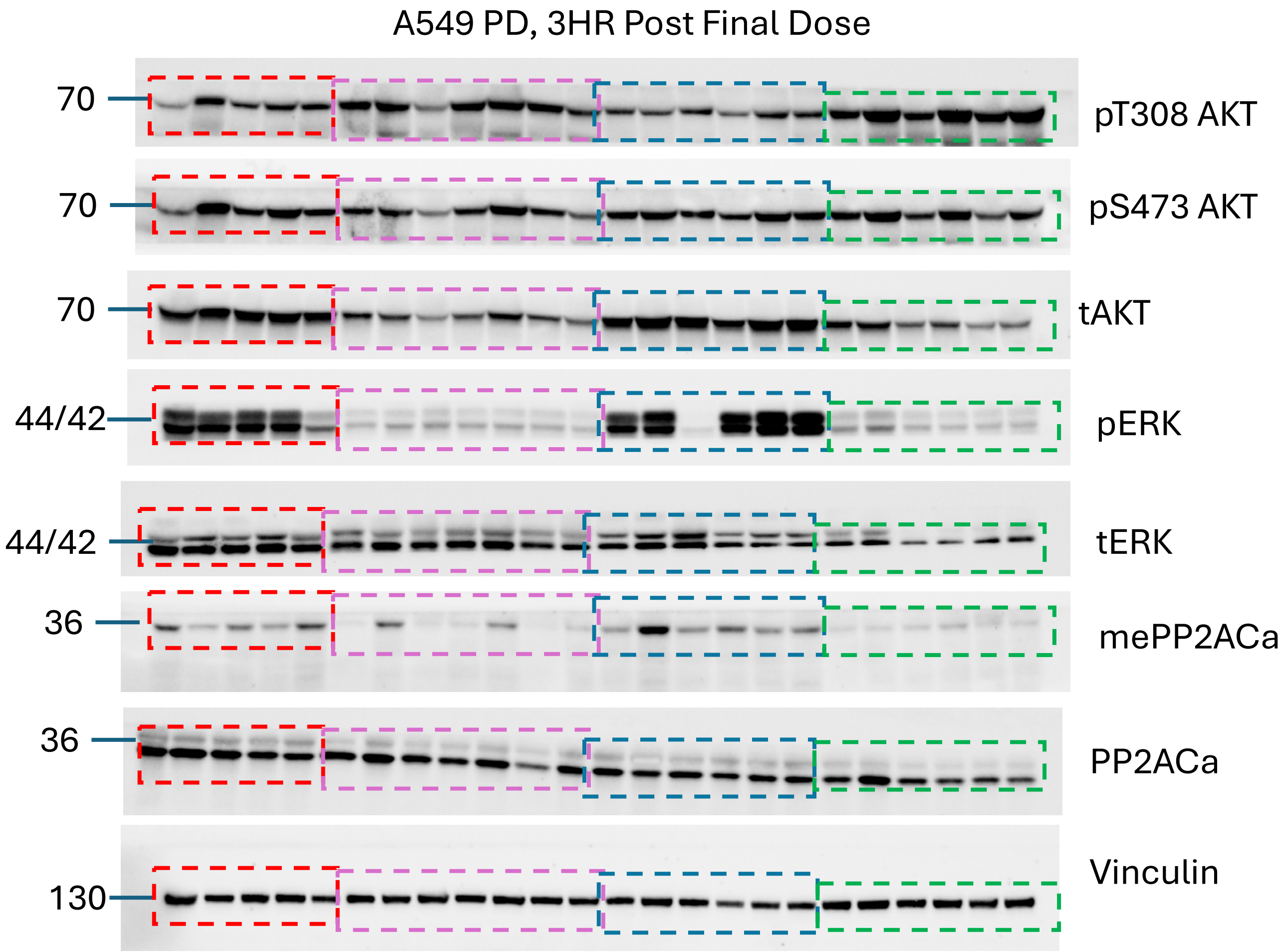

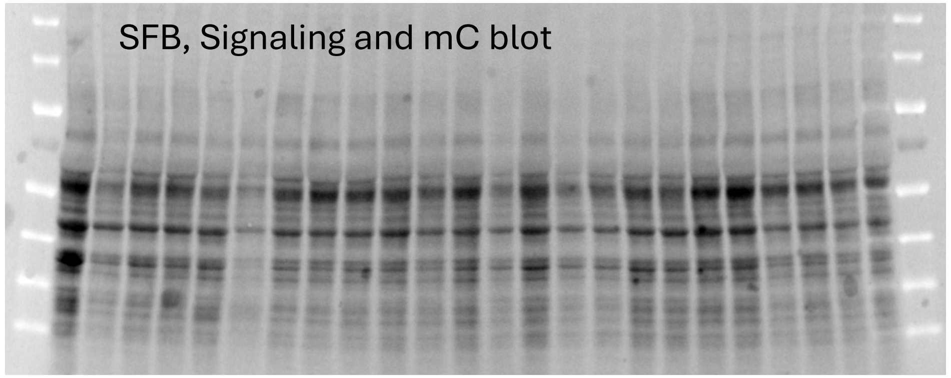

Full unedited blot/gel for Supplemental Figure 20B

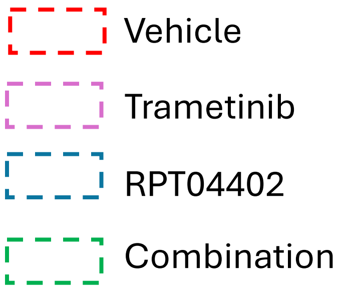

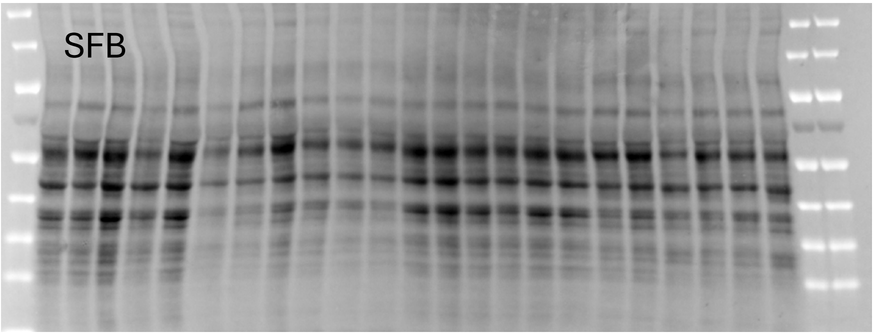

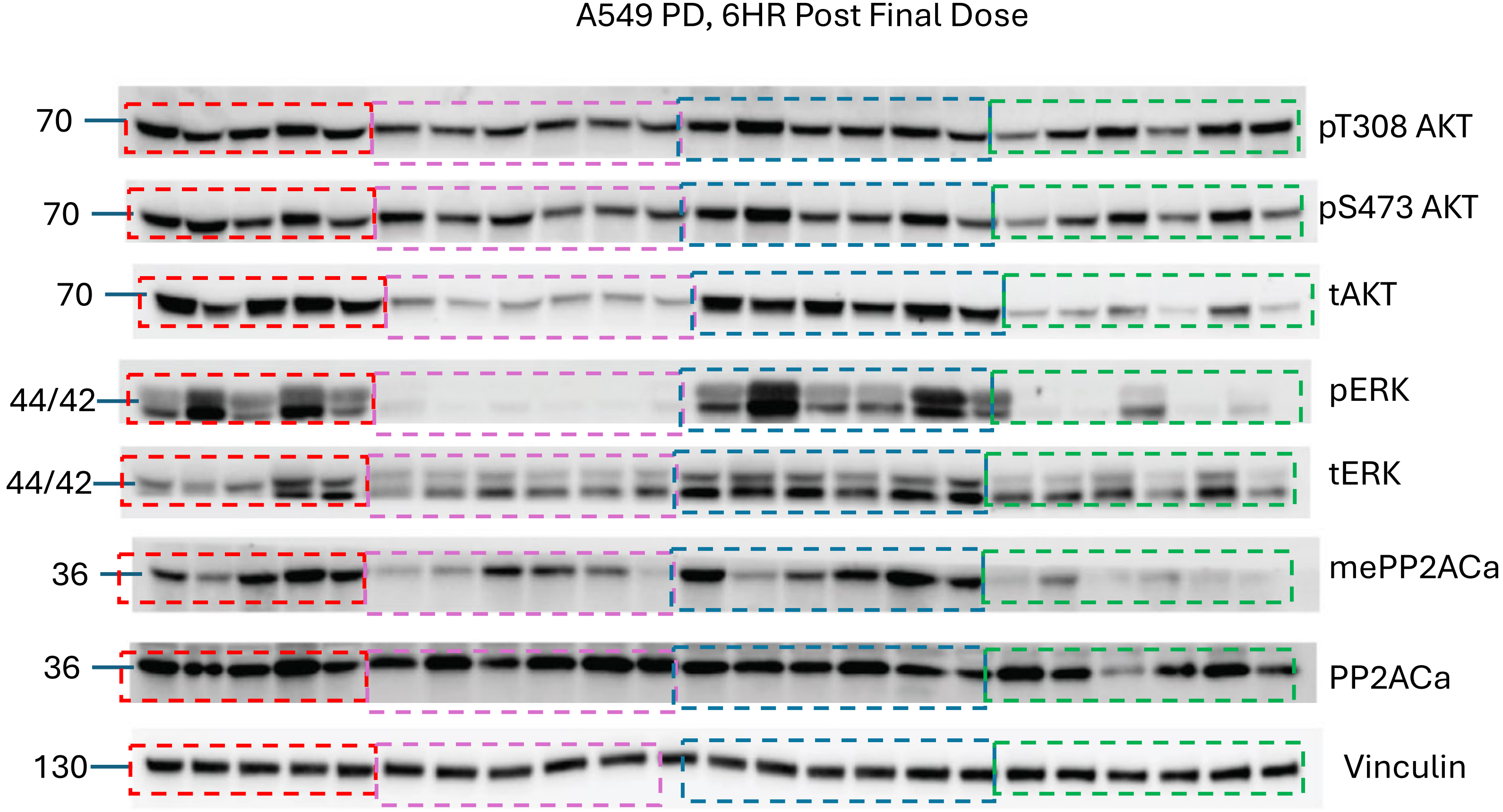

Full unedited blot/gel for Supplemental Figure 20C

Vehicle
Trametinib
RPT04402
Combination

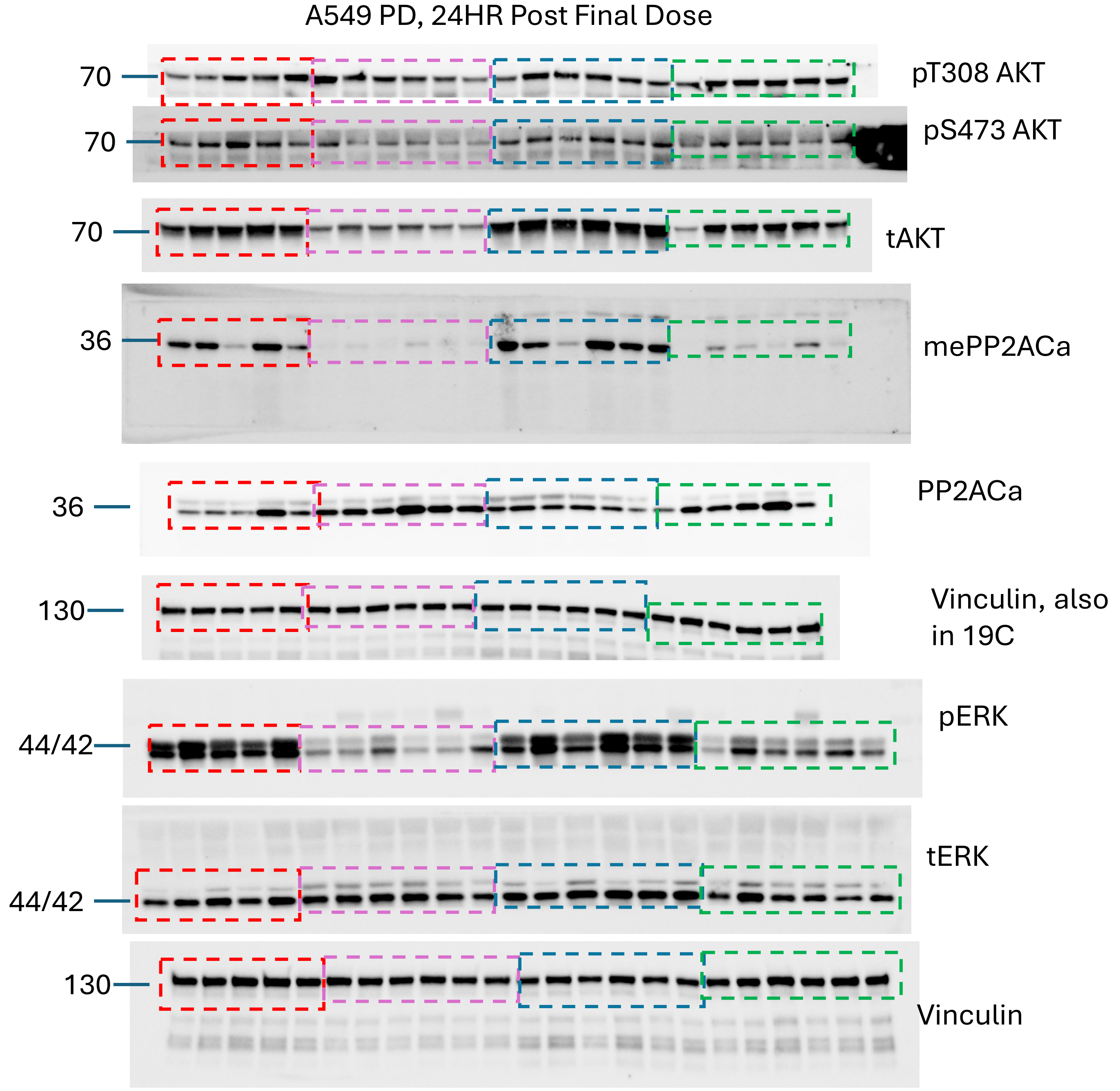

SFB Blot #1 is shared with 19C.
Here, includes mC, pAKT

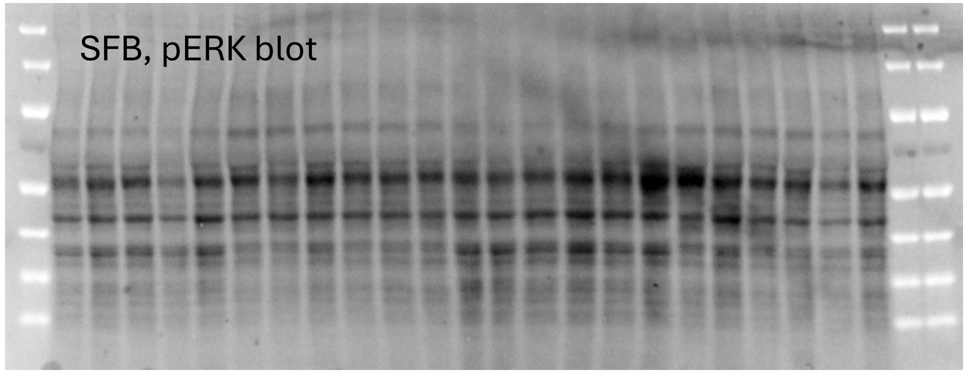

Full unedited blot/gel for Supplemental Figure 20F

Vehicle

Trametinib

RPT04402

Combination

SFB for pERK is the same as LCMT1 SFB in 19F

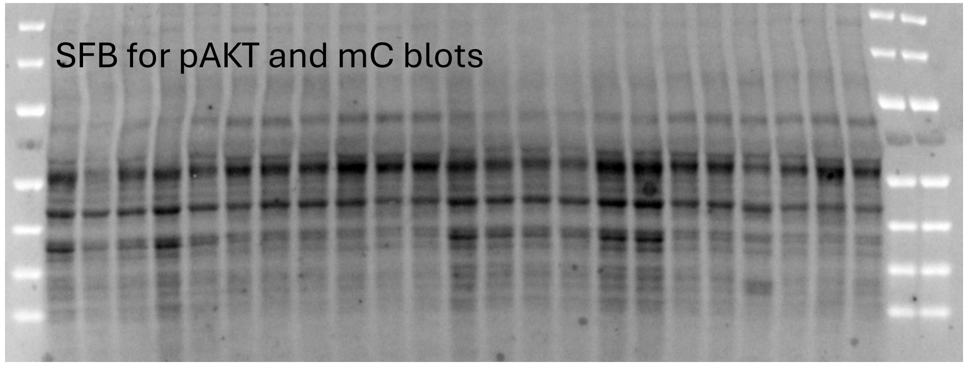

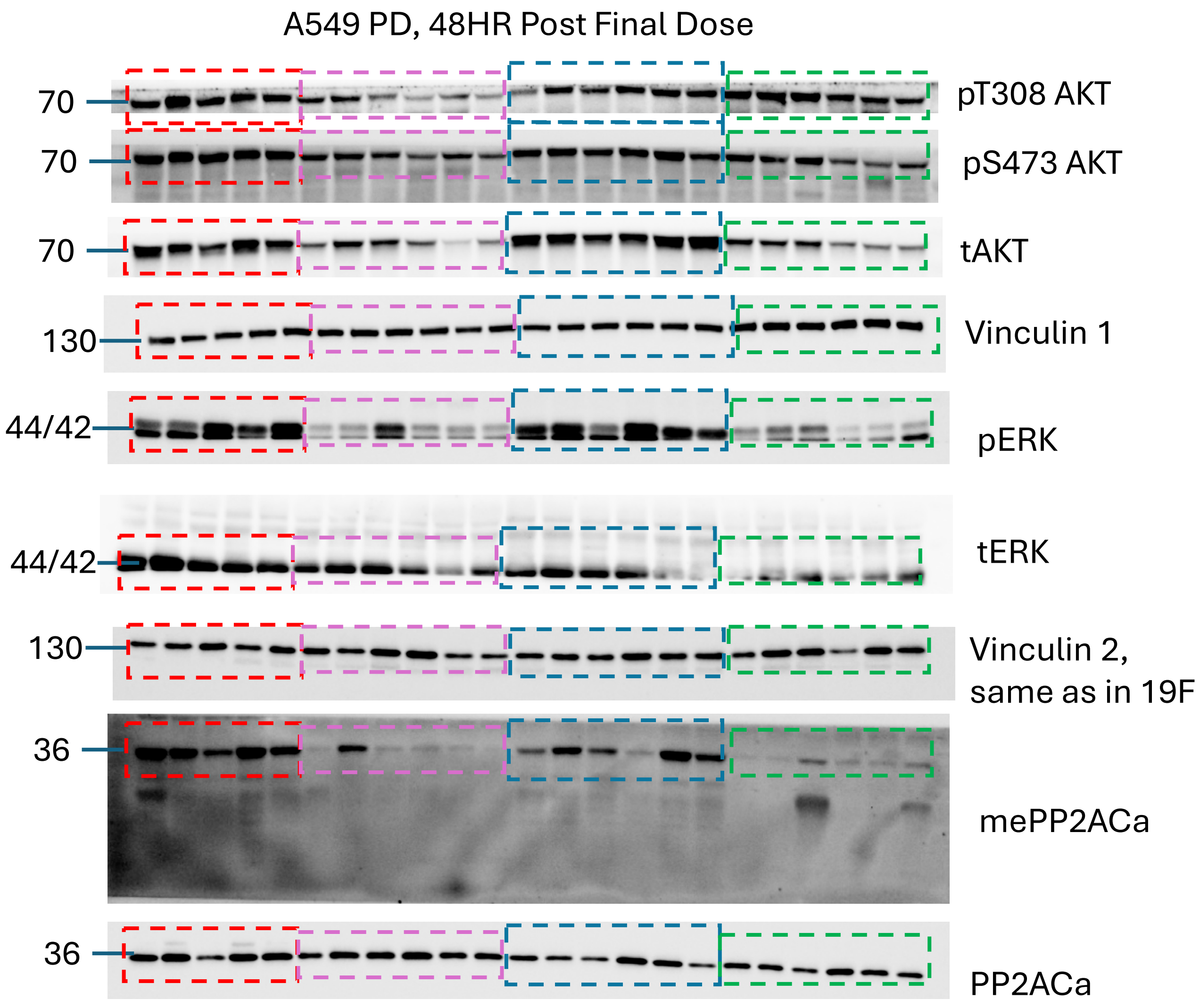

*Vinculin 1 corresponds to pAKT and mC, Vin 2 is for pERK blots
